# Data compilation on the effect of grain size, temperature, and texture on the strength of a single-phase FCC MnFeNi medium-entropy alloy

**DOI:** 10.1016/j.dib.2019.104807

**Published:** 2019-11-15

**Authors:** M. Schneider, F. Werner, D. Langenkämper, C. Reinhart, G. Laplanche

**Affiliations:** Institute for Materials, Ruhr-University Bochum, Universitätsstr. 150, 44801, Bochum, Germany

**Keywords:** FeNiMn, Medium- and high-entropy alloys, Compression-test data, Density and average thickness of annealing twins, Hall-Petch parameters

## Abstract

This data article presents a compilation of microstructural and mechanical data regarding the ternary single-phase FCC MnFeNi medium-entropy alloy (MEA). For the analysis, interpretation, and comparison of the data to literature values, the reader can refer to the original related research article entitled “Effect of Temperature and Texture on Hall-Petch Strengthening by Grain and Annealing Twin Boundaries in the MnFeNi Medium-Entropy Alloy”, see Schneider et al. (*Metals* 9, 2019, 84). The microstructural data reported here include: (i) raw backscatter electron (BSE) micrographs (tif-files) obtained using a scanning electron microscope (SEM) for nine different grain sizes with four images for each grain size and (ii) pdf reports and tables shown below presenting the distributions of the grain- (*d*, accounting for grain boundaries only) and crystallite- (*c*, which accounts for both grain and annealing twin boundaries) sizes and of the annealing twin thicknesses (*t*). These datasets may be useful to develop new algorithms for the automated evaluation of microstructural parameters in recrystallized alloys, i.e. with these benchmark data, an algorithm for image analysis could be trained to assess the above mentioned microstructural parameters. This would help to speed up the analysis of microstructures and improve its reliability. Additional tables describing the recrystallized microstructures and texture include the average number of annealing twin boundaries per grain (*n*), and the average Taylor factors (*M*). Raeisinia et al. (*Model. Simul. Mater. Sc.* 16, 2008, 025001) recently used a viscoplastic model to show that differences in the distribution of microstructural parameters affect the Hall-Petch parameters, but no attempt has been carried out so far to experimentally investigate this possibility since grain size distributions are rarely reported. Here, our benchmark data (e.g. distribution in grain/crystallite sizes, annealing twins per grain, distribution of annealing twin thicknesses) could be used to address these issues.

The data describing the mechanical properties reported here are excel-sheets of raw stress-strain curves for temperatures ranging from 77 K to 873 K and different grain sizes. The yield stress (*σ*_*0.2%*_) and the normalized Hall-Petch parameters (*σ*_*0*_/*G* and *k*_*y*_/*Gb*^2^) are given for all temperatures. The normalized Hall-Petch parameters are reported here since they allow to better compare the strength and the magnitude of grain boundary strengthening of different alloys with the same crystallographic structure, see Cordero et al. (*Int. Mater. Rev.* 61, 2016, 495–512). Moreover, the Hall-Petch parameters as well as the mechanical data reported here could be used for data mining and implemented in programs used for alloy design.

**Table:** Specifications Table

Subject	Materials Science
Specific subject area	High- and medium-entropy alloys (HEAs and MEAs)
Type of data	Tables (microstructural parameters and Hall-Petch parameters)/Excel-sheets (raw stress-strain curve data), Images (scanning electron microscopy), pdf-files (assessment of grain and crystallite sizes using the lineal intercept method)
How data were acquired	SEM: Quanta FEI 650 ESEM; Tensile/Compression testing machine: Zwick Roell XForce Z100
Data format	Raw (stress-strain curves, images), analyzed (grain/crystallite sizes, average annealing twin thicknesses, Taylor factors, Hall-Petch parameters)
Parameters for data collection	Backscatter electron images were obtained using an SEM of type Quanta FEI 650 ESEM with acceleration voltages between 15 kV and 30 kV and a working distance of 10 mm. Compression tests were performed at eight different temperatures with a constant strain rate of 10^−3^ s^−1^. Assessment of grain and crystallite sizes was carried out using the Heyn lineal intercept method.
Description of data collection	Metallographic samples were cut, embedded and prepared by grinding and polishing.
Data source location	Institute for Materials, Ruhr-University Bochum, Universitätsstr. 150, 44801 Bochum, Germany
Data accessibility	Data are with the article (attached file)
Related research article	Schneider, M., Werner, F., Langenkämper, D., Reinhart, C., Laplanche, G., 2019. Effect of Temperature and Texture on Hall-Petch Strengthening by Grain and Annealing Twin Boundaries in the MnFeNi Medium-Entropy Alloy. Metals. 19, 84. https://doi.org/10.3390/met9010084 [1].

**Table:** 

**Value of the Data** • High-quality datasets regarding recrystallized microstructures and mechanical properties of the ternary MnFeNi medium-entropy alloy are reported here. These data may be useful for other researchers in the community of high- and medium-entropy alloys. • This data compilation (BSE micrographs, Tables and pdf-files reporting the grain/crystallite-size distributions, Tables presenting the size distribution of the annealing twin thicknesses and Tables where the density of annealing twins as well as the texture are reported) can be used for the development of algorithms for image analysis to further improve the automated analysis of microstructures. • Our stress-strain curves could be used to further improve the automated analysis of yield stresses (machine learning). • The normalized Hall-Petch parameters reported here (correlation between yield stresses and grain/crystallite sizes) could be useful for other researchers who are interested in how these parameters are affected by chemistry, microstructure (*especially grain size distribution*), and alloy parameters such as the stacking fault energy and the shear modulus

## 1 Data

**Fig. 1:**
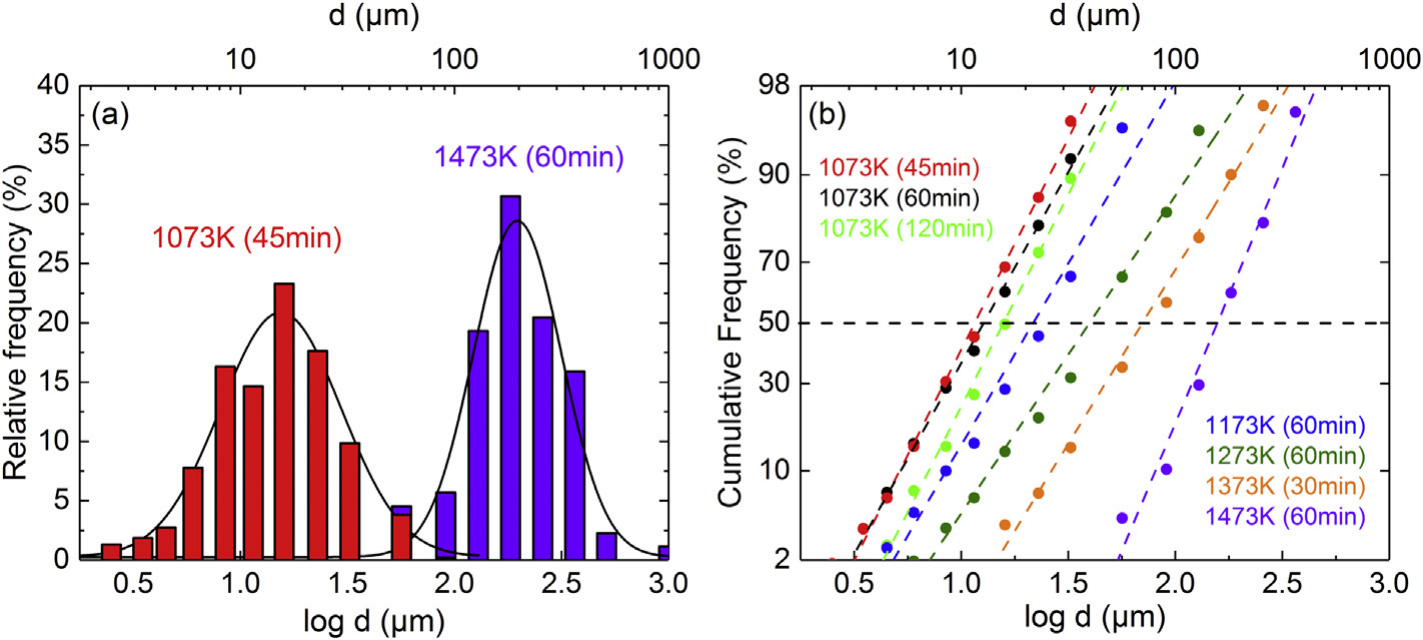
(a) Histograms comparing the grain size distributions of the specimens with the smallest (*d* = 17 μm, 1073 K for 45 min) and the biggest grain size (*d* = 216 μm, 1473 K, 60 min). (b) Logarithmic cumulative probability plots after annealings at different temperatures [1073 K–1473 K] and times [45 min–120 min].

**Fig. 2:**
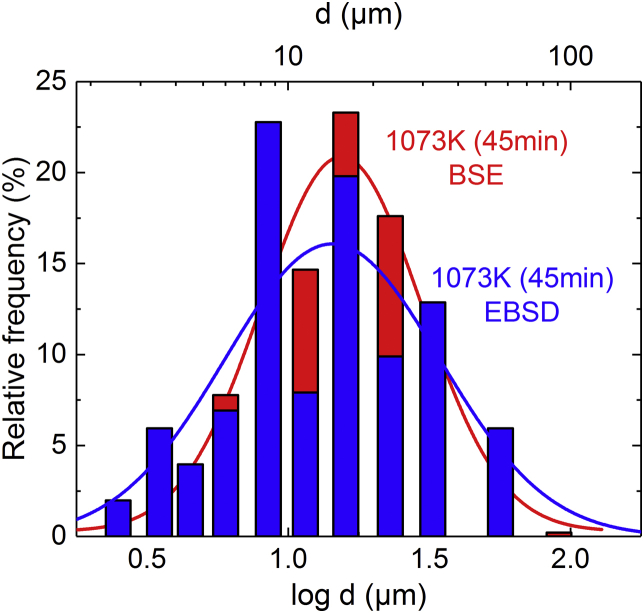
Comparison of the grain size distributions obtained by two different methods for the microstructure with the smallest grain size (*d* = 17 μm, 1073 K for 45 min). The blue histogram and curve represent the data obtained by EBSD whereas the red ones were obtained using the Heyn lineal intercept method in combination with BSE micrographs.

High- and medium-entropy alloys are currently intensively studied by the materials-science community [[1], [2], [3], [4], [5], [6], [7], [8]]. However, raw data are rarely reported in the literature which precludes data mining for alloy development, see Ref. [9]. The data presented in this article are microstructural and mechanical data for the single-phase FCC MnFeNi medium-entropy alloy. Recrystallization heat treatments at temperatures lying in the range (1073 K–1473 K) for times between 45 min and 120 min yielded nine different recrystallized microstructures. Four BSE-micrographs were recorded for each heat treatment. Since most of the BSE micrographs have a resolution of 4096 pixels × 3775 pixels, the size of all attached tif-files exceeds the upload limit of “Data in Brief” (500 MB). Therefore, in the attached zip-file, we only provide one BSE micrograph per grain size. However, to make all BSE images available, the complete set of BSE micrographs can be either downloaded from https://ruhr-uni-bochum.sciebo.de/s/dkr1YdHihA4rTJL or be sent on request by email. The BSE-images were used in combination with the lineal intercept method to determine the grain- and crystallite-size distributions, see Fig. 1, Fig. 2, Table 1, Table 2, and pdf-reports in the supplementary zip-file. Fig. 1a shows two Histograms that compare the grain size distributions of specimens having the smallest (*d* = 17 μm, red data) and the biggest (*d* = 216 μm, purple data) average grain sizes. Fig. 1b shows a probability plot of the cumulative frequency vs. the logarithm of grain diameter class for the seven specimens with different recrystallized microstructures, that were used for compression tests. Note that a numerical linearization of the Gaussian distribution function was used on the scale of the *y*-axis in Fig. 1b. The BSE micrographs were also used to measure the average grain (*d*) and crystallite (*c*) sizes, the number of annealing twin boundaries per grain (*n*) and the distribution of the annealing twin thickness (*t*), which are reported in Table 1, Table 2, Table 3, Table 4, respectively. All values are given with their respective uncertainties.

**Table 1:** Grain size distribution and mean grain size (*d*) with uncertainty (Δ*d*), after heat treatments at different temperatures and times. These data were obtained from BSE micrographs. The parameter (*d*) only accounts for the intersections of the test lines with grain-boundaries.

Cluster	Absolute frequency								
	1073K (45min)	1073K (60min)	1073K (120min)	1173K (30min)	1173K (60min)	1273K (60min)	1373K (30min)	1373K (60min)	1473K (60min)
0–2 μm	5	11	2	1	3	3	–	–	–
2–3 μm	12	13	4	2	3	2	–	–	–
3–4 μm	17	27	4	2	8	1	–	–	–
4–5 μm	25	46	13	17	15	2	1	–	–
5–7 μm	71	105	39	25	28	7	–	1	–
7–10 μm	149	189	59	47	56	14	2	0	–
10–13 μm	134	160	106	56	55	21	3	1	–
13–19 μm	213	276	194	172	152	52	4	3	2
19–27 μm	161	265	199	259	195	56	8	4	–
27–38 μm	90	166	141	324	228	86	15	5	–
38–75 μm	35	104	84	335	338	259	53	22	4
75–107 μm	2	2	3	11	48	136	52	17	5
107–151 μm	–	–	–	1	4	93	50	38	17
151–214 μm	–	–	2	–	–	32	31	35	27
214–302 μm	–	–	1	–	–	5	17	30	18
302–427 μm	–	–	–	–	–	–	6	8	14
427–600 μm	–	–	–	–	–	–	1	8	2
600 μm +	–	–	–	–	–	–	–	–	1
***d* (μm)**	**17**	**19**	**22**	**30**	**33**	**66**	**112**	**167**	**216**
**Δ*d* (μm)**	**1**	**1**	**2**	**1**	**2**	**2**	**5**	**7**	**10**

**Table 2:** Crystallite size distribution and mean grain size (*c*) with uncertainty (Δ*c*), after heat treatments at different temperatures and times. These data were obtained from BSE micrographs. The parameter (*c*) is determined by counting intersections with both grain and annealing twin boundaries.

Cluster	Absolute frequency								
	1073K (45min)	1073K (60min)	1073K (120min)	1173K (30min)	1173K (60min)	1273K (60min)	1373K (30min)	1373K (60min)	1473K (60min)
0–2 μm	32	39	31	43	7	1	–	–	–
2–3 μm	52	54	79	70	27	3	–	–	–
3–4 μm	61	76	73	71	31	11	–	–	1
4–5 μm	70	68	94	103	43	18	2	1	3
5–7 μm	142	141	164	168	91	33	1	5	1
7–10 μm	223	227	209	270	124	48	7	6	5
10–13 μm	147	189	182	225	123	43	7	6	2
13–19 μm	244	312	253	363	256	102	14	10	4
19–27 μm	135	234	165	387	250	124	19	11	10
27–38 μm	63	128	98	277	243	131	23	16	10
38–75 μm	20	101	42	154	262	309	94	68	31
75–107 μm	1	4	3	3	32	123	66	45	19
107–151 μm	1	–	1	–	6	67	43	40	29
151–214 μm	–	–	–	–	–	21	26	34	23
214–302 μm	–	–	–	–	–	2	11	17	14
302–427 μm	–	–	–	–	–	1	3	11	8
427–600 μm	–	–	–	–	–	–	1	1	1
600 μm +	–	–	–	–	–	–	–	–	1
***c* (μm)**	**13**	**16**	**14**	**18**	**25**	**49**	**86**	**106**	**120**
**Δ*c* (μm)**	**1**	**1**	**1**	**1**	**1**	**4**	**6**	**6**	**8**

**Table 3:** Average number of annealing twin boundaries per grain (*n*).

	1073K (45min)	1073K (60min)	1073K (120min)	1173K (30min)	1173K (60min)	1273K (60min)	1373K (30min)	1373K (60min)	1473K (60min)
*n* (−)	0.3	0.1	0.6	0.4	0.3	0.3	0.3	0.3	0.8
Δ*n* (−)	0.04	0.01	0.06	0.01	0.01	0.03	0.03	0.02	0.01

**Table 4:** Twin thickness distribution and average twin thicknesses (*t*) with uncertainty (Δ*t*), after heat treatments at different temperatures and times obtained on BSE micrographs.

Cluster	Absolute frequency								
	1073K (45min)	1073K (60min)	1073K (120min)	1173K (30min)	1173K (60min)	1273K (60min)	1373K (30min)	1373K (60min)	1473K (60min)
0–2 μm	18	17	14	6	2	2	–	–	–
2–3 μm	10	22	34	32	8	3	–	–	–
3–4 μm	13	15	23	24	6	1	–	–	–
4–5 μm	11	13	20	26	12	5	–	–	–
5–7 μm	15	20	35	51	11	12	1	1	–
7–10 μm	10	15	26	82	15	10	3	4	2
10–13 μm	4	8	21	40	8	6	1	1	–
13–19 μm	3	10	11	45	26	9	4	8	2
19–27 μm	–	5	3	29	13	18	5	6	–
27–38 μm	–	–	–	5	10	11	6	7	3
38–75 μm	–	–	–	–	11	9	5	15	2
75–107 μm	–	–	–	–	–	2	1	5	–
107–151 μm	–	–	–	–	–	–	–	6	1
151–214 μm	–	–	–	–	–	–	–	3	–
214–302 μm	–	–	–	–	–	–	–	–	1
302–427 μm	–	–	–	–	–	–	–	–	–
427–600 μm	–	–	–	–	–	–	–	–	–
600 μm +	–	–	–	–	–	–	–	–	–
***t* (μm)**	**4.5**	**7.0**	**7.0**	**9.6**	**15**	**21**	**30**	**55**	**68**
**Δ*t* (μm)**	**0.7**	**0.6**	**0.7**	**0.9**	**1**	**5**	**6**	**11**	**14**

Additionally to the Heyn lineal intercept method performed on BSE micrographs, we also used another method to determine mean grain- and crystallite size distributions, which is based on electron backscatter diffraction (EBSD), see Table 5, Table 6. Table 7 compares the mean grain sizes and corresponding standard deviations obtained with these two different methods for all recrystallized microstructures. Also shown in Table 7 are the Taylor factors (*M*) which were determined by EBSD. Fig. 2 compares the grain size distributions of the specimen showing the smallest grain size (*d* = 17 μm, 1073 K for 45 min) obtained by the two different methods. The blue histogram and the fitted Gaussian curve represent the data obtained by EBSD whereas those in red color were obtained using the Heyn lineal intercept method on BSE micrographs.

**Table 5:** Grain size distribution and mean grain size (*d*_EBSD_) with uncertainty (Δ*d*_EBSD_), after heat treatments at different temperatures and times. These data were obtained by EBSD.

Cluster	Absolute frequency								
	1073K (45min)	1073K (60min)	1073K (120min)	1173K (30min)	1173K (60min)	1273K (60min)	1373K (30min)	1373K (60min)	1473K (60min)
0–2 μm	2	5	–	–	–	–	–	–	–
2–3 μm	2	10	–	–	–	–	–	–	–
3–4 μm	6	11	10	5	2	–	–	–	–
4–5 μm	4	9	7	3	4	–	–	–	–
5–7 μm	7	21	13	6	4	–	–	–	–
7–10 μm	23	43	21	6	7	4	–	–	–
10–13 μm	8	38	26	2	7	5	–	–	–
13–19 μm	20	48	41	30	13	2	2	–	–
19–27 μm	10	44	25	18	22	14	–	2	–
27–38 μm	13	24	13	14	24	20	1	–	–
38–75 μm	6	12	5	19	42	76	10	7	–
75–107 μm	–	–	–	–	17	40	9	4	2
107–151 μm	–	–	–	–	3	24	14	12	4
151–214 μm	–	–	–	–	–	10	9	12	12
214–302 μm	–	–	–	–	–	–	4	12	18
302–427 μm	–	–	–	–	–	–	1	3	10
427–600 μm	–	–	–	–	–	–	–	–	4
600 μm +	–	–	–	–	–	–	–	–	3
***d***_**EBSD**_**(μm)**	**17**	**15**	**15**	**23**	**38**	**71**	**123**	**171**	**213**
**Δ*d***_**EBSD**_**(μm)**	**1**	**2**	**1**	**2**	**3**	**5**	**7**	**9**	**12**

**Table 6:** Crystallite size distribution and mean crystallite size (*c*_EBSD_) with uncertainty (Δ*c*_EBSD_), after heat treatments at different temperatures and times. These data were obtained by EBSD.

Cluster	Absolute frequency								
	1073K (45min)	1073K (60min)	1073K (120min)	1173K (30min)	1173K (60min)	1273K (60min)	1373K (30min)	1373K (60min)	1473K (60min)
0–2 μm	12	34	–	–	–	–	–	–	–
2–3 μm	49	84	16	–	–	–	–	–	–
3–4 μm	27	88	54	20	12	–	–	–	–
4–5 μm	49	52	44	19	20	–	–	–	–
5–7 μm	41	116	46	35	51	–	–	–	–
7–10 μm	57	137	88	36	43	57	–	–	–
10–13 μm	44	114	59	45	35	23	7	–	–
13–19 μm	47	98	74	60	107	83	11	–	–
19–27 μm	27	62	14	26	81	75	11	8	3
27–38 μm	9	7	10	22	55	76	16	15	7
38–75 μm	2	1	–	9	67	161	54	51	30
75–107 μm	–	–	–	–	6	48	29	38	17
107–151 μm	–	–	–	–	–	17	21	23	15
151–214 μm	–	–	–	–	–	6	6	14	45
214–302 μm	–	–	–	–	–	–	4	8	17
302–427 μm	–	–	–	–	–	–	–	2	8
427–600 μm	–	–	–	–	–	–	–	–	2
600 μm +	–	–	–	–	–	–	–	–	–
***c***_**EBSD**_**(μm)**	**10**	**9**	**10**	**14**	**22**	**40**	**69**	**96**	**152**
**Δ*c***_**EBSD**_**(μm)**	**1**	**2**	**1**	**2**	**3**	**5**	**7**	**9**	**12**

**Table 7:** Comparison of the mean grain size (excluding twin boundaries) obtained using the linear intercept method (*d*_*LIM*_) with that determined by EBSD (*d*_*EBSD*_). Additionally given are the corresponding Taylor factors (*M*).

*d*_LIM_ (μm)	*d*_EBSD_ (μm)	*M*
17 ± 1	17 ± 1	3.06
19 ± 1	15 ± 2	3.09
22 ± 2	15 ± 1	3.06
30 ± 1	23 ± 2	3.06
33 ± 2	38 ± 3	3.03
66 ± 2	71 ± 5	3.11
112 ± 5	123 ± 7	3.04
167 ± 7	171 ± 9	3.05
216 ± 10	213 ± 12	3.14

For seven of the nine grain sizes, compression tests were conducted. The Excel-sheets containing the corresponding stress-strain data can be found in the zip-file under the “Compression_Tests”-folder. This folder is divided into eight subfolders corresponding to eight testing temperatures. The Excel-sheets in these folders are named using the three following characteristics: alloy composition, recrystallization heat treatment (temperature and time), and compression test temperature. The Excel-sheet for a compression test conducted at 873 K, where the sample was recrystallized at 1073 K for 45 min is, therefore, labeled as: “MnFeNi_1073 K_45min_873 K”. From these stress-strain datasets, the yield stresses at 0.2% plastic deformation (*σ*_0.2%_) determined at different temperatures for various grain and crystallite sizes are given in Table 8. These data allowed us to plot the yield stress as a function of the square root of the average grain/crystallite size. From these Hall-Petch plots, the intrinsic lattice strength (*σ*_0_) and the Hall-Petch slope (*k*_y_) were determined following the procedures reported in Ref. [1]. These values were then respectively normalized by *G* and *Gb*^1/2^, where *G* is the temperature-dependent shear modulus and *b* is the Burgers vector, as shown in Ref. [10]. Both parameters were taken from Ref. [7]. The normalized data (*σ*_0_/*G* and *k*_y_/(*Gb*^1/2^)) are listed in Table 9. Using the temperature dependence of the yield stress obtained for the biggest grain/crystallite size (see Ref. [1]), the intrinsic lattice strength and the Hall-Petch slope were calculated (interpolated) for temperatures of 173 K, 223 K, 373 K, and 473 K using Eqs. (1) and (2) of Ref. [1]. These interpolated values are marked with an asterisk in Table 9. For further details on the experimental methods and calculations, the reader can refer to the related research article [1].

**Table 8:** Yield stresses *σ*_*0.2*%_ for nine grain (*d*) and crystallite (*c*) sizes obtained at eight different temperatures.

*d* (μm)	*c* (μm)	σ_0.2%_*(MPa*)							
		*77 K*	*173K*	*223K*	*293 K*	*373K*	*473K*	*673 K*	873 K
*17 ± 1*	*13 ± 1*	388 ± 8	–	–	263 ± 5	–	–	184 ± 4	192 ± 4
*19 ± 1*	*16 ± 1*	384 ± 8	–	–	252 ± 5	–	–	173 ± 4	179 ± 4
*22 ± 2*	*14 ± 1*	360 ± 7	–	–	239 ± 5	–	–	177 ± 4	175 ± 4
*33 ± 2*	*25 ± 1*	341 ± 7	–	–	206 ± 4	–	–	128 ± 3	148 ± 3
*66 ± 2*	*49 ± 4*	315 ± 6	–	–	175 ± 4	–	–	91 ± 2	111 ± 2
*112 ± 5*	*86 ± 6*	278 ± 6	–	–	155 ± 3	–	–	103 ± 2	95 ± 2
*216 ± 10*	*120 ± 8*	283 ± 6	182 ± 10	165 ± 10	146 ± 3	130 ± 9	104 ± 7	88 ± 2	96 ± 2

**Table 9:** Hall-Petch parameters (*σ*_*0*_ and *k*_*y*_) normalized by the shear modulus *G* and *Gb*^1/2^, respectively, for eight different temperatures.

T (K)	*(σ*_*0*_*/G)* × *1000* (MPa)		*k*_*y*_*/Gb*^*1/2*^ (−)		*G* (GPa)
	*d*	*c*	*d*	*c*	Ref. [7]
*77*	3.00 ± 0.01	2.97 ± 0.01	0.49 ± 0.03	0.43 ± 0.03	81.9
*173*^a^	1.70 ± 0.08	1.59 ± 0.08	0.51 ± 0.03	0.46 ± 0.03	79.9
*223*^a^	1.54 ± 0.06	1.43 ± 0.06	0.52 ± 0.02	0.47 ± 0.02	78.5
*293*	1.27 ± 0.05	1.20 ± 0.05	0.54 ± 0.02	0.49 ± 0.02	76.1
*373*^a^	1.06 ± 0.05	0.94 ± 0.05	0.53 ± 0.02	0.48 ± 0.02	73.3
*473*^a^	0.89 ± 0.04	0.76 ± 0.04	0.55 ± 0.02	0.49 ± 0.02	69.6
*673*	0.71 ± 0.05	0.63 ± 0.05	0.56 ± 0.02	0.51 ± 0.02	62.0
*873*	0.90 ± 0.06	0.81 ± 0.06	0.65 ± 0.02	0.59 ± 0.02	54.3

a calculated data.

## 2 Experimental design, materials, and methods

BSE micrographs were recorded in an SEM of type Quanta FEI 650 ESEM operating at a working distance of ∼10 mm. Acceleration voltages between 15 kV (small grains) and 20 kV (large grains) were chosen to optimize the BSE contrast. Four BSE images spaced 1 mm apart were collected for each grain size, except for the three coarsest microstructures. In this latter case, nine images were collected and assembled, covering an area representative of the whole cross-section of a compression specimen. These micrographs were then used to determine the mean grain (*d*) and mean crystallite (*c*) sizes and their distributions using the Heyn lineal intercept method with four horizontal and four vertical lines. Each line intersected ∼50 grains resulting in 300–500 intercepts per micrograph, similar to the procedure reported in Ref. [2]. The same procedure was used to determine the size distribution of the annealing twins, which is reported in Table 4 including the mean values (*t*) and corresponding uncertainties (Δ*t*). Using the data for *d* and *c* and the equation *n* = (*d*/*c* – 1), the average number of annealing twin boundaries per grain (*n*) was calculated, see Table 3.

Grain orientation maps were determined by electron backscatter diffraction (EBSD) in the above-mentioned SEM equipped with a Hikari XP camera (EDAX, AMETEK). From these orientation maps, grain- and crystallite size distributions (*d*_EBSD_, *c*_EBSD_, see Table 5, Table 6, respectively) and Taylor-factors (*M*, see Table 7) were determined. Evaluation of the data was done using the TSL OIM Analysis (version 6.2.0) software. Fig. 2 and Table 7 compare the results of the two previously mentioned methods, namely the Heyn lineal intercept method performed on BSE micrographs (*d*_LIM_, previous paragraph) and EBSD (*d*_EBSD_). Please note that a comparison of the two methods for the crystallite size would not be appropriate. Indeed, as grain/crystallite sizes obtained by EBSD are calculated using *d* = (*A* π/4)^1/2^ or *c* = (*A* π/4)^1/2^, where *A* is the cross-sectional area of the grain/crystallite and since annealing twins are not equiaxed, but exhibit an elongated geometry, the equation *c* = (*A* π/4)^1/2^ should not be used to compute a mean crystalitte size according to the standard test method ASTM E−112 [11].

Compression tests were conducted in a Zwick Roell XForce Z100 machine at temperatures ranging from 77 K to 873 K and at a nominal strain rate of 10^−3^ s^−1^. The compression specimens were deformed up to plastic strains ranging between 16% and 22%.
