# Supplementary material for: Data compilation on the effect of grain size, temperature, and texture on the strength of a single-phase FCC MnFeNi medium-entropy alloy
Source: Data Brief. 2019 Nov 15;28:104807. doi: 10.1016/j.dib.2019.104807 (PMC6909151; doi:10.1016/j.dib.2019.104807)
Supplement: Multimedia component 1 [file mmc1.zip › MnFeNi_1073K_120min/MnFeNi_1073K_120min_c=14μm.pdf]

Analysebericht

Jun 13, 2018 12:04:40 PM

powered by imagic.ch

1. cumulative Result 1

Number of images	4
Grain size (ASTM)	9.1
Grain size (G643)	9.1
Grain stretching	99.2 %
Mean chord length	13.5 µm

2. Single Result 1 (MnFeNi Semesterprojekt_MnFeNi_homogenized_8.1mmSW_800°C_120min_00098)

Mean chord length	13.8 µm
Grain size (ASTM)	9.1
Grain size (G643)	9
Grain stretching	87.1 %

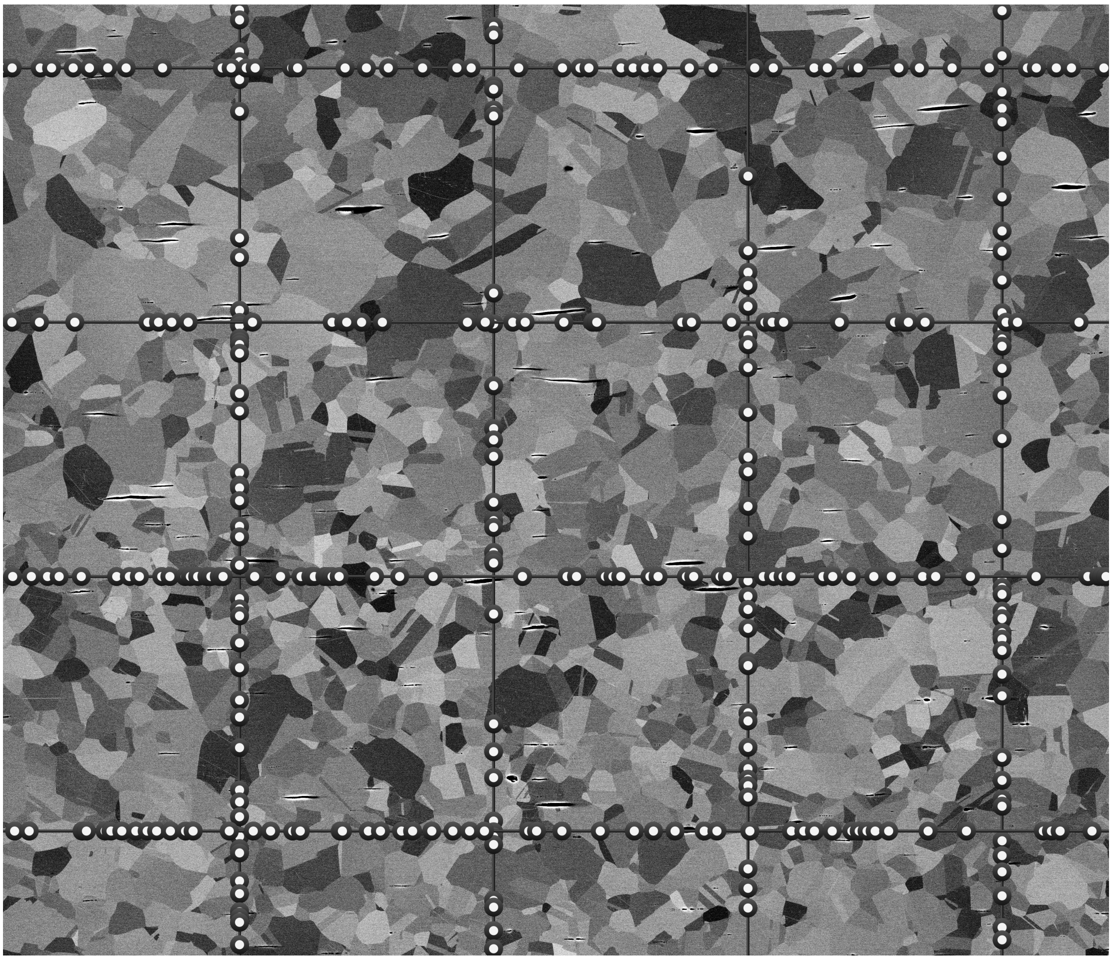

2.1. Statistical Analysis

Statistical Data	Length
Object Count	341
Minimum	0.6 µm
Maximum	101.5 µm
Average	13.8 µm
Standard deviation	12.5 µm
Skewness	0.0
Standard deviation (n-1)	12.5 µm
Variance	156.6 µm²
Variance (n-1)	157.1 µm²
Sum	4'714.0 µm
Sum of squares	118'573.3 µm²

Statistical Data	Length
Sum of cubes	5'092'228.7 µm³

2.1.1. Chord Lenght Distribution

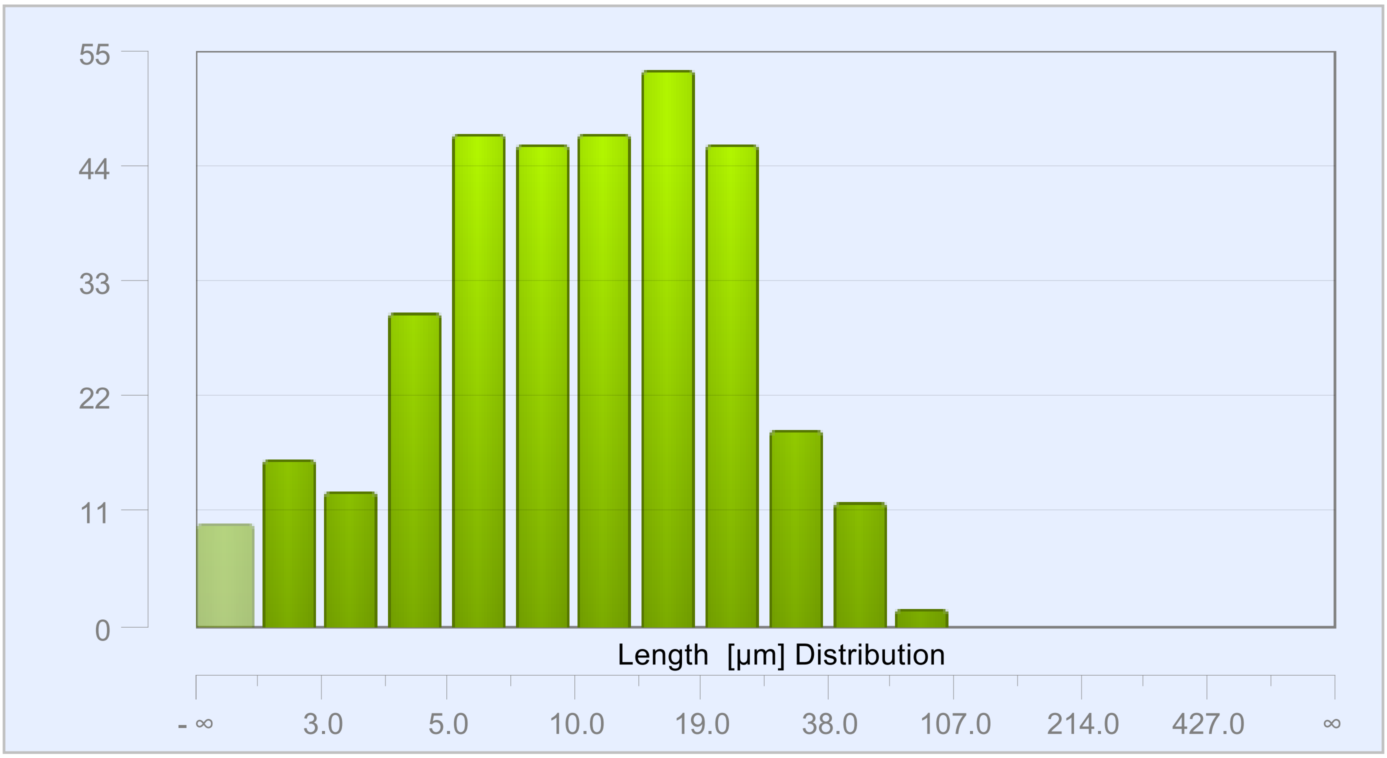

Start	End	Absolute Frequency	Absolute Frequency (accumulated)	Relative Frequency [%]	Relative Frequency (accumulated) [%]
	2.0 µm	10	10	3	3
2.0 µm	3.0 µm	16	26	5	8
3.0 µm	4.0 µm	13	39	4	11
4.0 µm	5.0 µm	30	69	9	20
5.0 µm	7.0 µm	47	116	14	34
7.0 µm	10.0 µm	46	162	13	48
10.0 µm	13.0 µm	47	209	14	61
13.0 µm	19.0 µm	53	262	16	77
19.0 µm	27.0 µm	46	308	13	90
27.0 µm	38.0 µm	19	327	6	96
38.0 µm	75.0 µm	12	339	4	99
75.0 µm	107.0 µm	2	341	1	100
107.0 µm	151.0 µm	0	341	0	100
151.0 µm	214.0 µm	0	341	0	100
214.0 µm	302.0 µm	0	341	0	100
302.0 µm	427.0 µm	0	341	0	100
427.0 µm	600.0 µm	0	341	0	100
600.0 µm		0	341	0	100

3. Single Result 2 (MnFeNi Semesterprojekt_MnFeNi_homogenized_8.1mmSW_800°C_120min_00099)

Mean chord length	13.3 µm
Grain size (ASTM)	9.2
Grain size (G643)	9.1
Grain stretching	92.1 %

3.1. Statistical Analysis

Statistical Data	Length
Object Count	355
Minimum	1.6 µm
Maximum	63.6 µm
Average	13.3 µm
Standard deviation	10.5 µm
Skewness	0.0
Standard deviation (n-1)	10.5 µm
Variance	110.2 µm²
Variance (n-1)	110.6 µm²
Sum	4'715.0 µm
Sum of squares	101'761.4 µm²
Sum of cubes	3'062'582.2 µm³

3.1.1. Chord Lenght Distribution

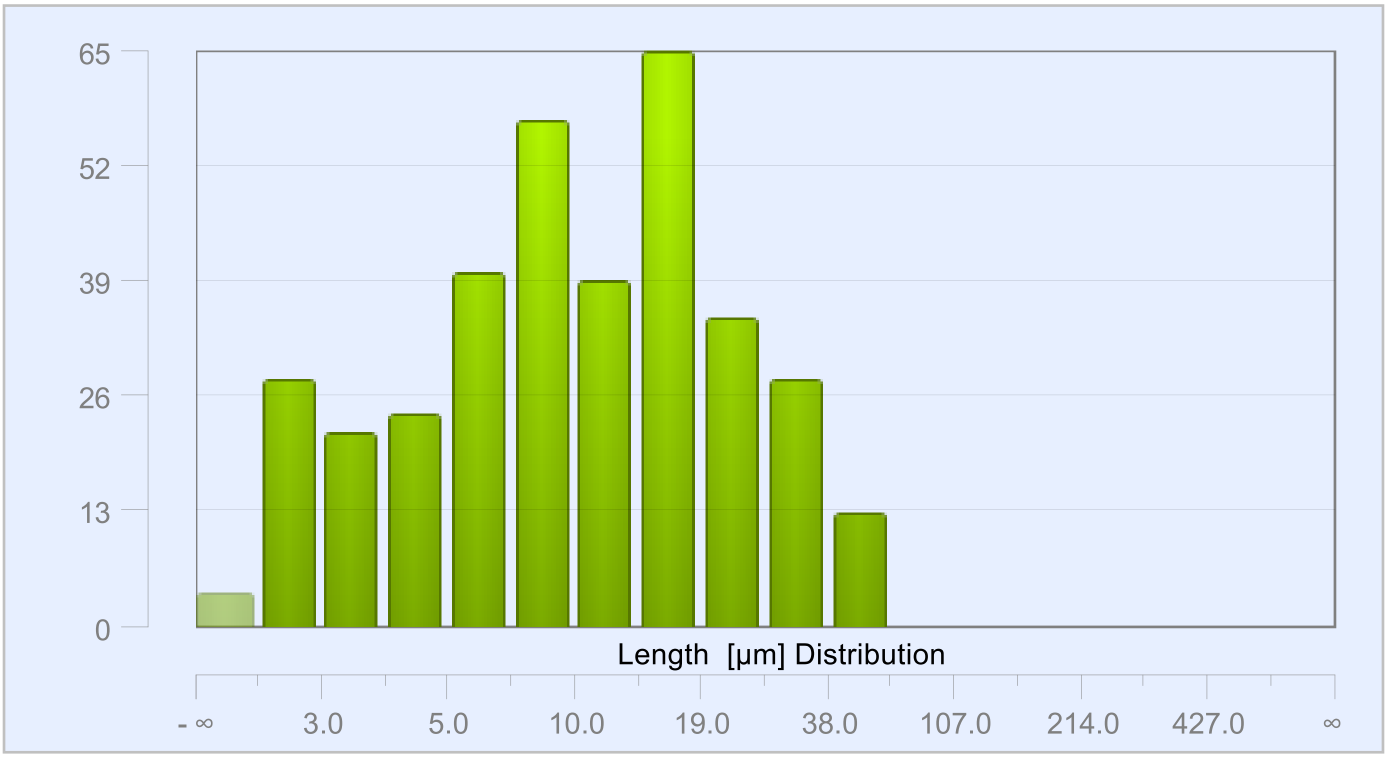

Start	End	Absolute Frequency	Absolute Frequency (accumulated)	Relative Frequency [%]	Relative Frequency (accumulated) [%]
	2.0 µm	4	4	1	1
2.0 µm	3.0 µm	28	32	8	9
3.0 µm	4.0 µm	22	54	6	15
4.0 µm	5.0 µm	24	78	7	22
5.0 µm	7.0 µm	40	118	11	33
7.0 µm	10.0 µm	57	175	16	49
10.0 µm	13.0 µm	39	214	11	60
13.0 µm	19.0 µm	65	279	18	79
19.0 µm	27.0 µm	35	314	10	88
27.0 µm	38.0 µm	28	342	8	96
38.0 µm	75.0 µm	13	355	4	100
75.0 µm	107.0 µm	0	355	0	100

Start	End	Absolute Frequency	Absolute Frequency (accumulated)	Relative Frequency [%]	Relative Frequency (accumulated) [%]
107.0 µm	151.0 µm	0	355	0	100
151.0 µm	214.0 µm	0	355	0	100
214.0 µm	302.0 µm	0	355	0	100
302.0 µm	427.0 µm	0	355	0	100
427.0 µm	600.0 µm	0	355	0	100
600.0 µm		0	355	0	100

4. Single Result 3 (MnFeNi Semesterprojekt_MnFeNi_homogenized_8.1mmSW_800°C_120min_00100)

Mean chord length	14.3 µm
Grain size (ASTM)	9
Grain size (G643)	8.9
Grain stretching	97.4 %

4.1. Statistical Analysis

Statistical Data	Length
Object Count	330
Minimum	1.7 µm
Maximum	103.6 µm
Average	14.3 µm
Standard deviation	11.0 µm
Skewness	0.0
Standard deviation (n-1)	11.1 µm
Variance	121.9 µm²
Variance (n-1)	122.3 µm²
Sum	4'724.4 µm
Sum of squares	107'874.2 µm²
Sum of cubes	3'757'515.2 µm³

4.1.1. Chord Lenght Distribution

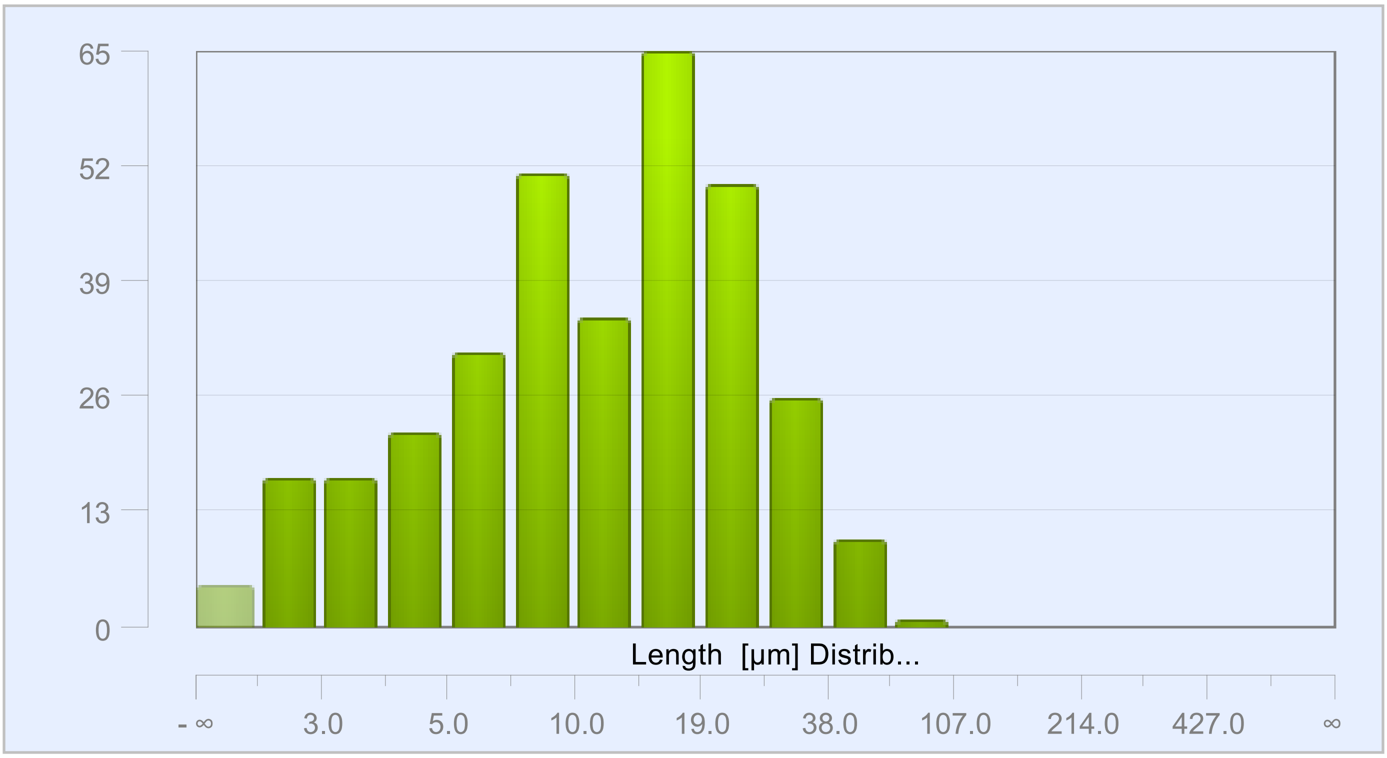

Start	End	Absolute Frequency	Absolute Frequency (accumulated)	Relative Frequency [%]	Relative Frequency (accumulated) [%]
	2.0 µm	5	5	2	2
2.0 µm	3.0 µm	17	22	5	7
3.0 µm	4.0 µm	17	39	5	12
4.0 µm	5.0 µm	22	61	7	18
5.0 µm	7.0 µm	31	92	9	28
7.0 µm	10.0 µm	51	143	15	43
10.0 µm	13.0 µm	35	178	11	54
13.0 µm	19.0 µm	65	243	20	74
19.0 µm	27.0 µm	50	293	15	89
27.0 µm	38.0 µm	26	319	8	97
38.0 µm	75.0 µm	10	329	3	100
75.0 µm	107.0 µm	1	330	0	100
107.0 µm	151.0 µm	0	330	0	100
151.0 µm	214.0 µm	0	330	0	100
214.0 µm	302.0 µm	0	330	0	100
302.0 µm	427.0 µm	0	330	0	100
427.0 µm	600.0 µm	0	330	0	100
600.0 µm		0	330	0	100

5. Single Result 4 (MnFeNi Semesterprojekt_MnFeNi_homogenized_8.1mmSW_800°C_120min_00101)

Mean chord length	12.8 µm
Grain size (ASTM)	9.3
Grain size (G643)	9.2
Grain stretching	94.7 %

5.1. Statistical Analysis

Statistical Data	Length
Object Count	368
Minimum	1.1 µm
Maximum	144.1 µm
Average	12.8 µm
Standard deviation	11.1 µm
Skewness	0.0
Standard deviation (n-1)	11.1 µm
Variance	123.1 µm²
Variance (n-1)	123.5 µm²
Sum	4'723.7 µm
Sum of squares	105'945.6 µm²
Sum of cubes	5'067'605.0 µm³

5.1.1. Chord Lenght Distribution

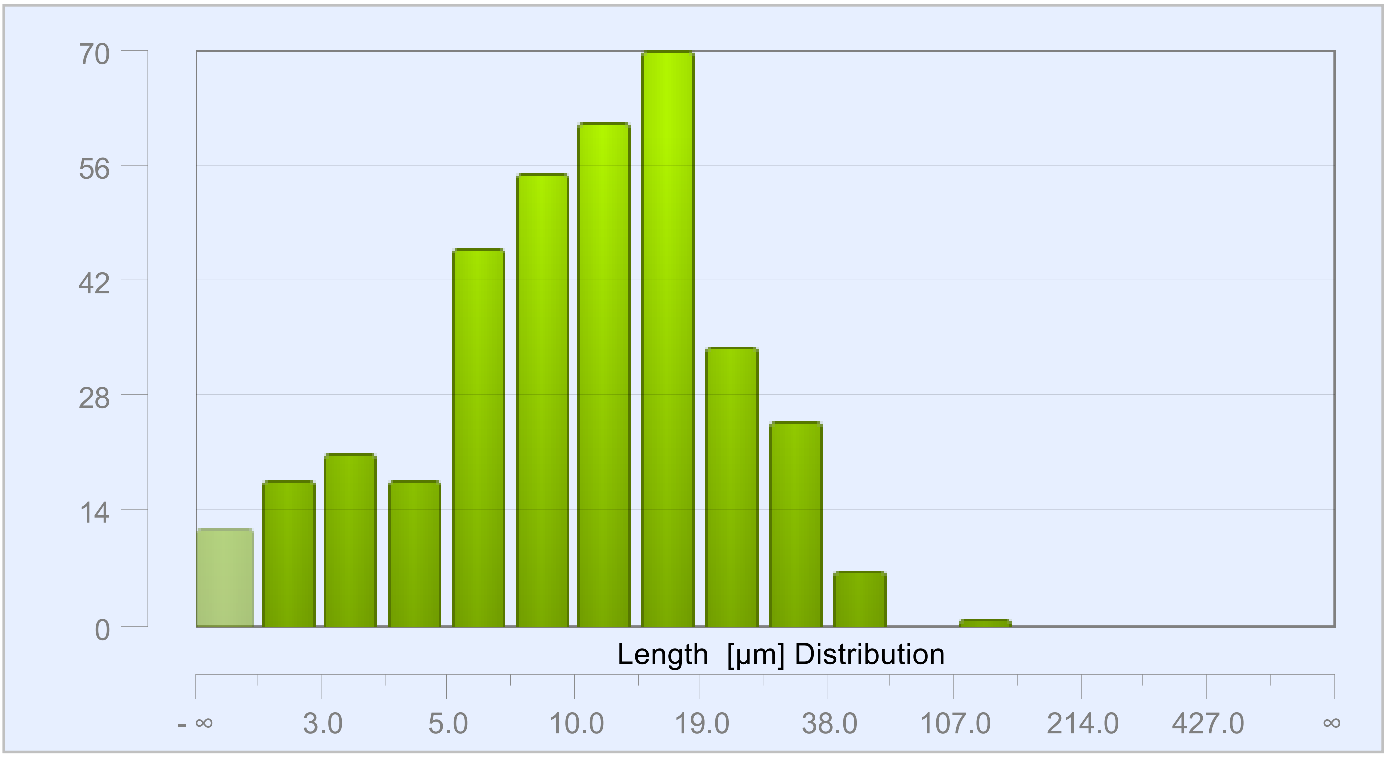

Start	End	Absolute Frequency	Absolute Frequency (accumulated)	Relative Frequency [%]	Relative Frequency (accumulated) [%]
	2.0 µm	12	12	3	3
2.0 µm	3.0 µm	18	30	5	8
3.0 µm	4.0 µm	21	51	6	14
4.0 µm	5.0 µm	18	69	5	19
5.0 µm	7.0 µm	46	115	12	31
7.0 µm	10.0 µm	55	170	15	46
10.0 µm	13.0 µm	61	231	17	63
13.0 µm	19.0 µm	70	301	19	82
19.0 µm	27.0 µm	34	335	9	91
27.0 µm	38.0 µm	25	360	7	98
38.0 µm	75.0 µm	7	367	2	100
75.0 µm	107.0 µm	0	367	0	100
107.0 µm	151.0 µm	1	368	0	100
151.0 µm	214.0 µm	0	368	0	100
214.0 µm	302.0 µm	0	368	0	100
302.0 µm	427.0 µm	0	368	0	100
427.0 µm	600.0 µm	0	368	0	100
600.0 µm		0	368	0	100
