# Supplementary material for: Data compilation on the effect of grain size, temperature, and texture on the strength of a single-phase FCC MnFeNi medium-entropy alloy
Source: Data Brief. 2019 Nov 15;28:104807. doi: 10.1016/j.dib.2019.104807 (PMC6909151; doi:10.1016/j.dib.2019.104807)
Supplement: Multimedia component 1 [file mmc1.zip › MnFeNi_1073K_120min/MnFeNi_1073K_120min_d=22μm.pdf]

Analysebericht

Jun 14, 2018 2:47:36 PM

powered by imagic.ch

1. cumulative Result 1

Number of images	4
Grain size (ASTM)	7.7
Grain size (G643)	7.7
Grain stretching	96 %
Mean chord length	22.2 µm

2. Single Result 1 (MnFeNi Semesterprojekt_MnFeNi_homogenized_8.1mmSW_800°C_120min_00098)

Mean chord length	24.1 µm
Grain size (ASTM)	7.5
Grain size (G643)	7.4
Grain stretching	84.3 %

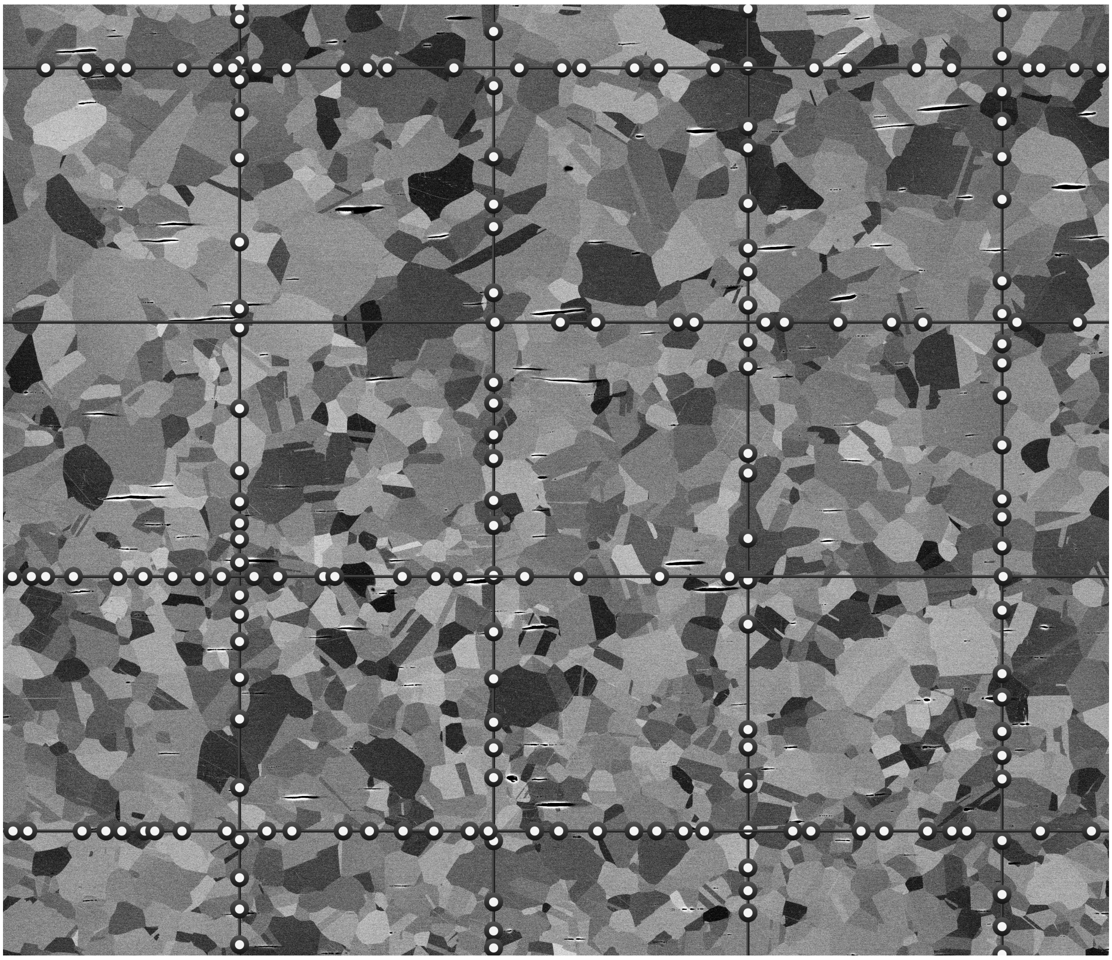

2.1. Statistical Analysis

Statistical Data	Length
Object Count	196
Minimum	0.5 µm
Maximum	281.9 µm
Average	24.1 µm
Standard deviation	24.1 µm
Skewness	0.0
Standard deviation (n-1)	24.2 µm
Variance	582.8 µm²
Variance (n-1)	585.8 µm²
Sum	4'714.0 µm
Sum of squares	227'611.0 µm²

Statistical Data	Length
Sum of cubes	30'576'560.8 µm³

2.1.1. Chord Lenght Distribution

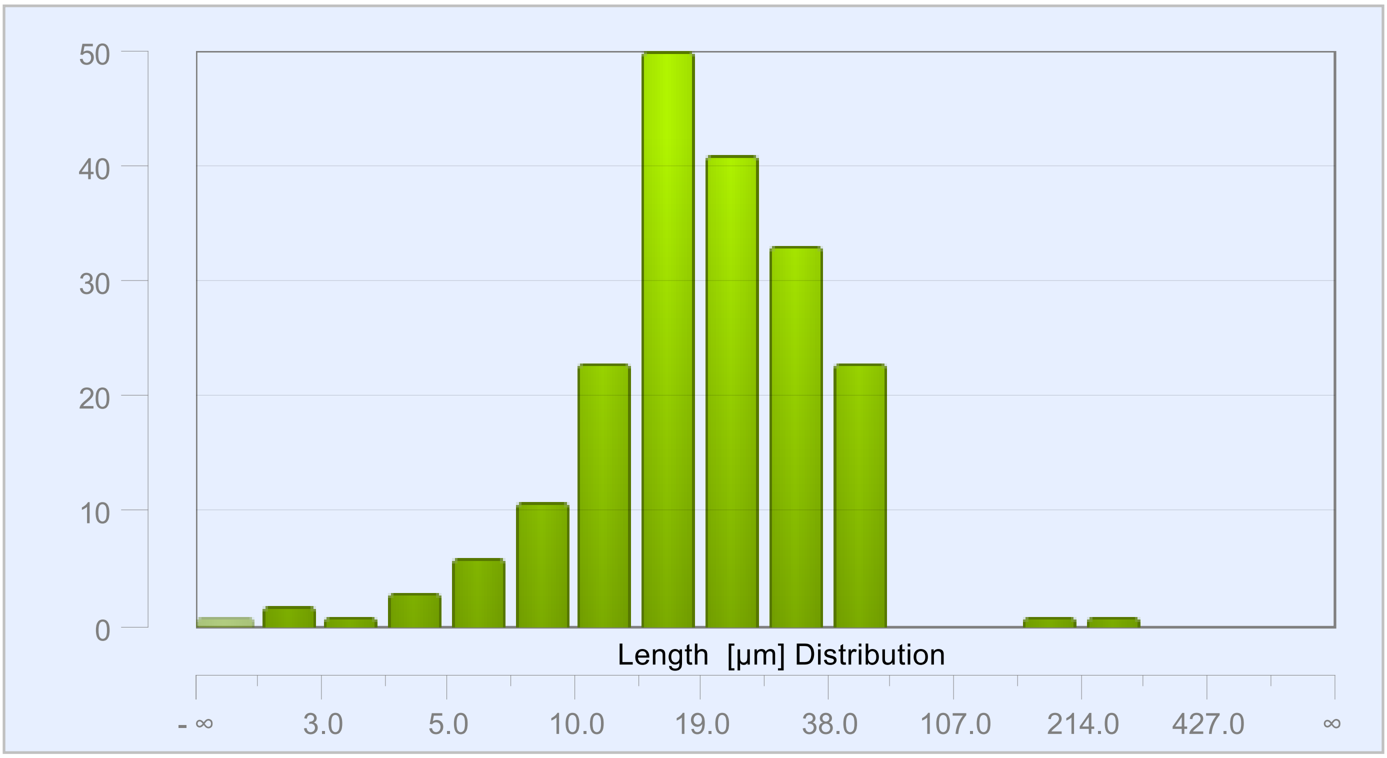

Start	End	Absolute Frequency	Absolute Frequency (accumulated)	Relative Frequency [%]	Relative Frequency (accumulated) [%]
	2.0 µm	1	1	1	1
2.0 µm	3.0 µm	2	3	1	2
3.0 µm	4.0 µm	1	4	1	2
4.0 µm	5.0 µm	3	7	2	4
5.0 µm	7.0 µm	6	13	3	7
7.0 µm	10.0 µm	11	24	6	12
10.0 µm	13.0 µm	23	47	12	24
13.0 µm	19.0 µm	50	97	26	49
19.0 µm	27.0 µm	41	138	21	70
27.0 µm	38.0 µm	33	171	17	87
38.0 µm	75.0 µm	23	194	12	99
75.0 µm	107.0 µm	0	194	0	99
107.0 µm	151.0 µm	0	194	0	99
151.0 µm	214.0 µm	1	195	1	99
214.0 µm	302.0 µm	1	196	1	100
302.0 µm	427.0 µm	0	196	0	100
427.0 µm	600.0 µm	0	196	0	100
600.0 µm		0	196	0	100

3. Single Result 2 (MnFeNi Semesterprojekt_MnFeNi_homogenized_8.1mmSW_800°C_120min_00099)

Mean chord length	21.7 µm
Grain size (ASTM)	7.8
Grain size (G643)	7.7
Grain stretching	96.2 %

3.1. Statistical Analysis

Statistical Data	Length
Object Count	217
Minimum	1.6 µm
Maximum	87.5 µm
Average	21.7 µm
Standard deviation	13.2 µm
Skewness	0.0
Standard deviation (n-1)	13.2 µm
Variance	173.0 µm²
Variance (n-1)	173.8 µm²
Sum	4'715.0 µm
Sum of squares	139'985.6 µm²
Sum of cubes	5'512'077.3 µm³

3.1.1. Chord Lenght Distribution

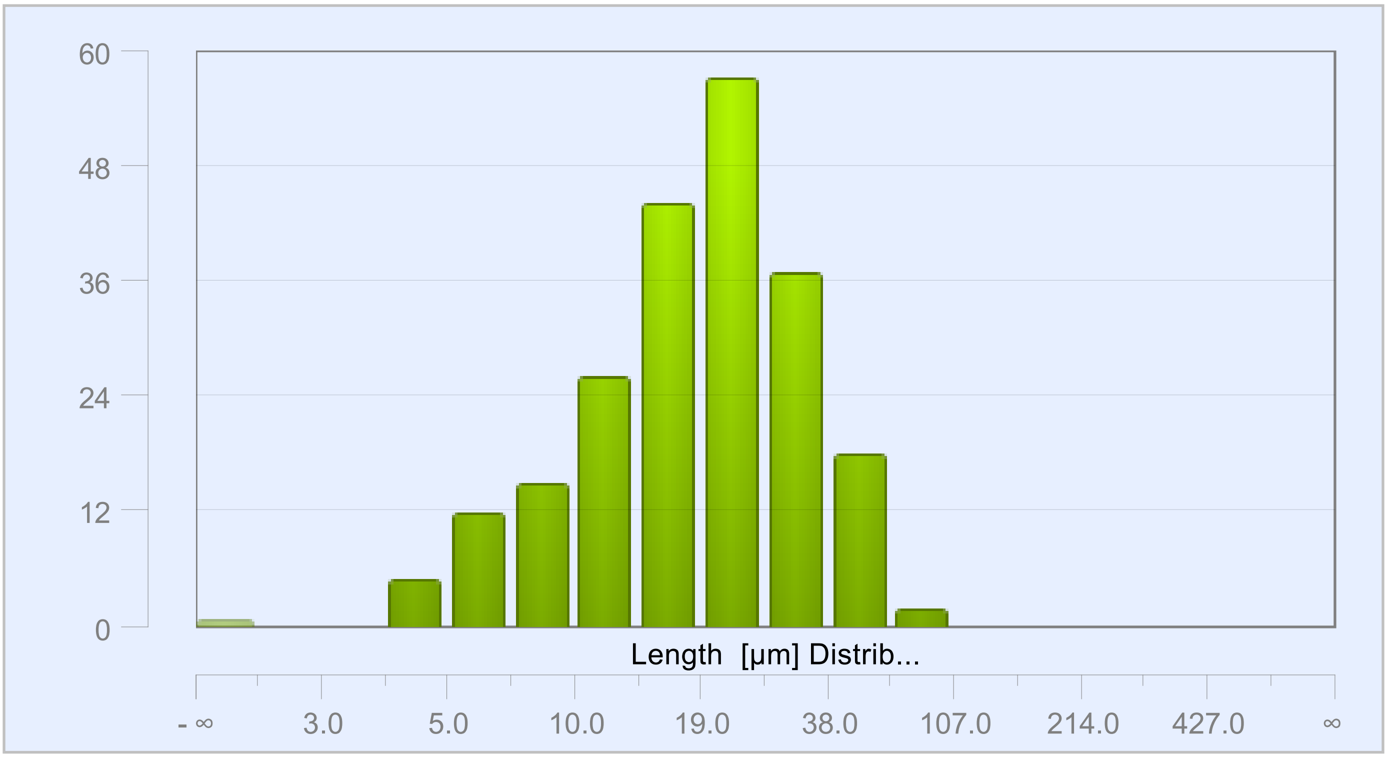

Start	End	Absolute Frequency	Absolute Frequency (accumulated)	Relative Frequency [%]	Relative Frequency (accumulated) [%]
	2.0 µm	1	1	0	0
2.0 µm	3.0 µm	0	1	0	0
3.0 µm	4.0 µm	0	1	0	0
4.0 µm	5.0 µm	5	6	2	3
5.0 µm	7.0 µm	12	18	6	8
7.0 µm	10.0 µm	15	33	7	15
10.0 µm	13.0 µm	26	59	12	27
13.0 µm	19.0 µm	44	103	20	47
19.0 µm	27.0 µm	57	160	26	74
27.0 µm	38.0 µm	37	197	17	91
38.0 µm	75.0 µm	18	215	8	99
75.0 µm	107.0 µm	2	217	1	100

Start	End	Absolute Frequency	Absolute Frequency (accumulated)	Relative Frequency [%]	Relative Frequency (accumulated) [%]
107.0 µm	151.0 µm	0	217	0	100
151.0 µm	214.0 µm	0	217	0	100
214.0 µm	302.0 µm	0	217	0	100
302.0 µm	427.0 µm	0	217	0	100
427.0 µm	600.0 µm	0	217	0	100
600.0 µm		0	217	0	100

4. Single Result 3 (MnFeNi Semesterprojekt_MnFeNi_homogenized_8.1mmSW_800°C_120min_00100)

Mean chord length	22.6 µm
Grain size (ASTM)	7.6
Grain size (G643)	7.6
Grain stretching	98.5 %

4.1. Statistical Analysis

Statistical Data	Length
Object Count	209
Minimum	2.9 µm
Maximum	102.4 µm
Average	22.6 µm
Standard deviation	13.4 µm
Skewness	0.0
Standard deviation (n-1)	13.4 µm
Variance	179.5 µm²
Variance (n-1)	180.4 µm²
Sum	4'724.4 µm
Sum of squares	144'320.1 µm²
Sum of cubes	5'735'478.8 µm³

4.1.1. Chord Lenght Distribution

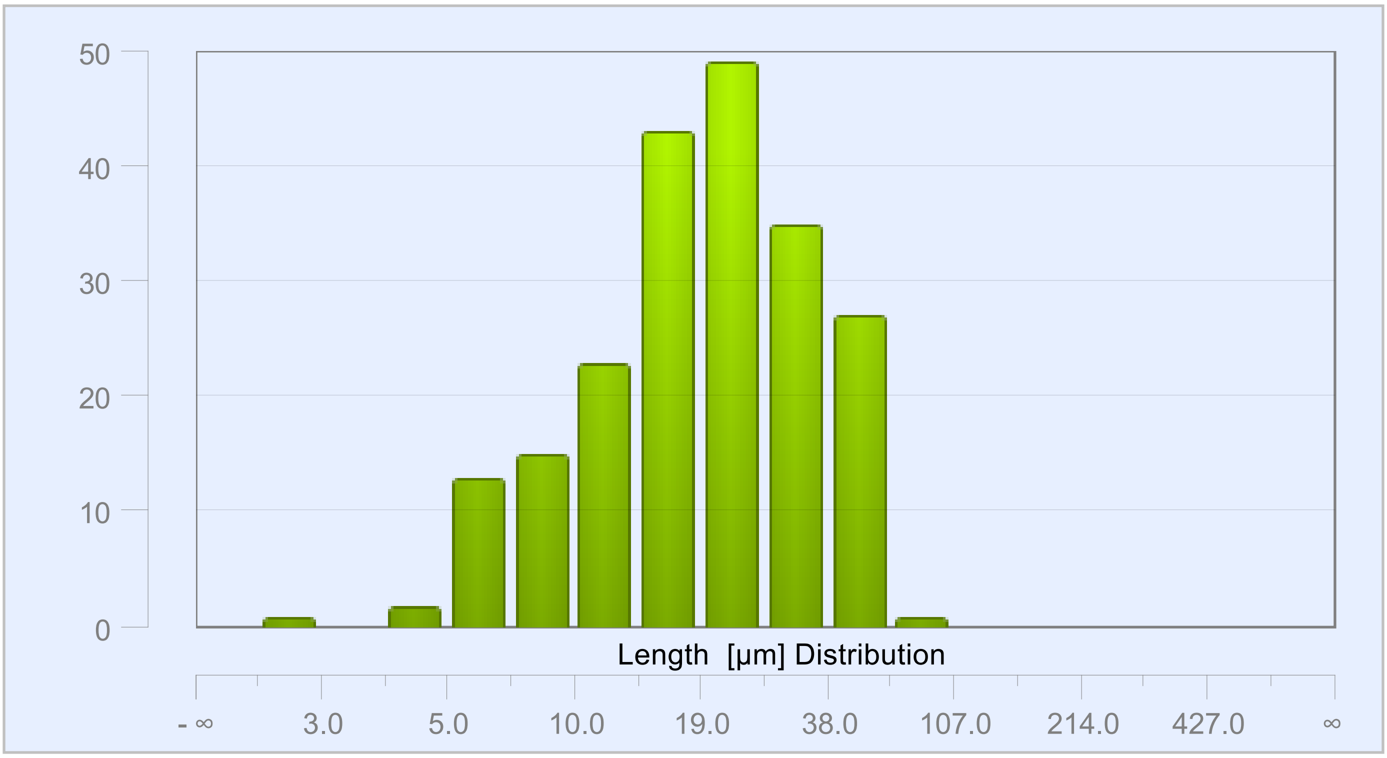

Start	End	Absolute Frequency	Absolute Frequency (accumulated)	Relative Frequency [%]	Relative Frequency (accumulated) [%]
	2.0 µm	0	0	0	0
2.0 µm	3.0 µm	1	1	0	0
3.0 µm	4.0 µm	0	1	0	0
4.0 µm	5.0 µm	2	3	1	1
5.0 µm	7.0 µm	13	16	6	8
7.0 µm	10.0 µm	15	31	7	15
10.0 µm	13.0 µm	23	54	11	26
13.0 µm	19.0 µm	43	97	21	46
19.0 µm	27.0 µm	49	146	23	70
27.0 µm	38.0 µm	35	181	17	87
38.0 µm	75.0 µm	27	208	13	100
75.0 µm	107.0 µm	1	209	0	100
107.0 µm	151.0 µm	0	209	0	100
151.0 µm	214.0 µm	0	209	0	100
214.0 µm	302.0 µm	0	209	0	100
302.0 µm	427.0 µm	0	209	0	100
427.0 µm	600.0 µm	0	209	0	100
600.0 µm		0	209	0	100

5. Single Result 4 (MnFeNi Semesterprojekt_MnFeNi_homogenized_8.1mmSW_800°C_120min_00101)

Mean chord length	20.6 µm
Grain size (ASTM)	7.9
Grain size (G643)	7.9
Grain stretching	94.6 %

5.1. Statistical Analysis

Statistical Data	Length
Object Count	229
Minimum	2.0 µm
Maximum	174.0 µm
Average	20.6 µm
Standard deviation	14.9 µm
Skewness	0.0
Standard deviation (n-1)	15.0 µm
Variance	223.2 µm²
Variance (n-1)	224.2 µm²
Sum	4'723.7 µm
Sum of squares	148'557.3 µm²
Sum of cubes	9'082'291.6 µm³

5.1.1. Chord Lenght Distribution

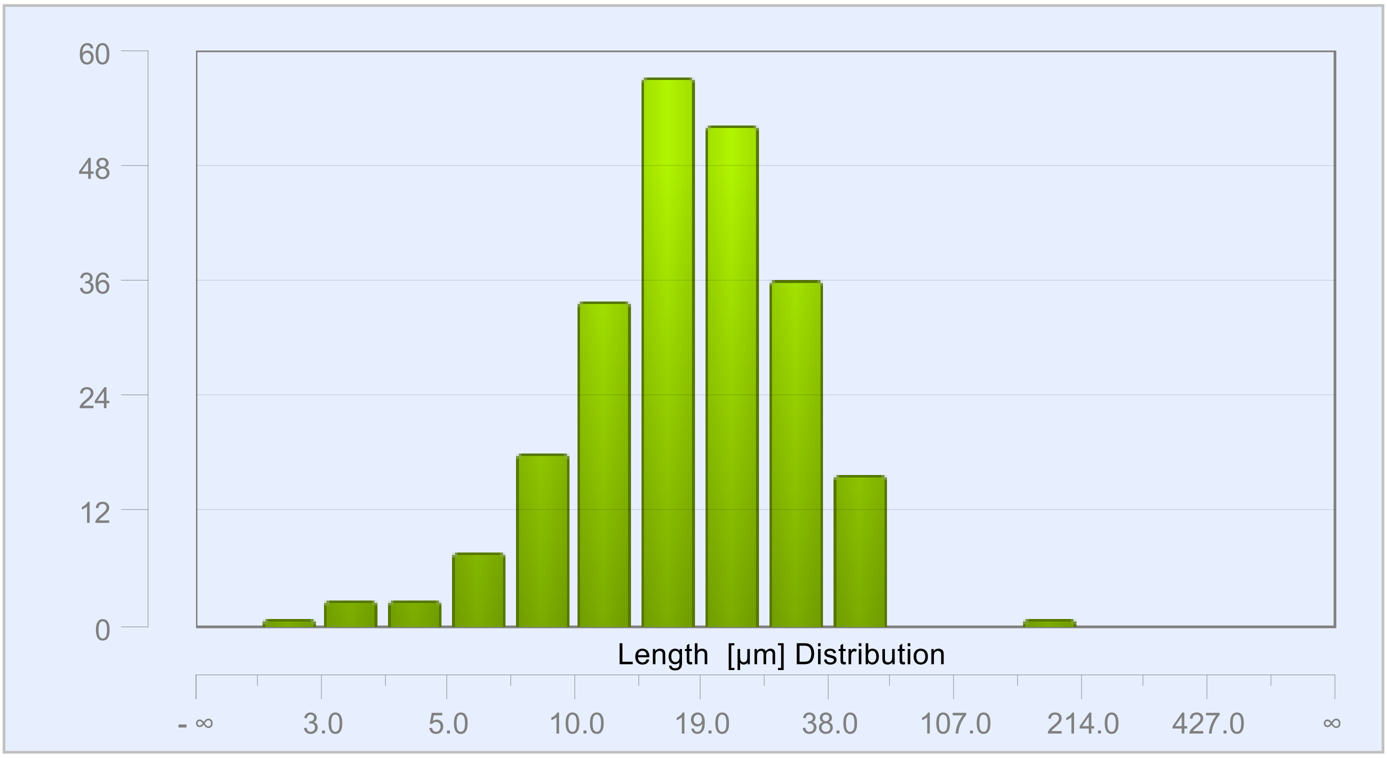

Start	End	Absolute Frequency	Absolute Frequency (accumulated)	Relative Frequency [%]	Relative Frequency (accumulated) [%]
	2.0 µm	0	0	0	0
2.0 µm	3.0 µm	1	1	0	0
3.0 µm	4.0 µm	3	4	1	2
4.0 µm	5.0 µm	3	7	1	3
5.0 µm	7.0 µm	8	15	3	7
7.0 µm	10.0 µm	18	33	8	14
10.0 µm	13.0 µm	34	67	15	29
13.0 µm	19.0 µm	57	124	25	54
19.0 µm	27.0 µm	52	176	23	77
27.0 µm	38.0 µm	36	212	16	93
38.0 µm	75.0 µm	16	228	7	100
75.0 µm	107.0 µm	0	228	0	100
107.0 µm	151.0 µm	0	228	0	100
151.0 µm	214.0 µm	1	229	0	100
214.0 µm	302.0 µm	0	229	0	100
302.0 µm	427.0 µm	0	229	0	100
427.0 µm	600.0 µm	0	229	0	100
600.0 µm		0	229	0	100
