# Supplementary material for: Data compilation on the effect of grain size, temperature, and texture on the strength of a single-phase FCC MnFeNi medium-entropy alloy
Source: Data Brief. 2019 Nov 15;28:104807. doi: 10.1016/j.dib.2019.104807 (PMC6909151; doi:10.1016/j.dib.2019.104807)
Supplement: Multimedia component 1 [file mmc1.zip › MnFeNi_1073K_45min/MnFeNi_1073K_45min_c=13μm.pdf]

Analysebericht

13.06.2018 15:45:07

powered by imagic.ch

1. cumulative Result 1

Anzahl Bilder	4
Korngröße (ASTM)	9,3
Korngröße (G643)	9,3
Kornstreckung	83,6 %
Mittlere Sehnenlänge	12,7 µm

2. Single Result 1 (MnFeNi Semesterprojekt_MnFeNi_homogenized_8.1mmSW_800°C_45min_00106)

Mittlere Sehnenlänge	12 µm
Korngröße (ASTM)	9,5
Korngröße (G643)	9,4
Kornstreckung	95,3 %

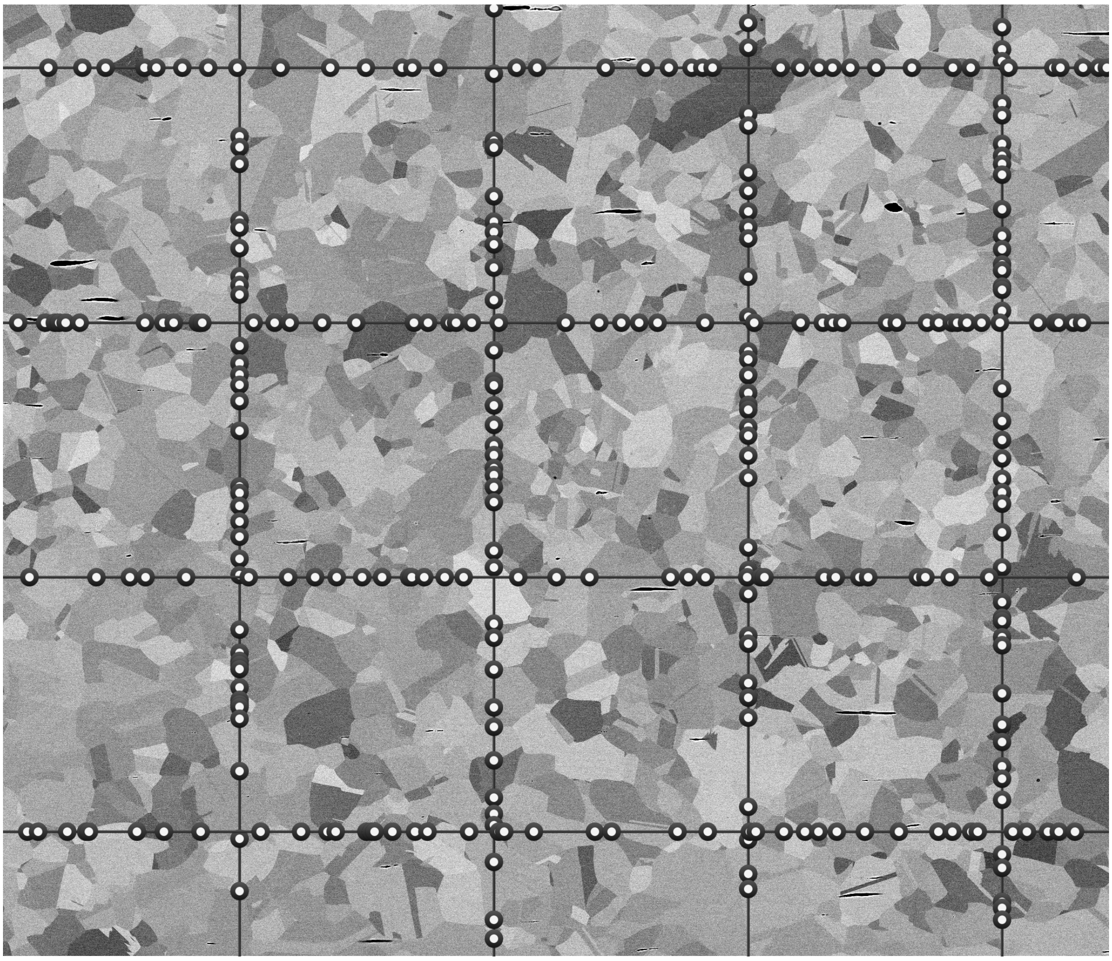

2.1. Statistische Analyse

Statistische Daten	Länge
Anzahl Objekte	314
Minimum	0,7 µm
Maximum	60,5 µm
Mittelwert	12,0 µm
Standardabweichung	8,6 µm
Schiefe	0,0
Standardabweichung (n-1)	8,6 µm
Varianz	74,0 µm²
Varianz (n-1)	74,3 µm²
Summe	3'779,7 µm

Statistische Daten	Länge
Quadratsumme	68'745,2 µm²
Kubiksumme	1'683'549,0 µm³

2.1.1. Chord Lenght Distribution

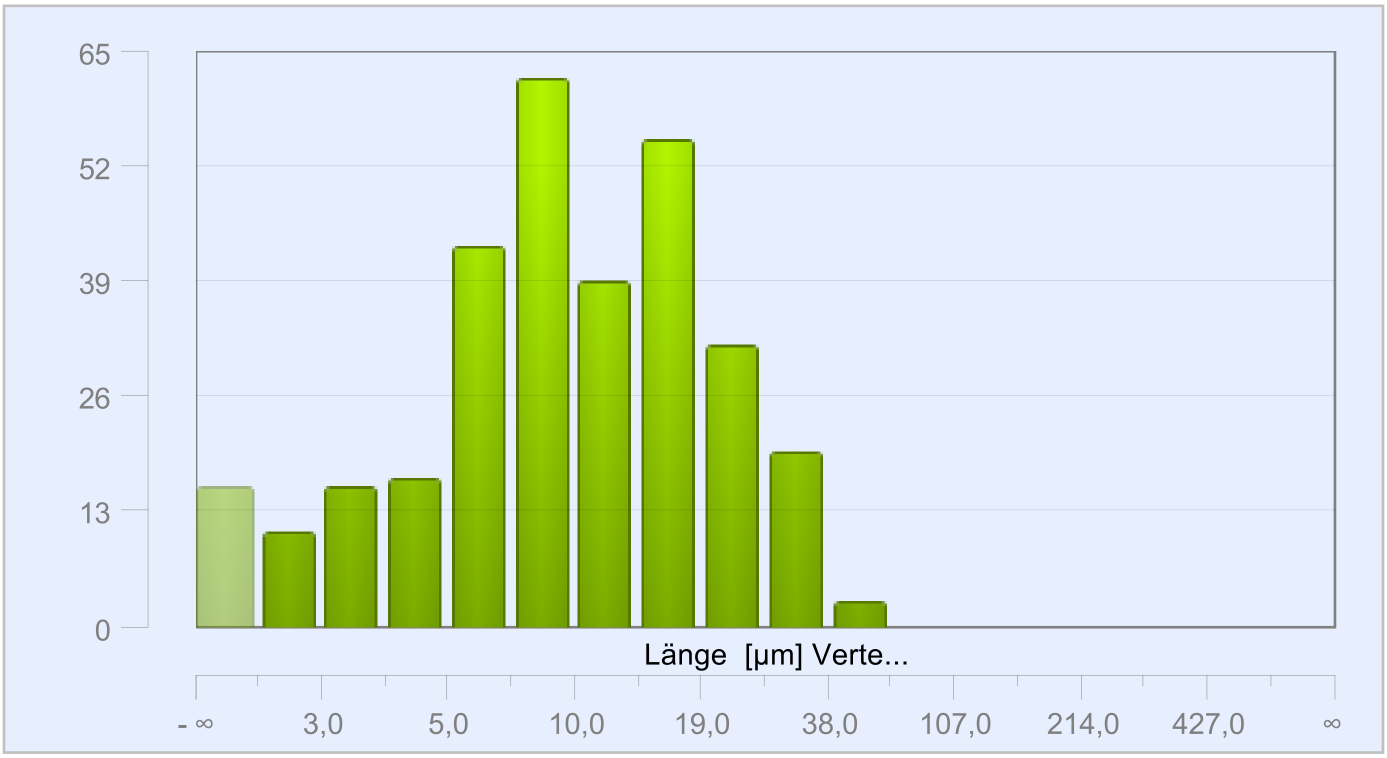

Start	Ende	Absolute Häufigkeit	Absolute Häufigkeit (kumuliert)	Relative Häufigkeit [%]	Relative Häufigkeit (kumuliert) [%]
	2,0 µm	16	16	5	5
2,0 µm	3,0 µm	11	27	4	9
3,0 µm	4,0 µm	16	43	5	14
4,0 µm	5,0 µm	17	60	5	19
5,0 µm	7,0 µm	43	103	14	33
7,0 µm	10,0 µm	62	165	20	53
10,0 µm	13,0 µm	39	204	12	65
13,0 µm	19,0 µm	55	259	18	82
19,0 µm	27,0 µm	32	291	10	93
27,0 µm	38,0 µm	20	311	6	99
38,0 µm	75,0 µm	3	314	1	100
75,0 µm	107,0 µm	0	314	0	100
107,0 µm	151,0 µm	0	314	0	100
151,0 µm	214,0 µm	0	314	0	100
214,0 µm	302,0 µm	0	314	0	100
302,0 µm	427,0 µm	0	314	0	100
427,0 µm	600,0 µm	0	314	0	100
600,0 µm		0	314	0	100

3. Single Result 2 (MnFeNi Semesterprojekt_MnFeNi_homogenized_8.1mmSW_800°C_45min_00107)

Mittlere Sehnenlänge	13,4 µm
Korngröße (ASTM)	9,2
Korngröße (G643)	9,1
Kornstreckung	86,3 %

3.1. Statistische Analyse

Statistische Daten	Länge
Anzahl Objekte	281
Minimum	1,5 µm
Maximum	113,6 µm
Mittelwert	13,4 µm
Standardabweichung	11,5 µm
Schiefe	0,0
Standardabweichung (n-1)	11,5 µm
Varianz	132,8 µm²
Varianz (n-1)	133,3 µm²
Summe	3'772,7 µm
Quadratsumme	87'973,7 µm²
Kubiksumme	3'735'766,2 µm³

3.1.1. Chord Lenght Distribution

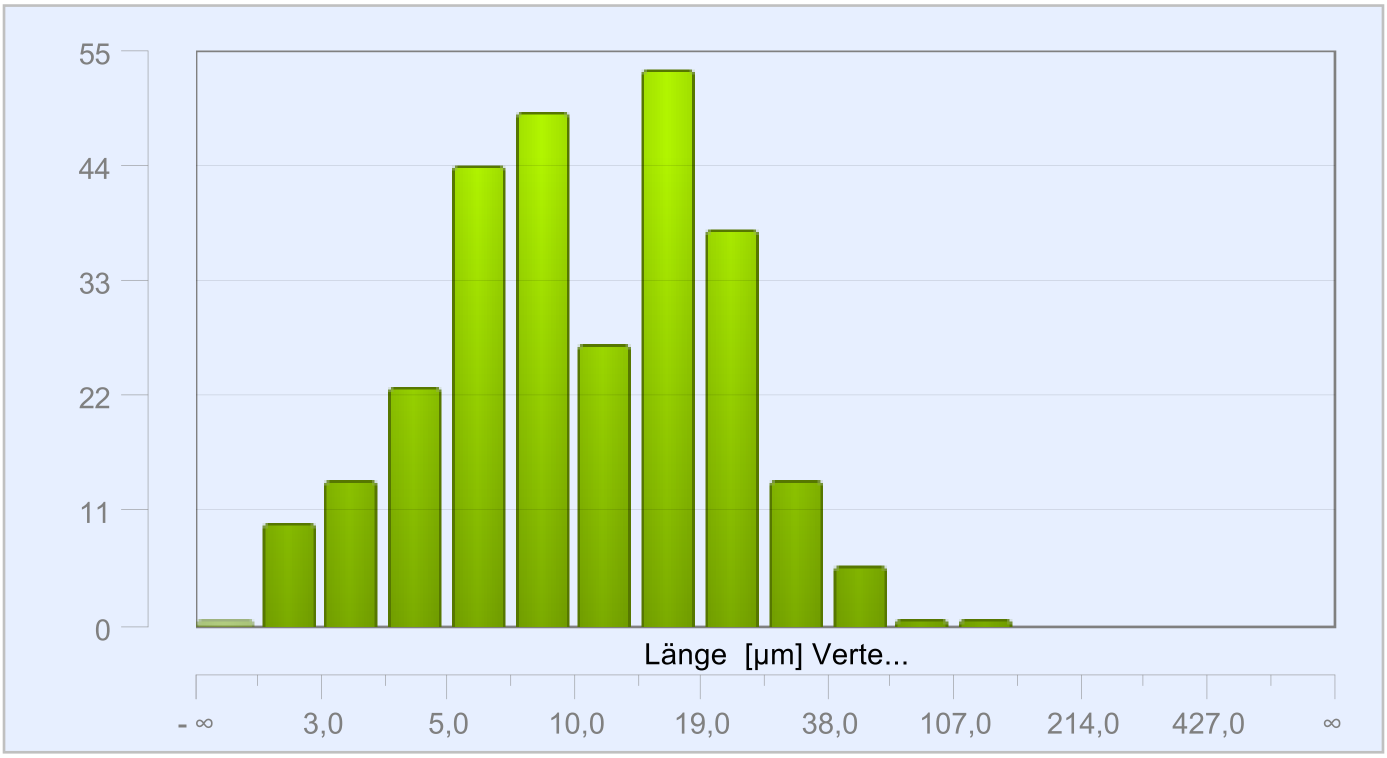

Start	Ende	Absolute Häufigkeit	Absolute Häufigkeit (kumuliert)	Relative Häufigkeit [%]	Relative Häufigkeit (kumuliert) [%]
	2,0 µm	1	1	0	0
2,0 µm	3,0 µm	10	11	4	4
3,0 µm	4,0 µm	14	25	5	9
4,0 µm	5,0 µm	23	48	8	17
5,0 µm	7,0 µm	44	92	16	33
7,0 µm	10,0 µm	49	141	17	50
10,0 µm	13,0 µm	27	168	10	60
13,0 µm	19,0 µm	53	221	19	79
19,0 µm	27,0 µm	38	259	14	92
27,0 µm	38,0 µm	14	273	5	97
38,0 µm	75,0 µm	6	279	2	99
75,0 µm	107,0 µm	1	280	0	100

Start	Ende	Absolute Häufigkeit	Absolute Häufigkeit (kumuliert)	Relative Häufigkeit [%]	Relative Häufigkeit (kumuliert) [%]
107,0 µm	151,0 µm	1	281	0	100
151,0 µm	214,0 µm	0	281	0	100
214,0 µm	302,0 µm	0	281	0	100
302,0 µm	427,0 µm	0	281	0	100
427,0 µm	600,0 µm	0	281	0	100
600,0 µm		0	281	0	100

4. Single Result 3 (MnFeNi Semesterprojekt_MnFeNi_homogenized_8.1mmSW_800°C_45min_00108)

Mittlere Sehnenlänge	12,2 µm
Korngröße (ASTM)	9,4
Korngröße (G643)	9,4
Kornstreckung	71,6 %

4.1. Statistische Analyse

Statistische Daten	Länge
Anzahl Objekte	308
Minimum	1,0 µm
Maximum	59,7 µm
Mittelwert	12,2 µm
Standardabweichung	8,1 µm
Schiefe	0,0
Standardabweichung (n-1)	8,1 µm
Varianz	65,4 µm²
Varianz (n-1)	65,7 µm²
Summe	3'768,3 µm
Quadratsumme	66'262,9 µm²
Kubiksumme	1'557'063,3 µm³

4.1.1. Chord Lenght Distribution

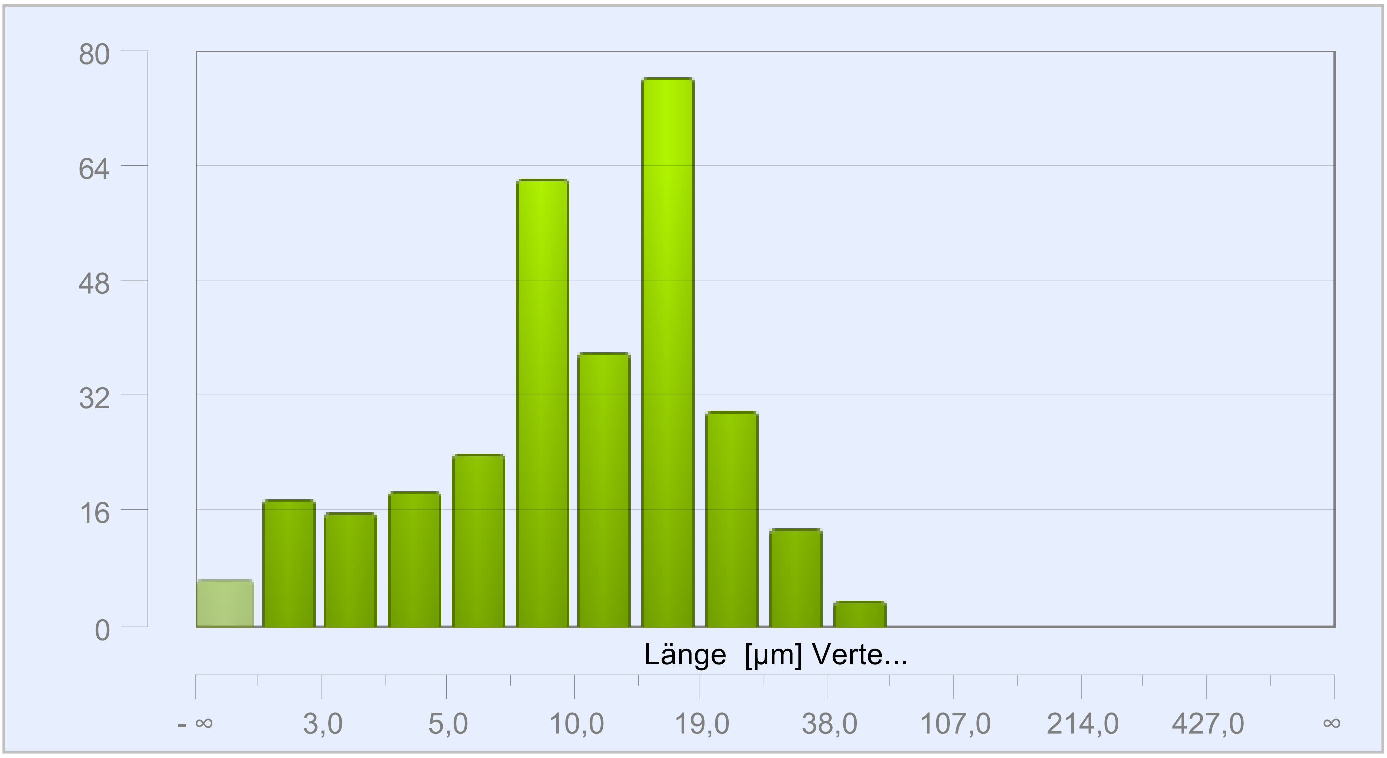

Start	Ende	Absolute Häufigkeit	Absolute Häufigkeit (kumuliert)	Relative Häufigkeit [%]	Relative Häufigkeit (kumuliert) [%]
	2,0 µm	7	7	2	2
2,0 µm	3,0 µm	18	25	6	8
3,0 µm	4,0 µm	16	41	5	13
4,0 µm	5,0 µm	19	60	6	19
5,0 µm	7,0 µm	24	84	8	27
7,0 µm	10,0 µm	62	146	20	47
10,0 µm	13,0 µm	38	184	12	60
13,0 µm	19,0 µm	76	260	25	84
19,0 µm	27,0 µm	30	290	10	94
27,0 µm	38,0 µm	14	304	5	99
38,0 µm	75,0 µm	4	308	1	100
75,0 µm	107,0 µm	0	308	0	100
107,0 µm	151,0 µm	0	308	0	100
151,0 µm	214,0 µm	0	308	0	100
214,0 µm	302,0 µm	0	308	0	100
302,0 µm	427,0 µm	0	308	0	100
427,0 µm	600,0 µm	0	308	0	100
600,0 µm		0	308	0	100

5. Single Result 4 (MnFeNi Semesterprojekt_MnFeNi_homogenized_8.1mmSW_800°C_45min_00109)

Mittlere Sehnenlänge	13,1 µm
Korngröße (ASTM)	9,2
Korngröße (G643)	9,2
Kornstreckung	82,8 %

5.1. Statistische Analyse

Statistische Daten	Länge
Anzahl Objekte	288
Minimum	0,7 µm
Maximum	64,4 µm
Mittelwert	13,1 µm
Standardabweichung	9,4 µm
Schiefe	0,0
Standardabweichung (n-1)	9,4 µm
Varianz	87,6 µm²
Varianz (n-1)	87,9 µm²
Summe	3'777,0 µm
Quadratsumme	74'752,5 µm²
Kubiksumme	2'055'387,4 µm³

5.1.1. Chord Lenght Distribution

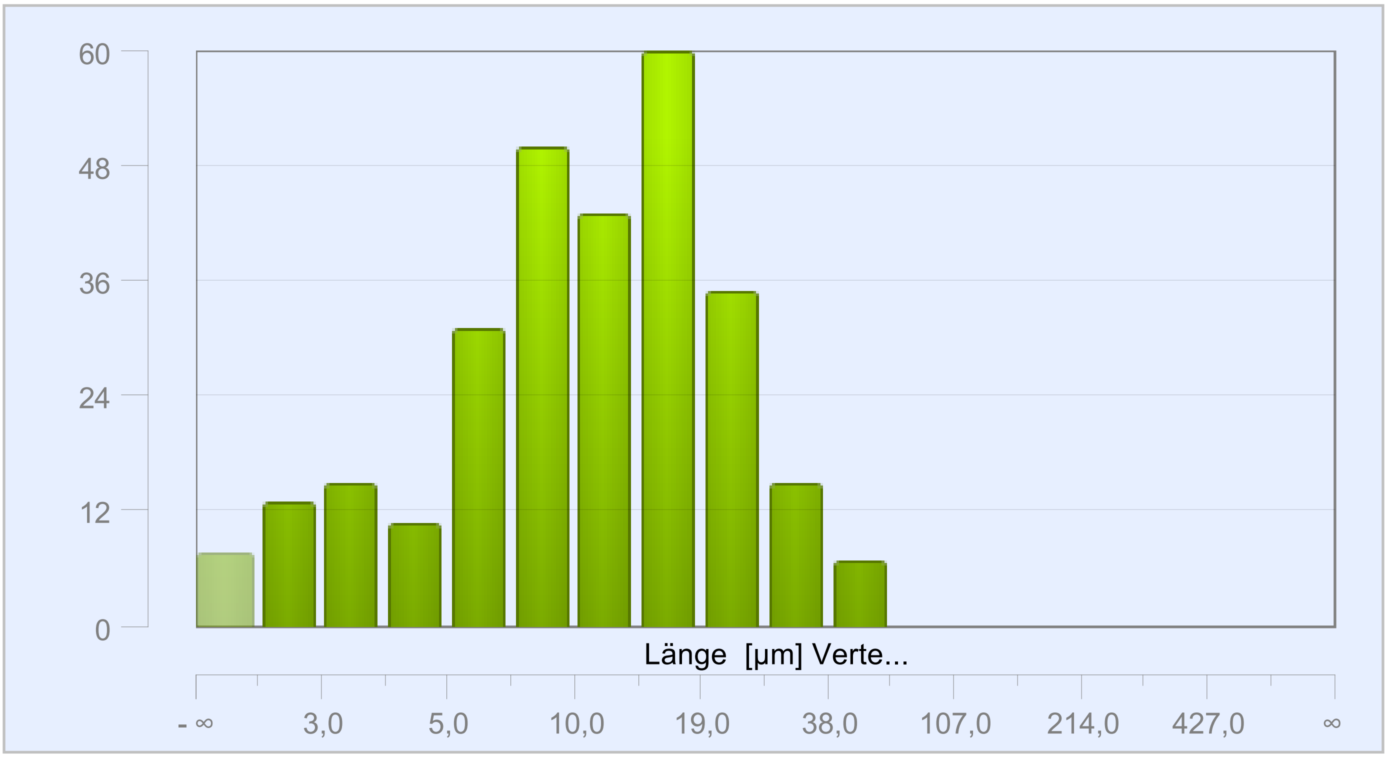

Start	Ende	Absolute Häufigkeit	Absolute Häufigkeit (kumuliert)	Relative Häufigkeit [%]	Relative Häufigkeit (kumuliert) [%]
	2,0 µm	8	8	3	3
2,0 µm	3,0 µm	13	21	5	7
3,0 µm	4,0 µm	15	36	5	12
4,0 µm	5,0 µm	11	47	4	16
5,0 µm	7,0 µm	31	78	11	27
7,0 µm	10,0 µm	50	128	17	44
10,0 µm	13,0 µm	43	171	15	59
13,0 µm	19,0 µm	60	231	21	80
19,0 µm	27,0 µm	35	266	12	92
27,0 µm	38,0 µm	15	281	5	98
38,0 µm	75,0 µm	7	288	2	100
75,0 µm	107,0 µm	0	288	0	100
107,0 µm	151,0 µm	0	288	0	100
151,0 µm	214,0 µm	0	288	0	100
214,0 µm	302,0 µm	0	288	0	100
302,0 µm	427,0 µm	0	288	0	100
427,0 µm	600,0 µm	0	288	0	100
600,0 µm		0	288	0	100
