# Supplementary material for: Data compilation on the effect of grain size, temperature, and texture on the strength of a single-phase FCC MnFeNi medium-entropy alloy
Source: Data Brief. 2019 Nov 15;28:104807. doi: 10.1016/j.dib.2019.104807 (PMC6909151; doi:10.1016/j.dib.2019.104807)
Supplement: Multimedia component 1 [file mmc1.zip › MnFeNi_1073K_45min/MnFeNi_1073K_45min_d=17μm.pdf]

Analysebericht

Jun 14, 2018 2:19:12 PM

powered by imagic.ch

1. cumulative Result 1

Number of images	4
Grain size (ASTM)	8.6
Grain size (G643)	8.5
Grain stretching	93 %
Mean chord length	16.5 µm

2. Single Result 1 (MnFeNi Semesterprojekt_MnFeNi_homogenized_8.1mmSW_800°C_45min_00106)

Mean chord length	15.9 µm
Grain size (ASTM)	8.7
Grain size (G643)	8.6
Grain stretching	91.8 %

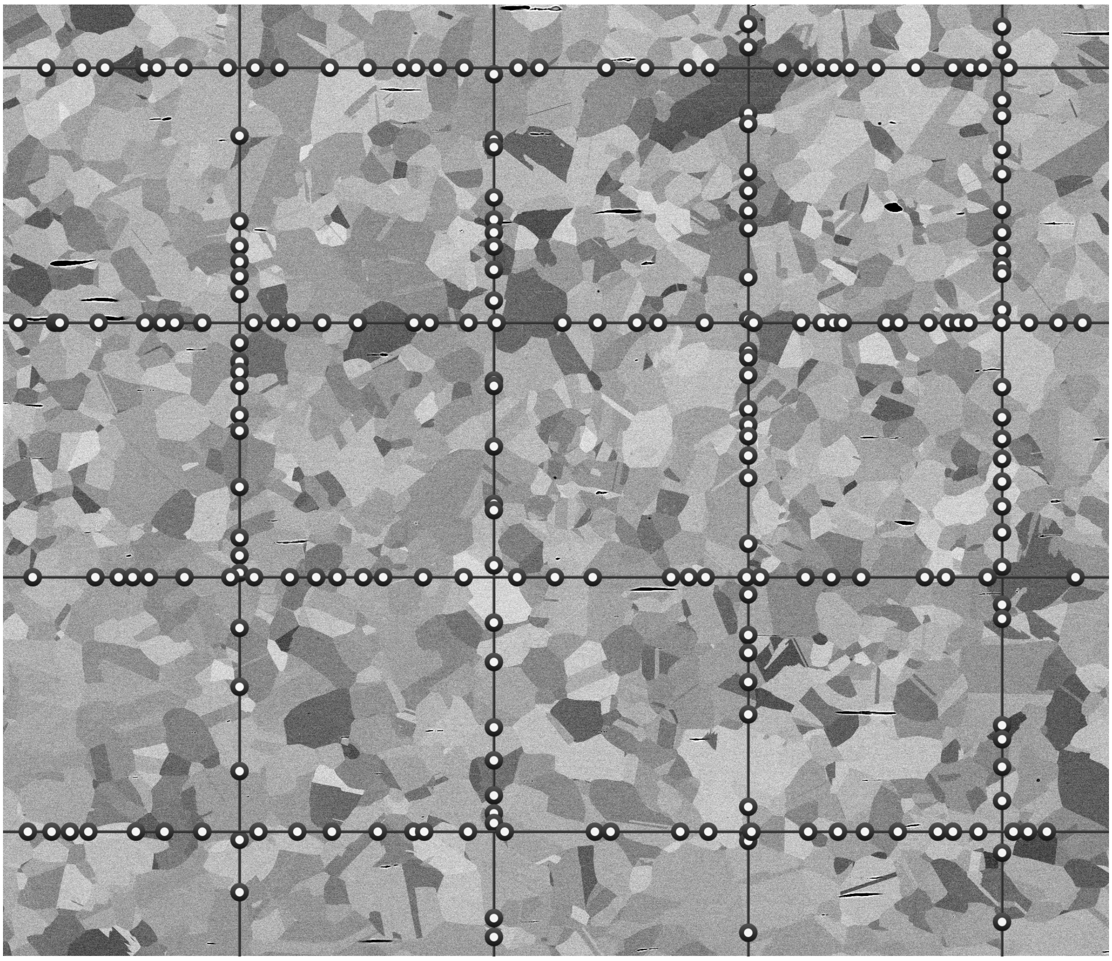

2.1. Statistical Analysis

Statistical Data	Length
Object Count	238
Minimum	2.2 µm
Maximum	60.3 µm
Average	15.9 µm
Standard deviation	9.7 µm
Skewness	0.0
Standard deviation (n-1)	9.7 µm
Variance	93.9 µm²
Variance (n-1)	94.3 µm²
Sum	3'779.7 µm

Statistical Data	Length
Sum of squares	82'378.6 µm²
Sum of cubes	2'309'993.3 µm³

2.1.1. Chord Lenght Distribution

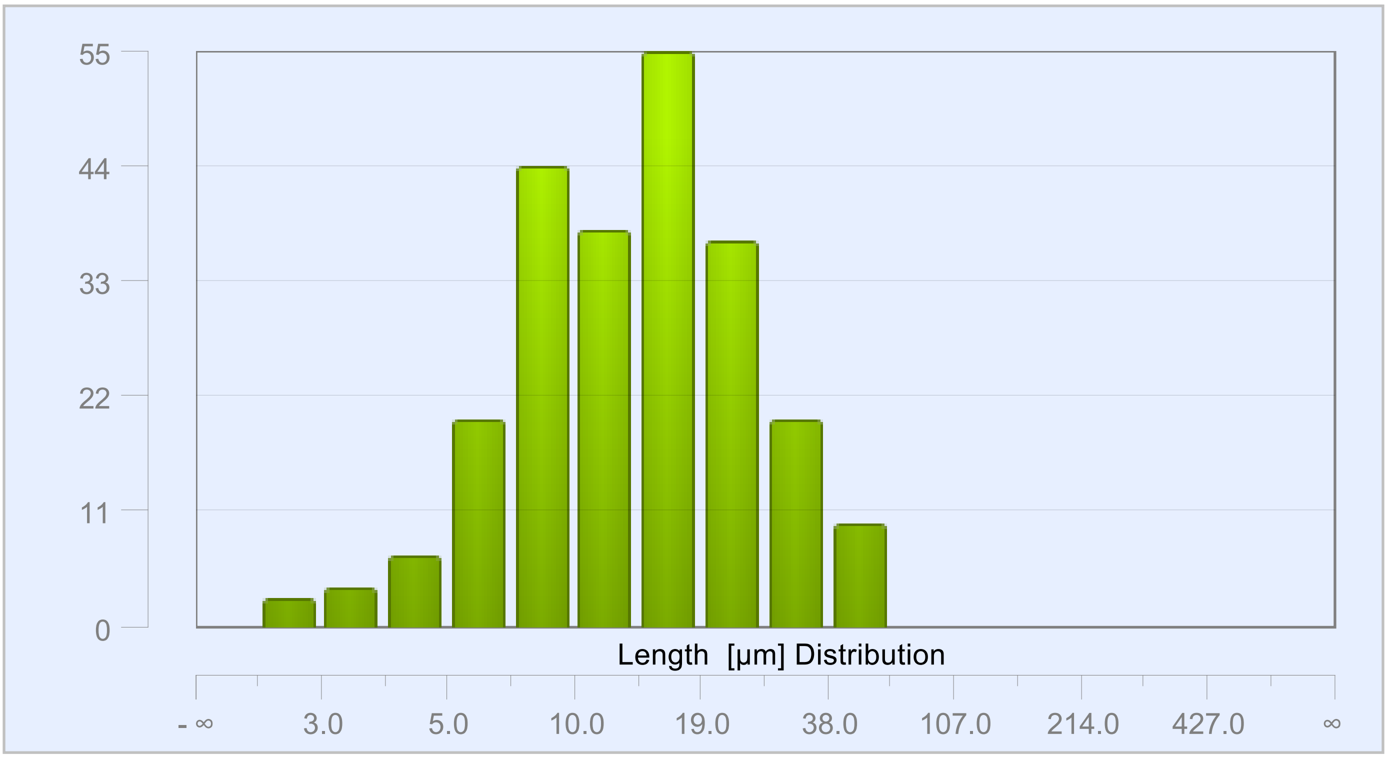

Start	End	Absolute Frequency	Absolute Frequency (accumulated)	Relative Frequency [%]	Relative Frequency (accumulated) [%]
	2.0 µm	0	0	0	0
2.0 µm	3.0 µm	3	3	1	1
3.0 µm	4.0 µm	4	7	2	3
4.0 µm	5.0 µm	7	14	3	6
5.0 µm	7.0 µm	20	34	8	14
7.0 µm	10.0 µm	44	78	18	33
10.0 µm	13.0 µm	38	116	16	49
13.0 µm	19.0 µm	55	171	23	72
19.0 µm	27.0 µm	37	208	16	87
27.0 µm	38.0 µm	20	228	8	96
38.0 µm	75.0 µm	10	238	4	100
75.0 µm	107.0 µm	0	238	0	100
107.0 µm	151.0 µm	0	238	0	100
151.0 µm	214.0 µm	0	238	0	100
214.0 µm	302.0 µm	0	238	0	100
302.0 µm	427.0 µm	0	238	0	100
427.0 µm	600.0 µm	0	238	0	100
600.0 µm		0	238	0	100

3. Single Result 2 (MnFeNi Semesterprojekt_MnFeNi_homogenized_8.1mmSW_800°C_45min_00107)

Mean chord length	17.4 µm
Grain size (ASTM)	8.4
Grain size (G643)	8.4
Grain stretching	89.7 %

3.1. Statistical Analysis

Statistical Data	Length
Object Count	217
Minimum	1.0 µm
Maximum	79.7 µm
Average	17.4 µm
Standard deviation	11.8 µm
Skewness	0.0
Standard deviation (n-1)	11.9 µm
Variance	140.0 µm²
Variance (n-1)	140.6 µm²
Sum	3'772.7 µm
Sum of squares	95'971.2 µm²
Sum of cubes	3'306'709.8 µm³

3.1.1. Chord Lenght Distribution

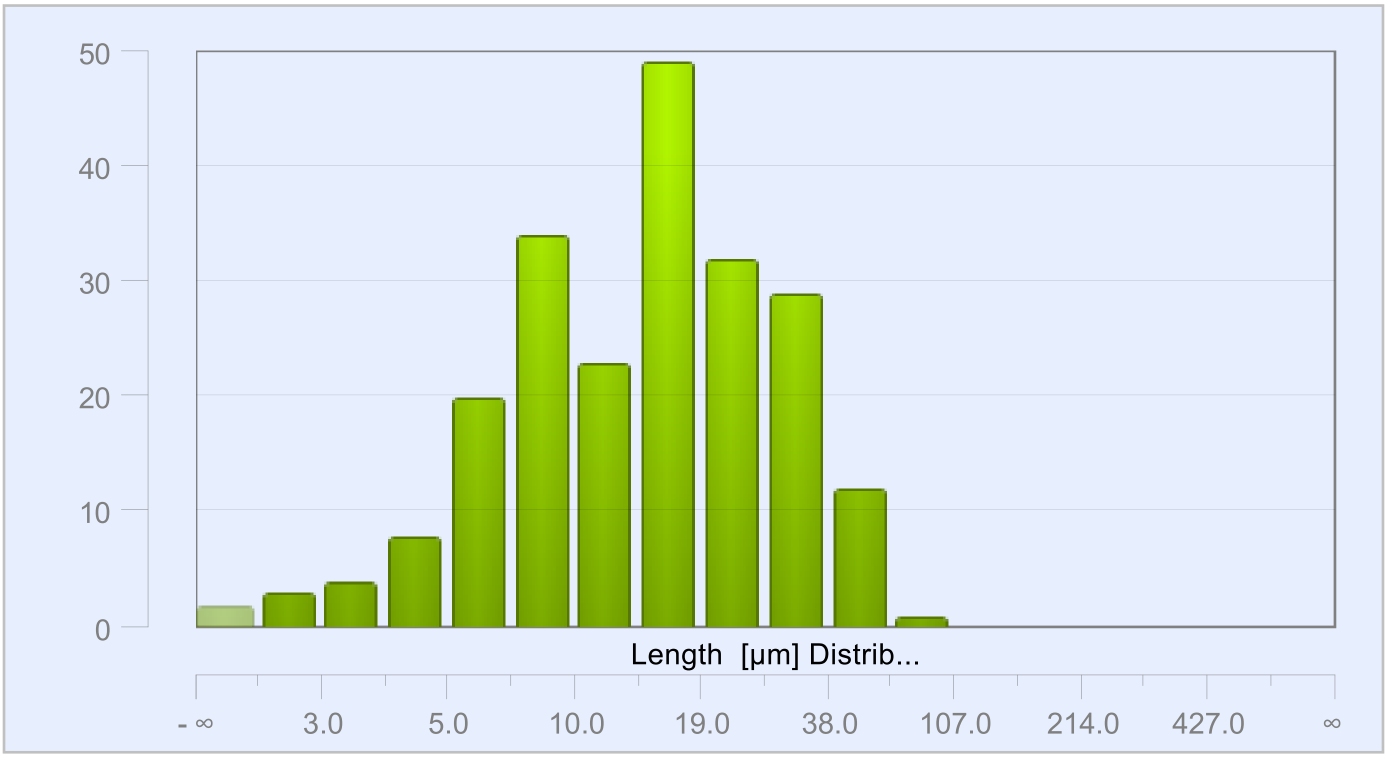

Start	End	Absolute Frequency	Absolute Frequency (accumulated)	Relative Frequency [%]	Relative Frequency (accumulated) [%]
	2.0 µm	2	2	1	1
2.0 µm	3.0 µm	3	5	1	2
3.0 µm	4.0 µm	4	9	2	4
4.0 µm	5.0 µm	8	17	4	8
5.0 µm	7.0 µm	20	37	9	17
7.0 µm	10.0 µm	34	71	16	33
10.0 µm	13.0 µm	23	94	11	43
13.0 µm	19.0 µm	49	143	23	66
19.0 µm	27.0 µm	32	175	15	81
27.0 µm	38.0 µm	29	204	13	94
38.0 µm	75.0 µm	12	216	6	100
75.0 µm	107.0 µm	1	217	0	100

Start	End	Absolute Frequency	Absolute Frequency (accumulated)	Relative Frequency [%]	Relative Frequency (accumulated) [%]
107.0 µm	151.0 µm	0	217	0	100
151.0 µm	214.0 µm	0	217	0	100
214.0 µm	302.0 µm	0	217	0	100
302.0 µm	427.0 µm	0	217	0	100
427.0 µm	600.0 µm	0	217	0	100
600.0 µm		0	217	0	100

4. Single Result 3 (MnFeNi Semesterprojekt_MnFeNi_homogenized_8.1mmSW_800°C_45min_00108)

Mean chord length	16.3 µm
Grain size (ASTM)	8.6
Grain size (G643)	8.5
Grain stretching	80.9 %

4.1. Statistical Analysis

Statistical Data	Length
Object Count	231
Minimum	2.0 µm
Maximum	61.9 µm
Average	16.3 µm
Standard deviation	9.5 µm
Skewness	0.0
Standard deviation (n-1)	9.5 µm
Variance	89.8 µm²
Variance (n-1)	90.2 µm²
Sum	3'768.3 µm
Sum of squares	82'216.3 µm²
Sum of cubes	2'322'530.7 µm³

4.1.1. Chord Lenght Distribution

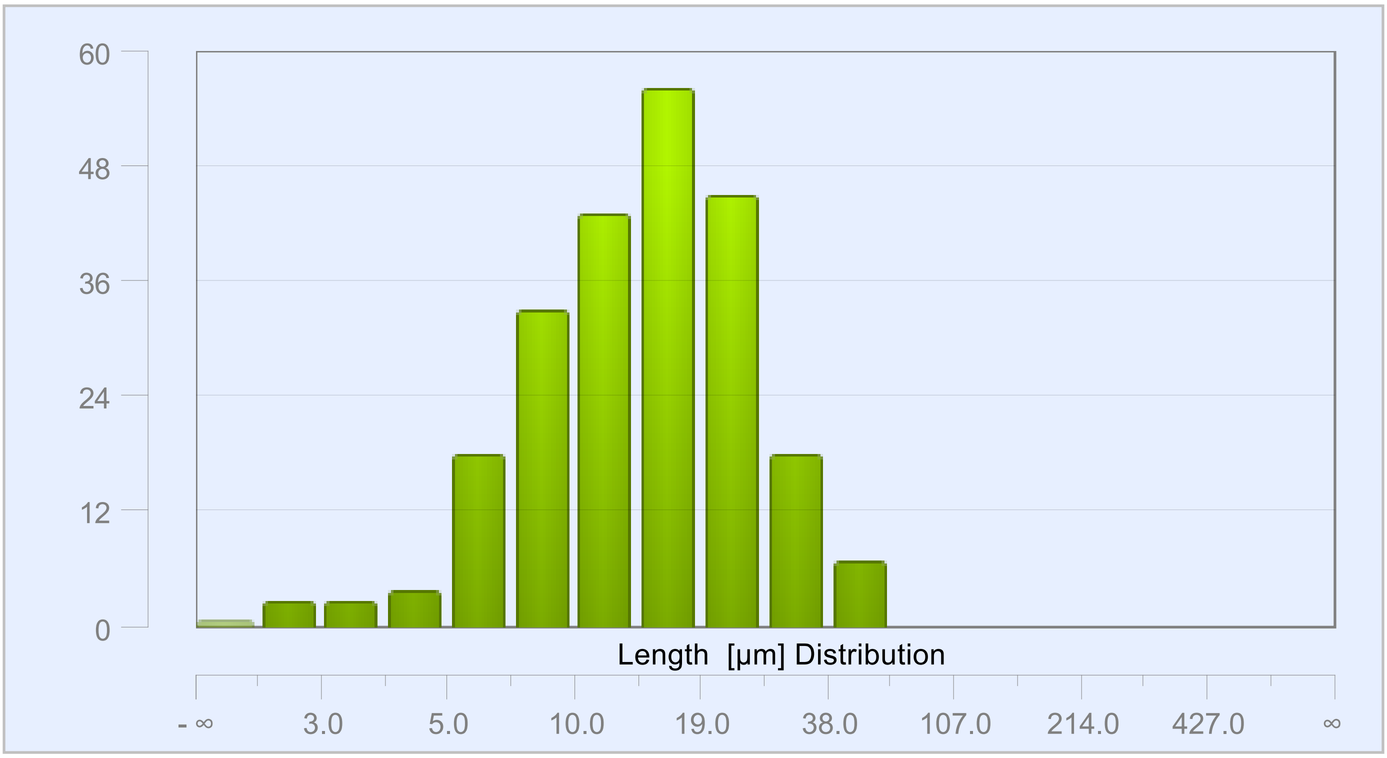

Start	End	Absolute Frequency	Absolute Frequency (accumulated)	Relative Frequency [%]	Relative Frequency (accumulated) [%]
	2.0 µm	1	1	0	0
2.0 µm	3.0 µm	3	4	1	2
3.0 µm	4.0 µm	3	7	1	3
4.0 µm	5.0 µm	4	11	2	5
5.0 µm	7.0 µm	18	29	8	13
7.0 µm	10.0 µm	33	62	14	27
10.0 µm	13.0 µm	43	105	19	45
13.0 µm	19.0 µm	56	161	24	70
19.0 µm	27.0 µm	45	206	19	89
27.0 µm	38.0 µm	18	224	8	97
38.0 µm	75.0 µm	7	231	3	100
75.0 µm	107.0 µm	0	231	0	100
107.0 µm	151.0 µm	0	231	0	100
151.0 µm	214.0 µm	0	231	0	100
214.0 µm	302.0 µm	0	231	0	100
302.0 µm	427.0 µm	0	231	0	100
427.0 µm	600.0 µm	0	231	0	100
600.0 µm		0	231	0	100

5. Single Result 4 (MnFeNi Semesterprojekt_MnFeNi_homogenized_8.1mmSW_800°C_45min_00109)

Mean chord length	16.6 µm
Grain size (ASTM)	8.5
Grain size (G643)	8.5
Grain stretching	94.2 %

5.1. Statistical Analysis

Statistical Data	Length
Object Count	228
Minimum	1.0 µm
Maximum	89.0 µm
Average	16.6 µm
Standard deviation	10.2 µm
Skewness	0.0
Standard deviation (n-1)	10.2 µm
Variance	104.5 µm²
Variance (n-1)	105.0 µm²
Sum	3'777.0 µm
Sum of squares	86'397.1 µm²
Sum of cubes	2'717'125.5 µm³

5.1.1. Chord Lenght Distribution

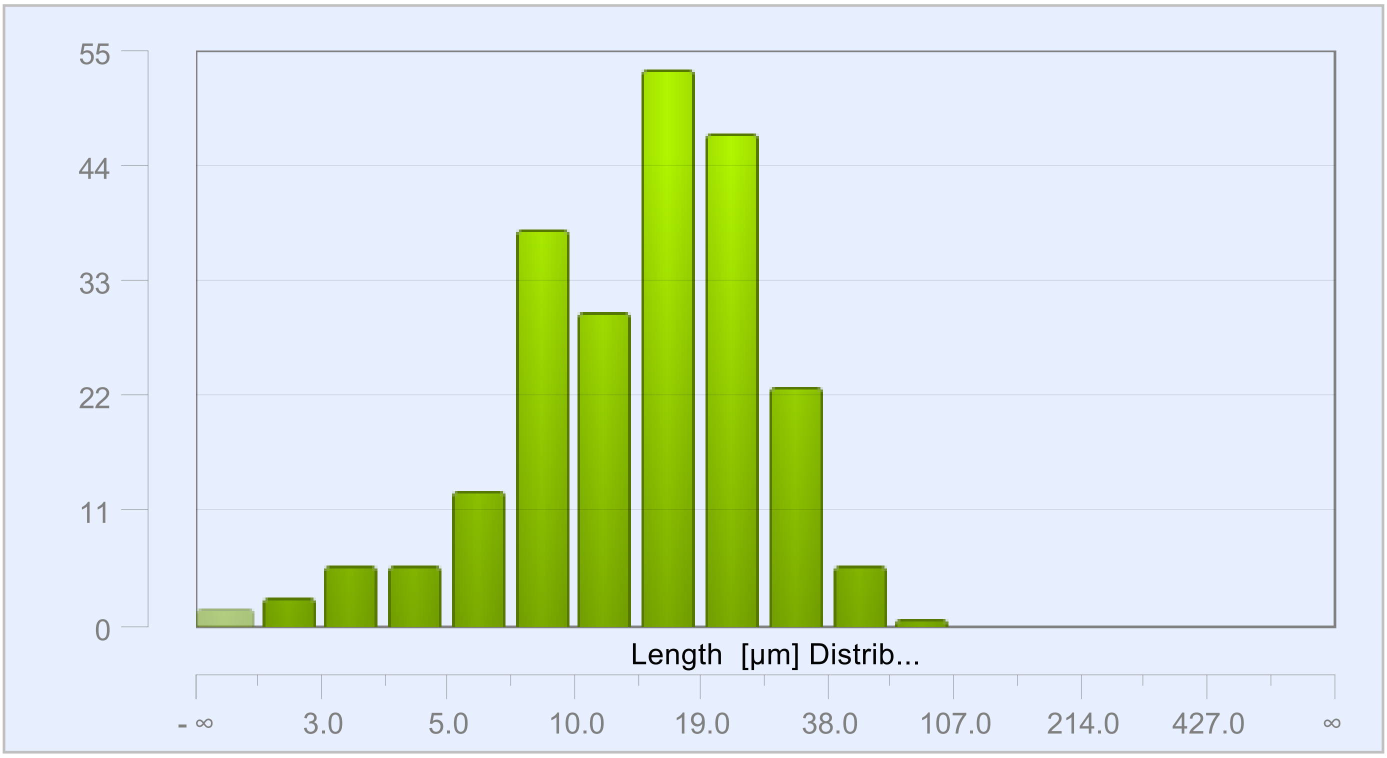

Start	End	Absolute Frequency	Absolute Frequency (accumulated)	Relative Frequency [%]	Relative Frequency (accumulated) [%]
	2.0 µm	2	2	1	1
2.0 µm	3.0 µm	3	5	1	2
3.0 µm	4.0 µm	6	11	3	5
4.0 µm	5.0 µm	6	17	3	7
5.0 µm	7.0 µm	13	30	6	13
7.0 µm	10.0 µm	38	68	17	30
10.0 µm	13.0 µm	30	98	13	43
13.0 µm	19.0 µm	53	151	23	66
19.0 µm	27.0 µm	47	198	21	87
27.0 µm	38.0 µm	23	221	10	97
38.0 µm	75.0 µm	6	227	3	100
75.0 µm	107.0 µm	1	228	0	100
107.0 µm	151.0 µm	0	228	0	100
151.0 µm	214.0 µm	0	228	0	100
214.0 µm	302.0 µm	0	228	0	100
302.0 µm	427.0 µm	0	228	0	100
427.0 µm	600.0 µm	0	228	0	100
600.0 µm		0	228	0	100
