# Supplementary material for: Data compilation on the effect of grain size, temperature, and texture on the strength of a single-phase FCC MnFeNi medium-entropy alloy
Source: Data Brief. 2019 Nov 15;28:104807. doi: 10.1016/j.dib.2019.104807 (PMC6909151; doi:10.1016/j.dib.2019.104807)
Supplement: Multimedia component 1 [file mmc1.zip › MnFeNi_1073K_60min/MnFeNi_1073K_60min_c=16μm.pdf]

Analysebericht

Mar 1, 2018 4:07:44 PM

powered by imagic.ch

1. cumulative Result 1

Number of images	4
Grain size (ASTM)	8.6
Grain size (G643)	8.6
Grain stretching	92.3 %
Mean chord length	16 µm

2. Single Result 1 (MnFeNi Semesterprojekt_MnFeNi_homogenized_8.1mmSW_800°C_60min_00039)

Mean chord length	15.8 µm
Grain size (ASTM)	8.7
Grain size (G643)	8.6
Grain stretching	85.3 %

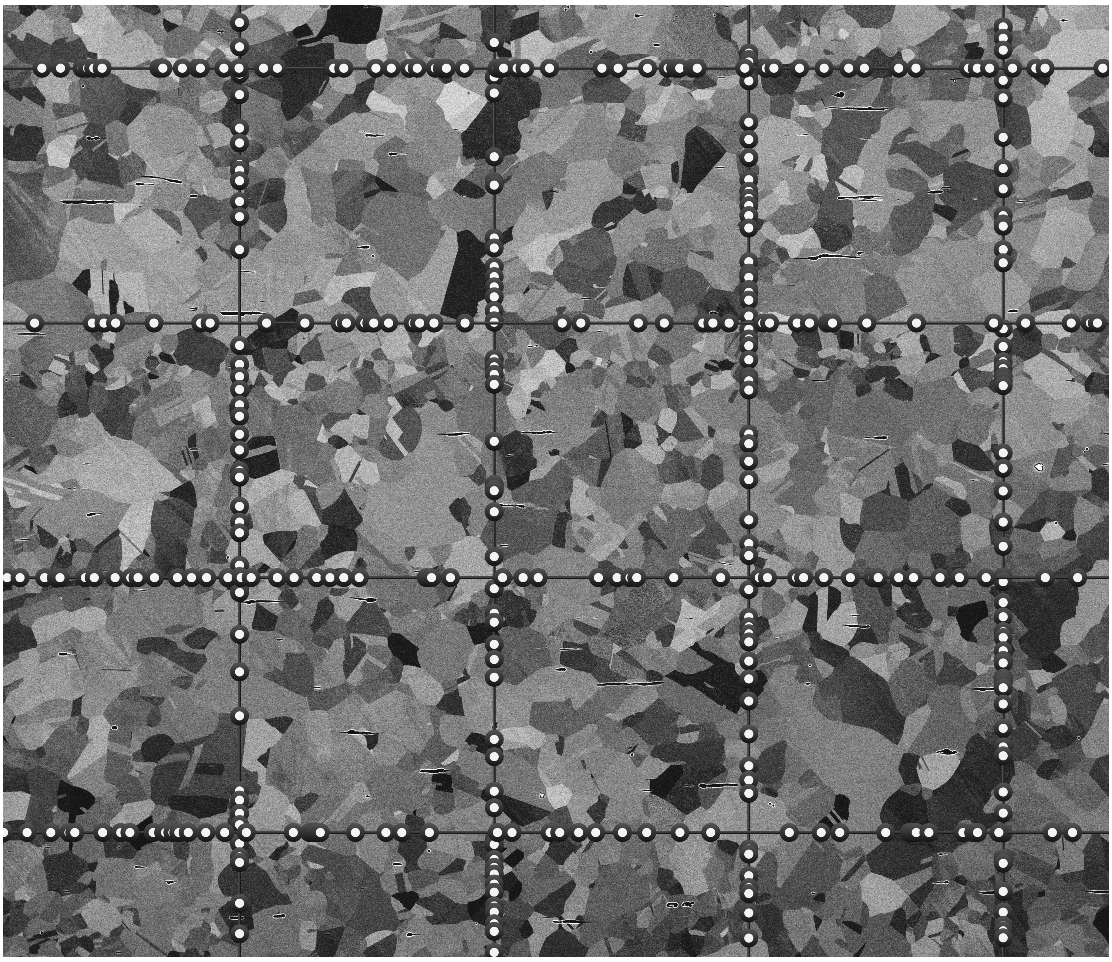

2.1. Statistical Analysis

Statistical Data	Length
Object Count	400
Minimum	0.6 µm
Maximum	74.5 µm
Average	15.8 µm
Standard deviation	12.2 µm
Skewness	0.0
Standard deviation (n-1)	12.2 µm
Variance	148.3 µm²
Variance (n-1)	148.7 µm²
Sum	6'305.7 µm

Statistical Data	Length
Sum of squares	158'733.3 µm²
Sum of cubes	5'536'864.8 µm³

2.1.1. Chord Lenght Distribution

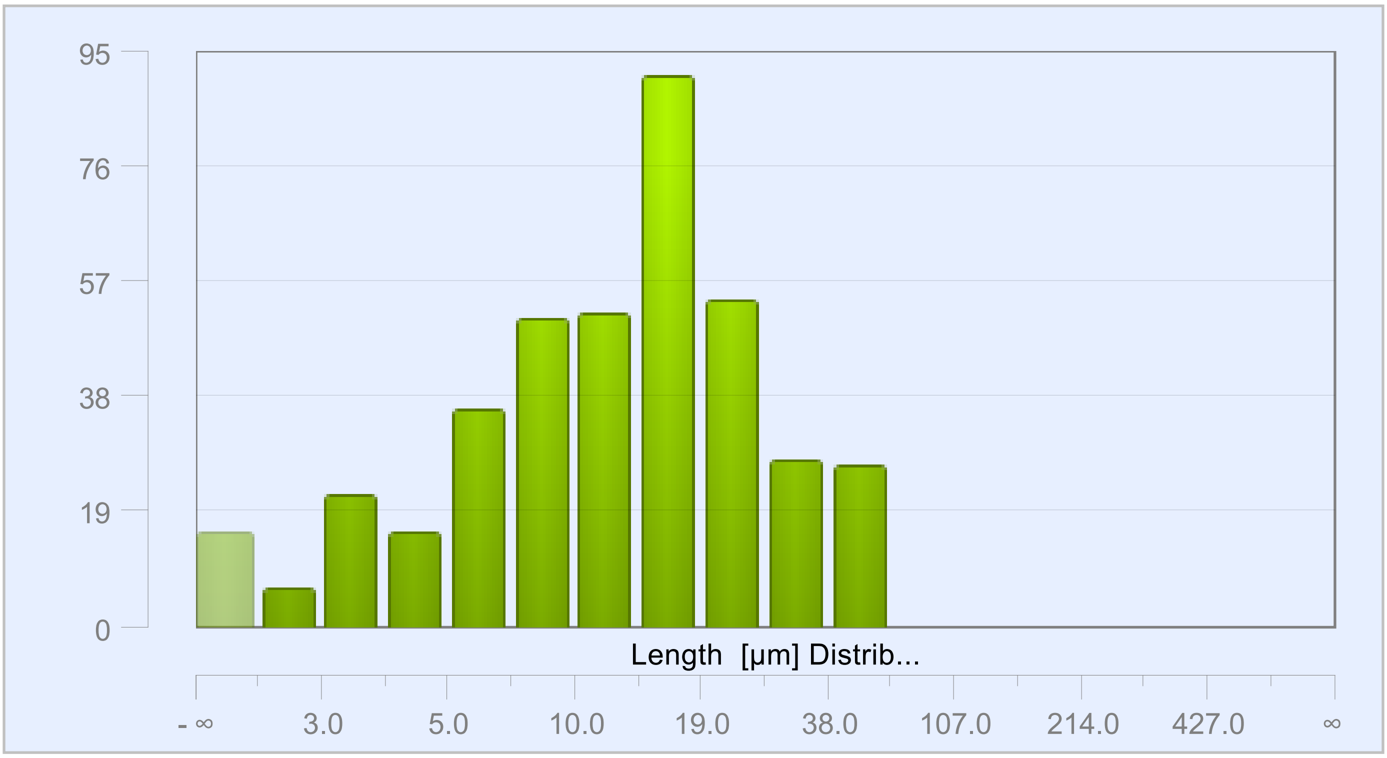

Start	End	Absolute Frequency	Absolute Frequency (accumulated)	Relative Frequency [%]	Relative Frequency (accumulated) [%]
	2.0 µm	16	16	4	4
2.0 µm	3.0 µm	7	23	2	6
3.0 µm	4.0 µm	22	45	6	11
4.0 µm	5.0 µm	16	61	4	15
5.0 µm	7.0 µm	36	97	9	24
7.0 µm	10.0 µm	51	148	13	37
10.0 µm	13.0 µm	52	200	13	50
13.0 µm	19.0 µm	91	291	23	73
19.0 µm	27.0 µm	54	345	14	86
27.0 µm	38.0 µm	28	373	7	93
38.0 µm	75.0 µm	27	400	7	100
75.0 µm	107.0 µm	0	400	0	100
107.0 µm	151.0 µm	0	400	0	100
151.0 µm	214.0 µm	0	400	0	100
214.0 µm	302.0 µm	0	400	0	100
302.0 µm	427.0 µm	0	400	0	100
427.0 µm	600.0 µm	0	400	0	100
600.0 µm		0	400	0	100

3. Single Result 2 (MnFeNi Semesterprojekt_MnFeNi_homogenized_8.1mmSW_800°C_60min_00057)

Mean chord length	15.5 µm
Grain size (ASTM)	8.7
Grain size (G643)	8.7
Grain stretching	96.7 %

3.1. Statistical Analysis

Statistical Data	Length
Object Count	407
Minimum	1.0 µm
Maximum	64.9 µm
Average	15.5 µm
Standard deviation	11.3 µm
Skewness	0.0
Standard deviation (n-1)	11.3 µm
Variance	127.6 µm²
Variance (n-1)	127.9 µm²
Sum	6'300.0 µm
Sum of squares	149'464.9 µm²
Sum of cubes	4'708'671.2 µm³

3.1.1. Chord Lenght Distribution

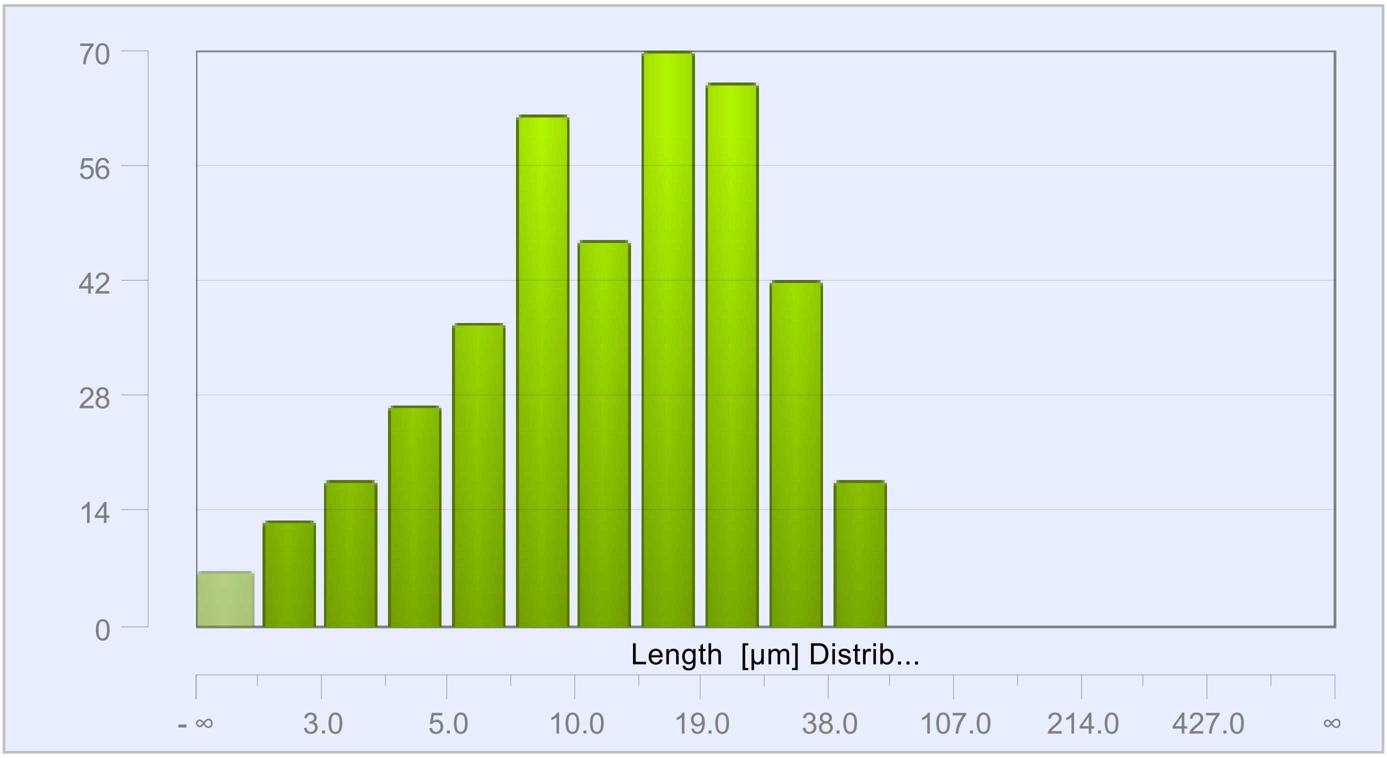

Start	End	Absolute Frequency	Absolute Frequency (accumulated)	Relative Frequency [%]	Relative Frequency (accumulated) [%]
	2.0 µm	7	7	2	2
2.0 µm	3.0 µm	13	20	3	5
3.0 µm	4.0 µm	18	38	4	9
4.0 µm	5.0 µm	27	65	7	16
5.0 µm	7.0 µm	37	102	9	25
7.0 µm	10.0 µm	62	164	15	40
10.0 µm	13.0 µm	47	211	12	52
13.0 µm	19.0 µm	70	281	17	69
19.0 µm	27.0 µm	66	347	16	85
27.0 µm	38.0 µm	42	389	10	96
38.0 µm	75.0 µm	18	407	4	100
75.0 µm	107.0 µm	0	407	0	100

Start	End	Absolute Frequency	Absolute Frequency (accumulated)	Relative Frequency [%]	Relative Frequency (accumulated) [%]
107.0 µm	151.0 µm	0	407	0	100
151.0 µm	214.0 µm	0	407	0	100
214.0 µm	302.0 µm	0	407	0	100
302.0 µm	427.0 µm	0	407	0	100
427.0 µm	600.0 µm	0	407	0	100
600.0 µm		0	407	0	100

4. Single Result 3 (MnFeNi Semesterprojekt_MnFeNi_homogenized_8.1mmSW_800°C_60min_00058)

Mean chord length	15.7 µm
Grain size (ASTM)	8.7
Grain size (G643)	8.7
Grain stretching	93.2 %

4.1. Statistical Analysis

Statistical Data	Length
Object Count	402
Minimum	1.0 µm
Maximum	83.1 µm
Average	15.7 µm
Standard deviation	13.4 µm
Skewness	0.0
Standard deviation (n-1)	13.5 µm
Variance	180.5 µm²
Variance (n-1)	181.0 µm²
Sum	6'299.9 µm
Sum of squares	171'290.7 µm²
Sum of cubes	6'938'674.4 µm³

4.1.1. Chord Lenght Distribution

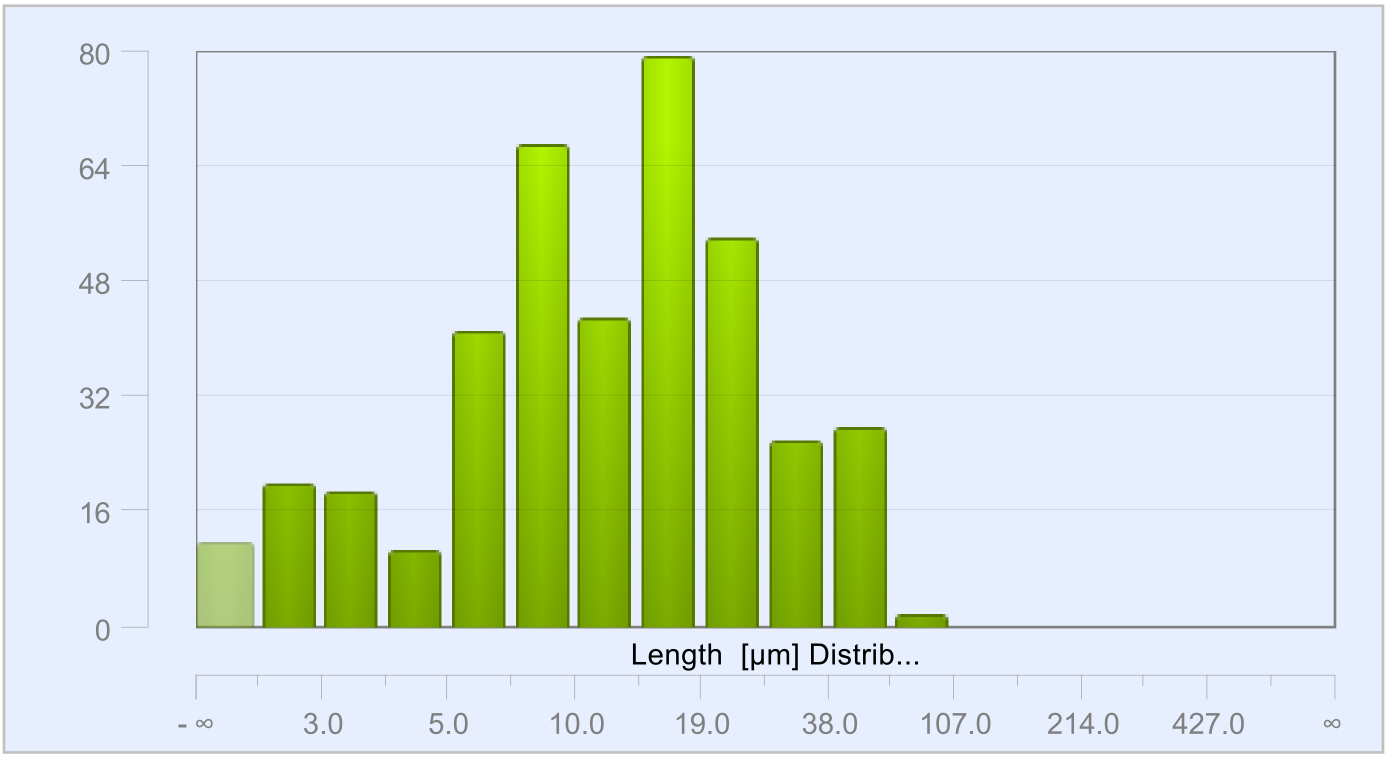

Start	End	Absolute Frequency	Absolute Frequency (accumulated)	Relative Frequency [%]	Relative Frequency (accumulated) [%]
	2.0 µm	12	12	3	3
2.0 µm	3.0 µm	20	32	5	8
3.0 µm	4.0 µm	19	51	5	13
4.0 µm	5.0 µm	11	62	3	15
5.0 µm	7.0 µm	41	103	10	26
7.0 µm	10.0 µm	67	170	17	42
10.0 µm	13.0 µm	43	213	11	53
13.0 µm	19.0 µm	79	292	20	73
19.0 µm	27.0 µm	54	346	13	86
27.0 µm	38.0 µm	26	372	6	93
38.0 µm	75.0 µm	28	400	7	100
75.0 µm	107.0 µm	2	402	0	100
107.0 µm	151.0 µm	0	402	0	100
151.0 µm	214.0 µm	0	402	0	100
214.0 µm	302.0 µm	0	402	0	100
302.0 µm	427.0 µm	0	402	0	100
427.0 µm	600.0 µm	0	402	0	100
600.0 µm		0	402	0	100

5. Single Result 4 (MnFeNi Semesterprojekt_MnFeNi_homogenized_8.1mmSW_800°C_60min_00059)

Mean chord length	17.3 µm
Grain size (ASTM)	8.4
Grain size (G643)	8.4
Grain stretching	87.8 %

5.1. Statistical Analysis

Statistical Data	Length
Object Count	364
Minimum	0.8 µm
Maximum	96.1 µm
Average	17.3 µm
Standard deviation	13.6 µm
Skewness	0.0
Standard deviation (n-1)	13.7 µm
Variance	186.3 µm²
Variance (n-1)	186.8 µm²
Sum	6'295.8 µm
Sum of squares	176'712.5 µm²
Sum of cubes	7'147'818.6 µm³

5.1.1. Chord Lenght Distribution

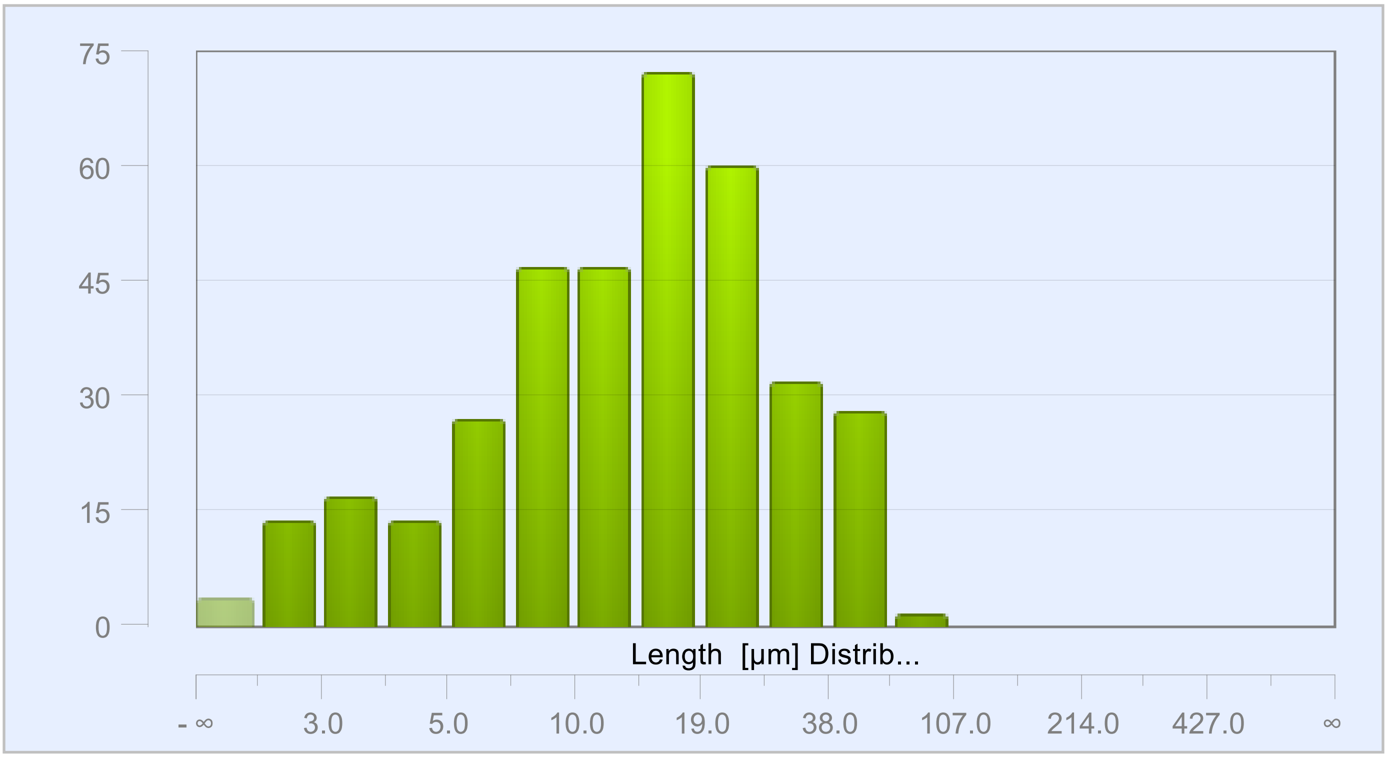

Start	End	Absolute Frequency	Absolute Frequency (accumulated)	Relative Frequency [%]	Relative Frequency (accumulated) [%]
	2.0 µm	4	4	1	1
2.0 µm	3.0 µm	14	18	4	5
3.0 µm	4.0 µm	17	35	5	10
4.0 µm	5.0 µm	14	49	4	13
5.0 µm	7.0 µm	27	76	7	21
7.0 µm	10.0 µm	47	123	13	34
10.0 µm	13.0 µm	47	170	13	47
13.0 µm	19.0 µm	72	242	20	66
19.0 µm	27.0 µm	60	302	16	83
27.0 µm	38.0 µm	32	334	9	92
38.0 µm	75.0 µm	28	362	8	99
75.0 µm	107.0 µm	2	364	1	100
107.0 µm	151.0 µm	0	364	0	100
151.0 µm	214.0 µm	0	364	0	100
214.0 µm	302.0 µm	0	364	0	100
302.0 µm	427.0 µm	0	364	0	100
427.0 µm	600.0 µm	0	364	0	100
600.0 µm		0	364	0	100
