# Supplementary material for: Data compilation on the effect of grain size, temperature, and texture on the strength of a single-phase FCC MnFeNi medium-entropy alloy
Source: Data Brief. 2019 Nov 15;28:104807. doi: 10.1016/j.dib.2019.104807 (PMC6909151; doi:10.1016/j.dib.2019.104807)
Supplement: Multimedia component 1 [file mmc1.zip › MnFeNi_1073K_60min/MnFeNi_1073K_60min_d=19μm.pdf]

Analysebericht

Feb 28, 2018 4:01:51 PM

powered by imagic.ch

1. cumulative Result 1

Number of images	4
Grain size (ASTM)	8.2
Grain size (G643)	8.2
Grain stretching	98.2 %
Mean chord length	18.5 µm

2. Single Result 1 (MnFeNi Semesterprojekt_MnFeNi_homogenized_8.1mmSW_800°C_60min_00039)

Mean chord length	17.6 µm
Grain size (ASTM)	8.4
Grain size (G643)	8.3
Grain stretching	96 %

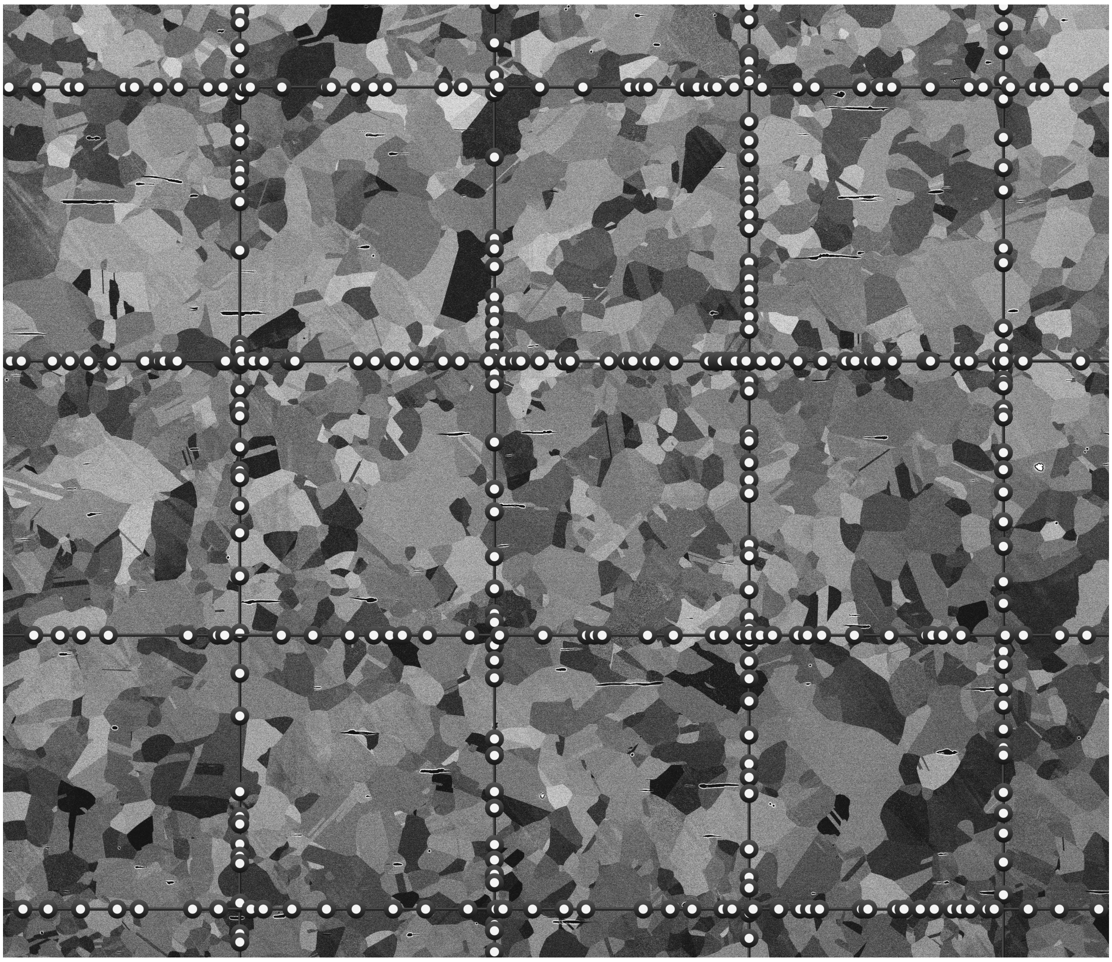

2.1. Statistical Analysis

Statistical Data	Length
Object Count	358
Minimum	0.8 µm
Maximum	72.0 µm
Average	17.6 µm
Standard deviation	11.4 µm
Skewness	0.0
Standard deviation (n-1)	11.4 µm
Variance	129.4 µm²
Variance (n-1)	129.8 µm²
Sum	6'305.7 µm

Statistical Data	Length
Sum of squares	157'394.0 μm²
Sum of cubes	5'075'679.4 μm³

2.1.1. Chord Lenght Distribution

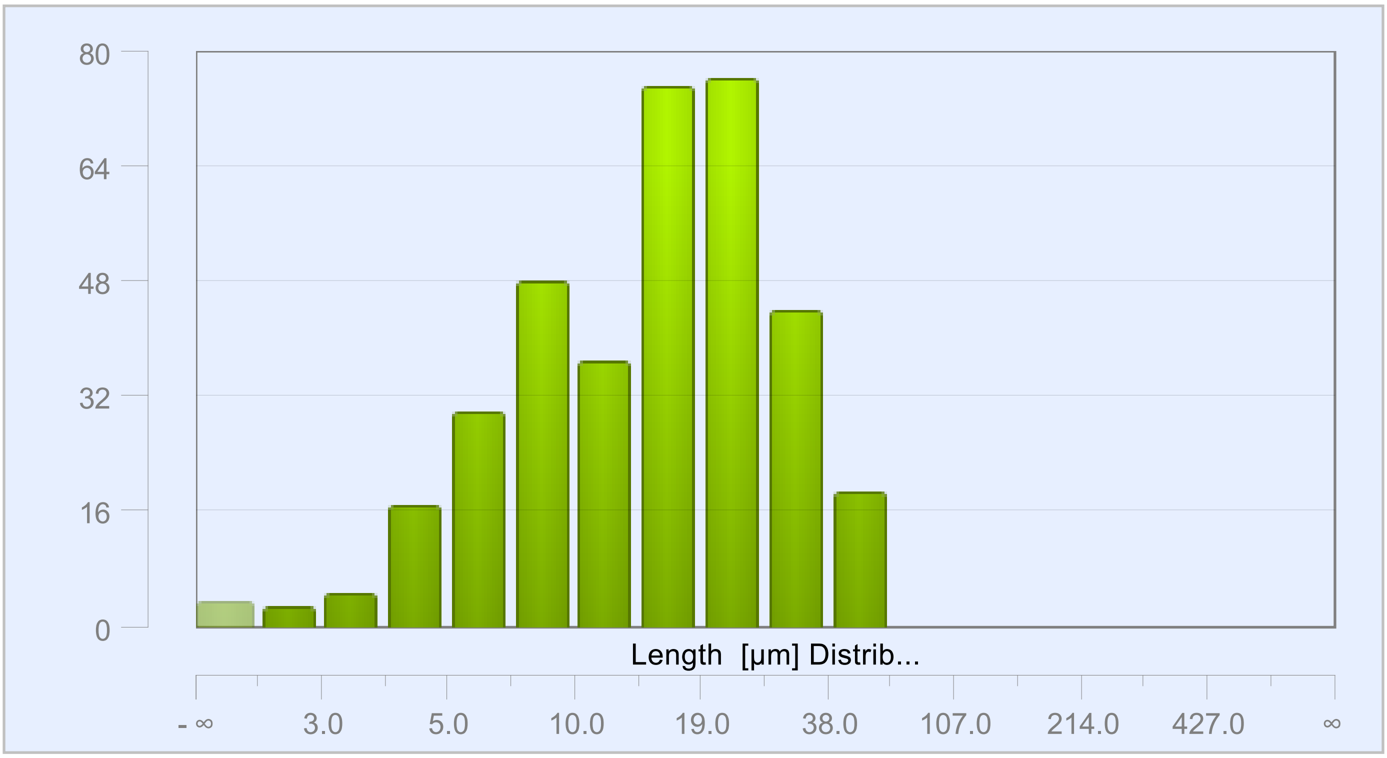

Start	End	Absolute Frequency	Absolute Frequency (accumulated)	Relative Frequency [%]	Relative Frequency (accumulated) [%]
	2.0 μm	4	4	1	1
2.0 μm	3.0 μm	3	7	1	2
3.0 μm	4.0 μm	5	12	1	3
4.0 μm	5.0 μm	17	29	5	8
5.0 μm	7.0 μm	30	59	8	16
7.0 μm	10.0 μm	48	107	13	30
10.0 μm	13.0 μm	37	144	10	40
13.0 μm	19.0 μm	75	219	21	61
19.0 μm	27.0 μm	76	295	21	82
27.0 μm	38.0 μm	44	339	12	95
38.0 μm	75.0 μm	19	358	5	100
75.0 μm	107.0 μm	0	358	0	100
107.0 μm	151.0 μm	0	358	0	100
151.0 μm	214.0 μm	0	358	0	100
214.0 μm	302.0 μm	0	358	0	100
302.0 μm	427.0 μm	0	358	0	100
427.0 μm	600.0 μm	0	358	0	100
600.0 μm		0	358	0	100

3. Single Result 2 (MnFeNi Semesterprojekt_MnFeNi_homogenized_8.1mmSW_800°C_60min_00057)

Mean chord length	19.9 μm
Grain size (ASTM)	8
Grain size (G643)	8
Grain stretching	91.8 %

3.1. Statistical Analysis

Statistical Data	Length
Object Count	316
Minimum	1.9 µm
Maximum	96.7 µm
Average	19.9 µm
Standard deviation	13.3 µm
Skewness	0.0
Standard deviation (n-1)	13.3 µm
Variance	177.3 µm²
Variance (n-1)	177.9 µm²
Sum	6'300.0 µm
Sum of squares	181'627.1 µm²
Sum of cubes	7'074'575.1 µm³

3.1.1. Chord Lenght Distribution

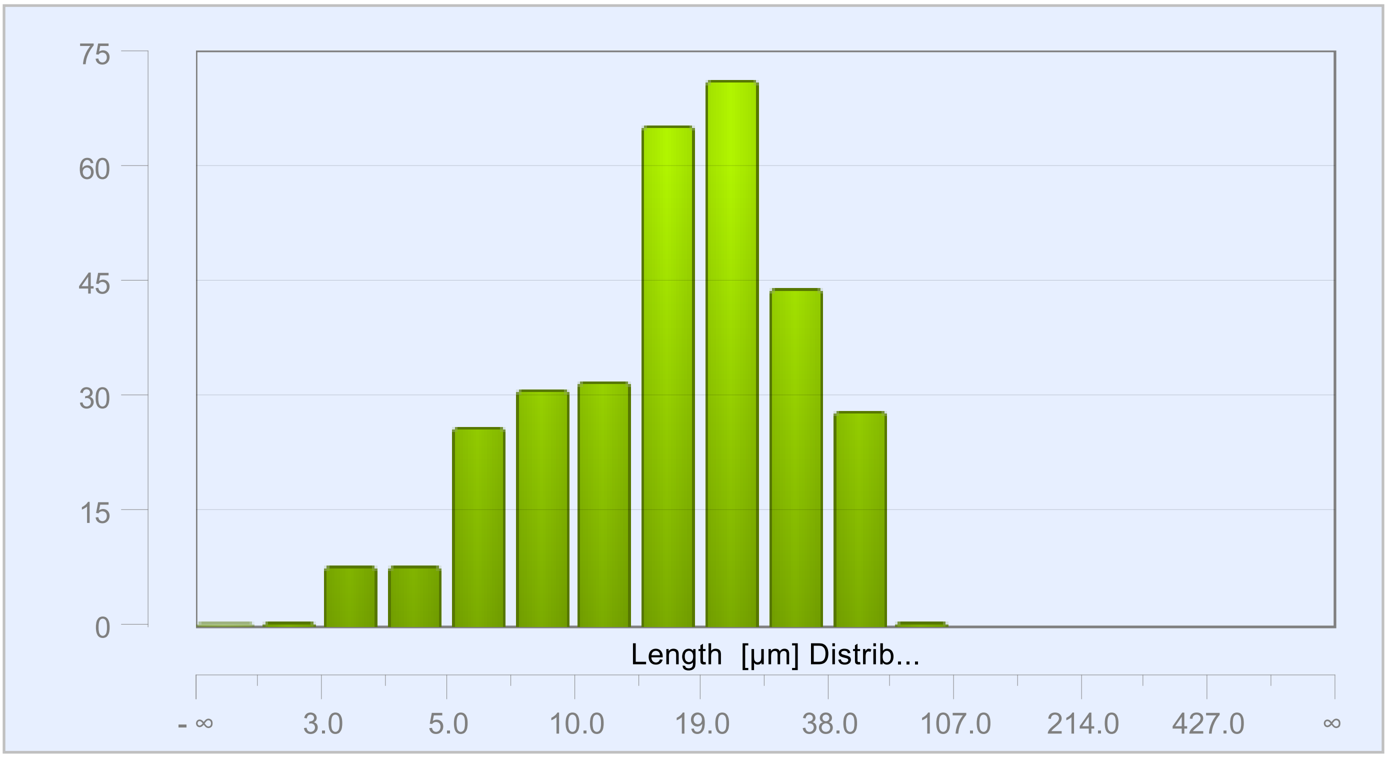

Start	End	Absolute Frequency	Absolute Frequency (accumulated)	Relative Frequency [%]	Relative Frequency (accumulated) [%]
	2.0 µm	1	1	0	0
2.0 µm	3.0 µm	1	2	0	1
3.0 µm	4.0 µm	8	10	3	3
4.0 µm	5.0 µm	8	18	3	6
5.0 µm	7.0 µm	26	44	8	14
7.0 µm	10.0 µm	31	75	10	24
10.0 µm	13.0 µm	32	107	10	34
13.0 µm	19.0 µm	65	172	21	54
19.0 µm	27.0 µm	71	243	22	77
27.0 µm	38.0 µm	44	287	14	91
38.0 µm	75.0 µm	28	315	9	100
75.0 µm	107.0 µm	1	316	0	100

Start	End	Absolute Frequency	Absolute Frequency (accumulated)	Relative Frequency [%]	Relative Frequency (accumulated) [%]
107.0 µm	151.0 µm	0	316	0	100
151.0 µm	214.0 µm	0	316	0	100
214.0 µm	302.0 µm	0	316	0	100
302.0 µm	427.0 µm	0	316	0	100
427.0 µm	600.0 µm	0	316	0	100
600.0 µm		0	316	0	100

4. Single Result 3 (MnFeNi Semesterprojekt_MnFeNi_homogenized_8.1mmSW_800°C_60min_00058)

Mean chord length	17.9 µm
Grain size (ASTM)	8.3
Grain size (G643)	8.3
Grain stretching	91.9 %

4.1. Statistical Analysis

Statistical Data	Length
Object Count	351
Minimum	1.2 µm
Maximum	69.5 µm
Average	17.9 µm
Standard deviation	12.7 µm
Skewness	0.0
Standard deviation (n-1)	12.7 µm
Variance	160.2 µm²
Variance (n-1)	160.7 µm²
Sum	6'299.9 µm
Sum of squares	169'310.9 µm²
Sum of cubes	6'082'510.8 µm³

4.1.1. Chord Lenght Distribution

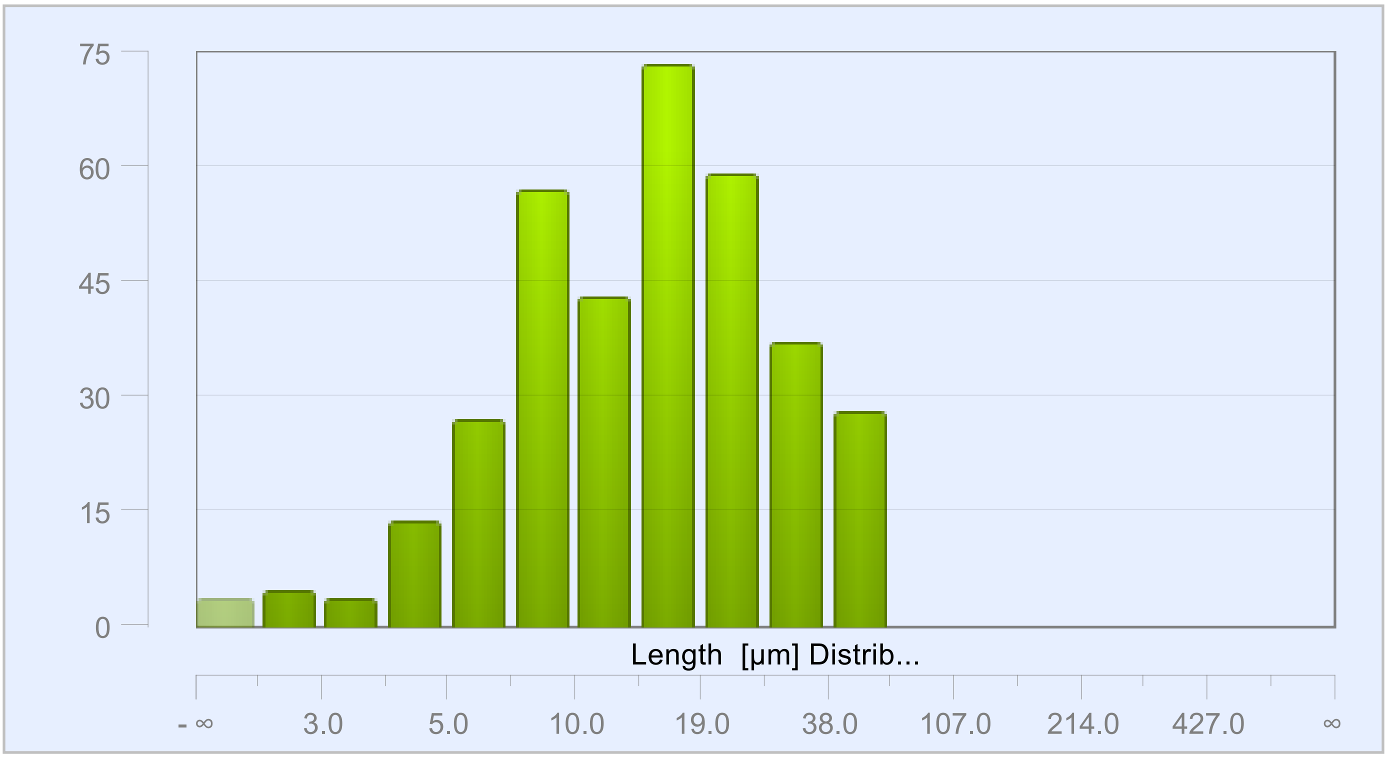

Start	End	Absolute Frequency	Absolute Frequency (accumulated)	Relative Frequency [%]	Relative Frequency (accumulated) [%]
	2.0 µm	4	4	1	1
2.0 µm	3.0 µm	5	9	1	3
3.0 µm	4.0 µm	4	13	1	4
4.0 µm	5.0 µm	14	27	4	8
5.0 µm	7.0 µm	27	54	8	15
7.0 µm	10.0 µm	57	111	16	32
10.0 µm	13.0 µm	43	154	12	44
13.0 µm	19.0 µm	73	227	21	65
19.0 µm	27.0 µm	59	286	17	81
27.0 µm	38.0 µm	37	323	11	92
38.0 µm	75.0 µm	28	351	8	100
75.0 µm	107.0 µm	0	351	0	100
107.0 µm	151.0 µm	0	351	0	100
151.0 µm	214.0 µm	0	351	0	100
214.0 µm	302.0 µm	0	351	0	100
302.0 µm	427.0 µm	0	351	0	100
427.0 µm	600.0 µm	0	351	0	100
600.0 µm		0	351	0	100

5. Single Result 4 (MnFeNi Semesterprojekt_MnFeNi_homogenized_8.1mmSW_800°C_60min_00059)

Mean chord length	18.6 µm
Grain size (ASTM)	8.2
Grain size (G643)	8.2
Grain stretching	88.5 %

5.1. Statistical Analysis

Statistical Data	Length
Object Count	339
Minimum	1.7 µm
Maximum	87.6 µm
Average	18.6 µm
Standard deviation	12.8 µm
Skewness	0.0
Standard deviation (n-1)	12.9 µm
Variance	164.7 µm²
Variance (n-1)	165.2 µm²
Sum	6'295.8 µm
Sum of squares	172'753.5 µm²
Sum of cubes	6'332'483.3 µm³

5.1.1. Chord Lenght Distribution

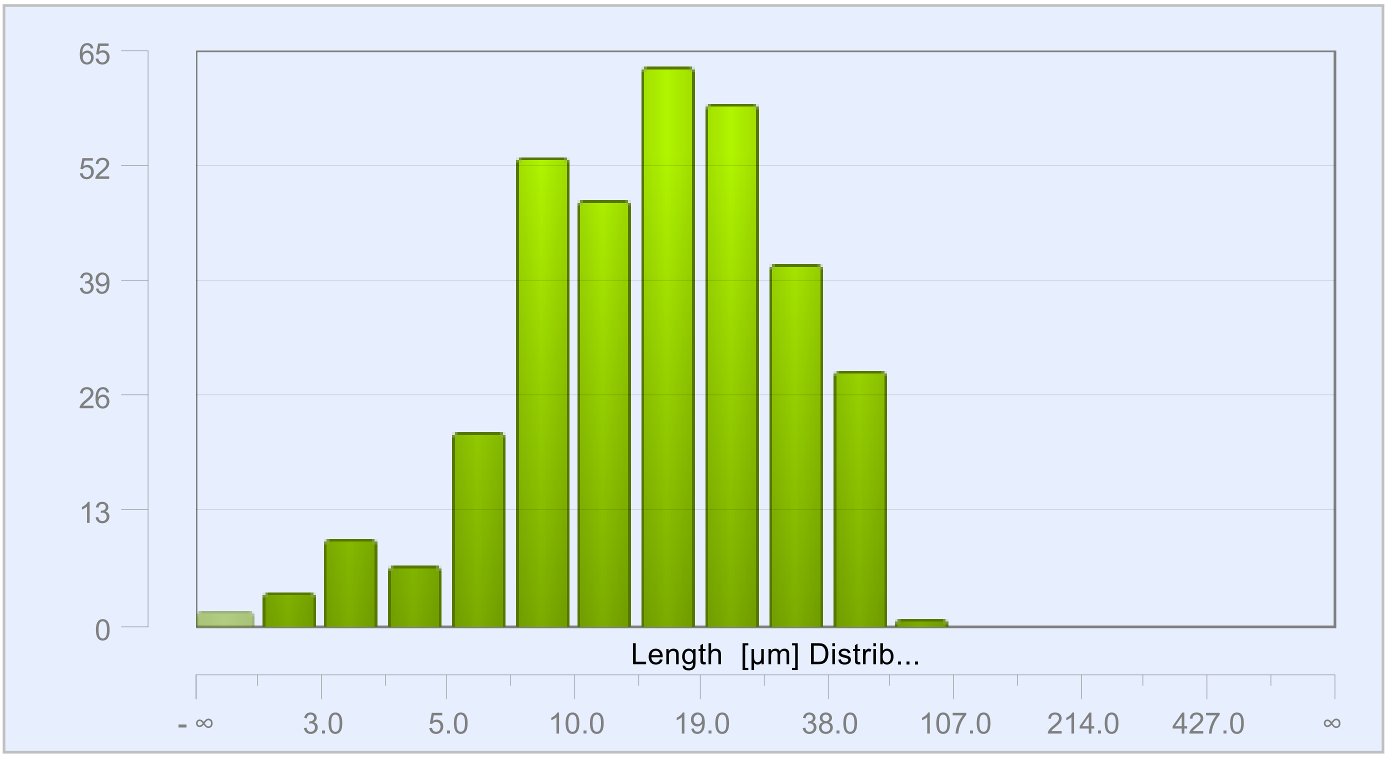

Start	End	Absolute Frequency	Absolute Frequency (accumulated)	Relative Frequency [%]	Relative Frequency (accumulated) [%]
	2.0 µm	2	2	1	1
2.0 µm	3.0 µm	4	6	1	2
3.0 µm	4.0 µm	10	16	3	5
4.0 µm	5.0 µm	7	23	2	7
5.0 µm	7.0 µm	22	45	6	13
7.0 µm	10.0 µm	53	98	16	29
10.0 µm	13.0 µm	48	146	14	43
13.0 µm	19.0 µm	63	209	19	62
19.0 µm	27.0 µm	59	268	17	79
27.0 µm	38.0 µm	41	309	12	91
38.0 µm	75.0 µm	29	338	9	100
75.0 µm	107.0 µm	1	339	0	100
107.0 µm	151.0 µm	0	339	0	100
151.0 µm	214.0 µm	0	339	0	100
214.0 µm	302.0 µm	0	339	0	100
302.0 µm	427.0 µm	0	339	0	100
427.0 µm	600.0 µm	0	339	0	100
600.0 µm		0	339	0	100
