# Supplementary material for: Data compilation on the effect of grain size, temperature, and texture on the strength of a single-phase FCC MnFeNi medium-entropy alloy
Source: Data Brief. 2019 Nov 15;28:104807. doi: 10.1016/j.dib.2019.104807 (PMC6909151; doi:10.1016/j.dib.2019.104807)
Supplement: Multimedia component 1 [file mmc1.zip › MnFeNi_1173K_30min/MnFeNi_1173K_30min_c=18μm.pdf]

# Analysebericht

02.10.2019 16:22:31

powered by imagic.ch

## 1. cumulative Result 1

| | |
|---|---|
| Anzahl Bilder | 4 |
| Korngröße (ASTM) | 8,3 |
| Korngröße (G643) | 8,3 |
| Kornstreckung | 98,1 % |
| Mittlere Sehnenlänge | 17,7 µm |

## 2. Single Result 1 (MnFeNi Semesterprojekt_MnFeNi_homogenized_8.1mmSW_900°C_30min_00133)

| | |
|---|---|
| Mittlere Sehnenlänge | 17,9 µm |
| Korngröße (ASTM) | 8,3 |
| Korngröße (G643) | 8,3 |
| Kornstreckung | 96,2 % |

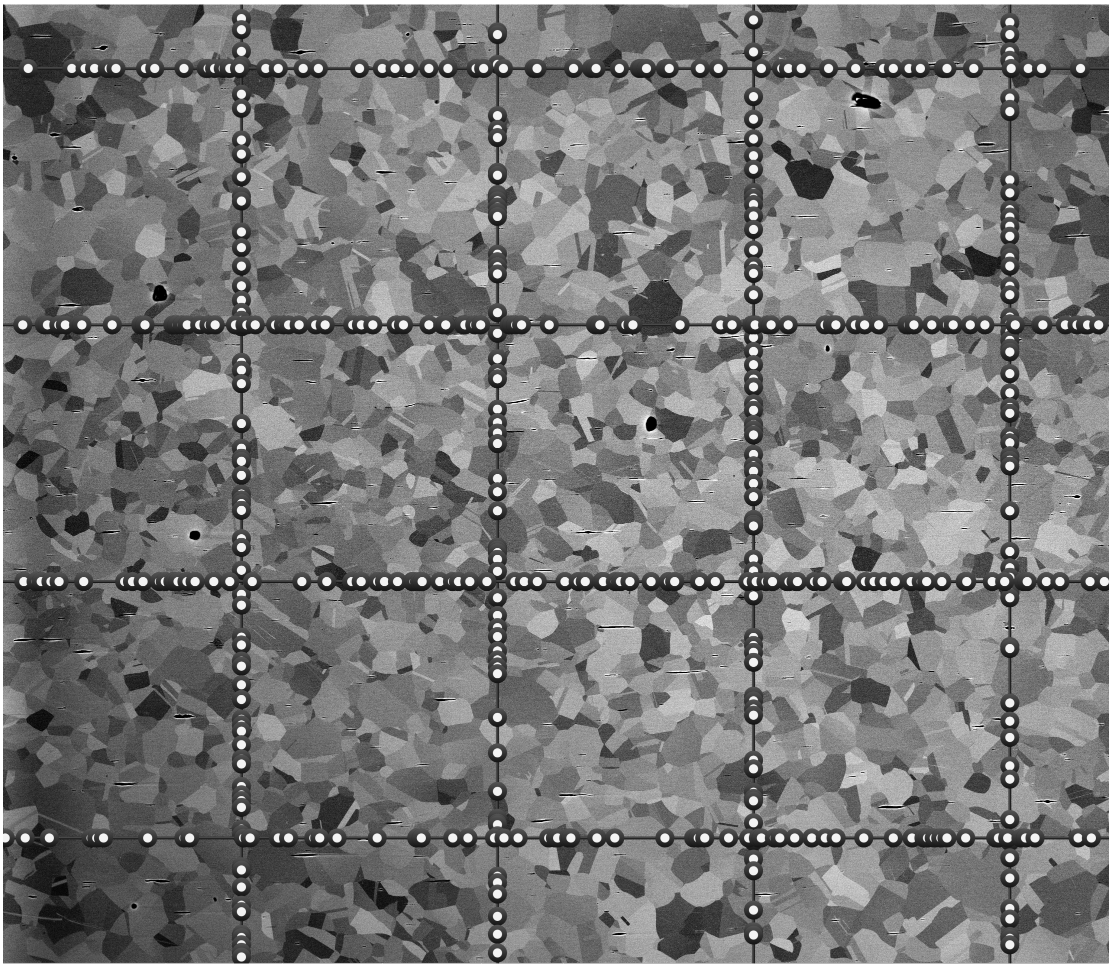

### 2.1. Statistische Analyse

| Statistische Daten | Länge |
|---|---|
| Anzahl Objekte | 528 |
| Minimum | 0,9 µm |
| Maximum | 78,1 µm |
| Mittelwert | 17,9 µm |
| Standardabweichung | 13,0 µm |
| Schiefe | 0,0 |
| Standardabweichung (n-1) | 13,0 µm |
| Varianz | 169,3 µm² |
| Varianz (n-1) | 169,6 µm² |
| Summe | 9'448,1 µm |

| Statistische Daten | Länge |
|---|---|
| Quadratsumme | 258'431,9 µm² |
| Kubiksumme | 9'158'385,1 µm³ |

### 2.1.1. Chord Lenght Distribution

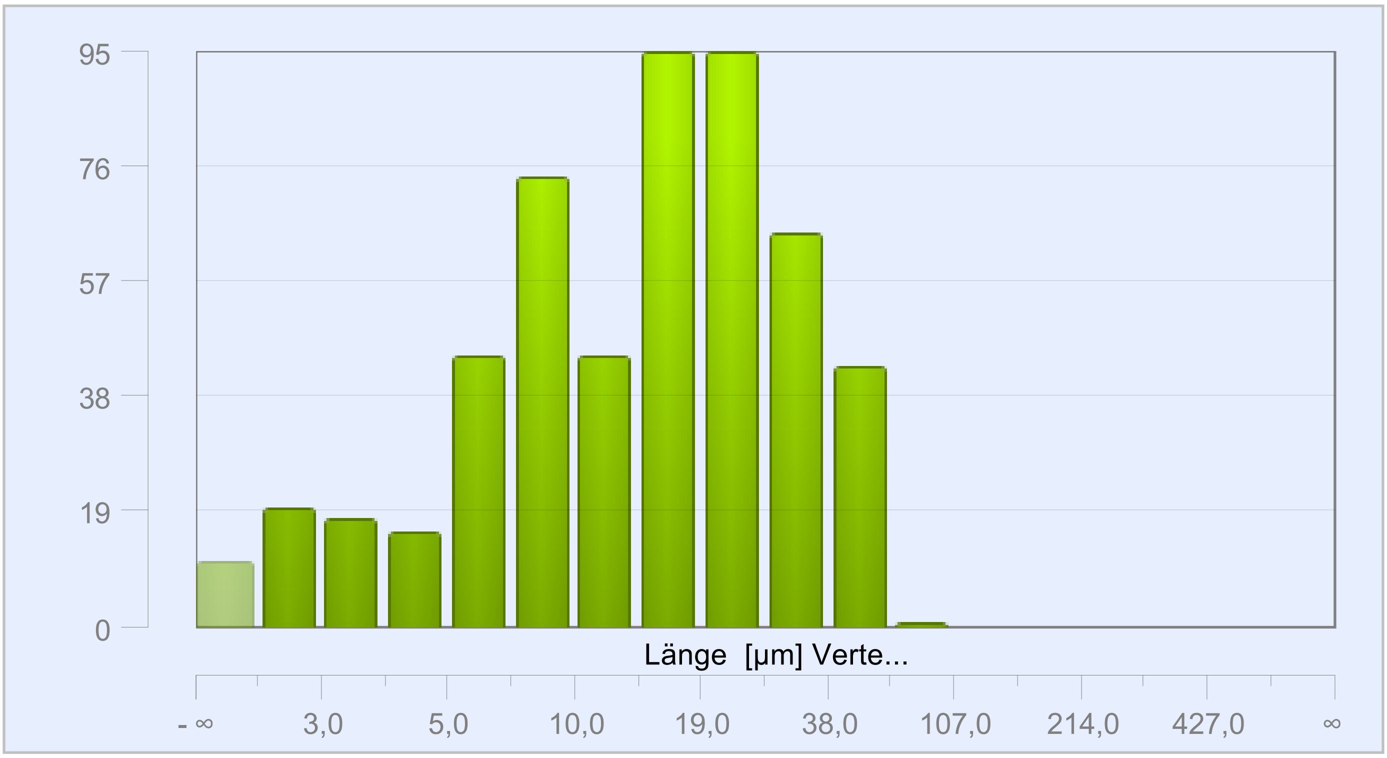


| Start | Ende | Absolute Häufigkeit | Absolute Häufigkeit (kumuliert) | Relative Häufigkeit [%] | Relative Häufigkeit (kumuliert) [%] |
|---|---|---|---|---|---|
| | 2,0 µm | 11 | 11 | 2 | 2 |
| 2,0 µm | 3,0 µm | 20 | 31 | 4 | 6 |
| 3,0 µm | 4,0 µm | 18 | 49 | 3 | 9 |
| 4,0 µm | 5,0 µm | 16 | 65 | 3 | 12 |
| 5,0 µm | 7,0 µm | 45 | 110 | 9 | 21 |
| 7,0 µm | 10,0 µm | 74 | 184 | 14 | 35 |
| 10,0 µm | 13,0 µm | 45 | 229 | 9 | 43 |
| 13,0 µm | 19,0 µm | 95 | 324 | 18 | 61 |
| 19,0 µm | 27,0 µm | 95 | 419 | 18 | 79 |
| 27,0 µm | 38,0 µm | 65 | 484 | 12 | 92 |
| 38,0 µm | 75,0 µm | 43 | 527 | 8 | 100 |
| 75,0 µm | 107,0 µm | 1 | 528 | 0 | 100 |
| 107,0 µm | 151,0 µm | 0 | 528 | 0 | 100 |
| 151,0 µm | 214,0 µm | 0 | 528 | 0 | 100 |
| 214,0 µm | 302,0 µm | 0 | 528 | 0 | 100 |
| 302,0 µm | 427,0 µm | 0 | 528 | 0 | 100 |
| 427,0 µm | 600,0 µm | 0 | 528 | 0 | 100 |
| 600,0 µm | | 0 | 528 | 0 | 100 |

## 3. Single Result 2 (MnFeNi Semesterprojekt_MnFeNi_homogenized_8.1mmSW_900°C_30min_00134)

| | |
|---|---|
| Mittlere Sehnenlänge | 18,2 µm |
| Korngröße (ASTM) | 8,3 |
| Korngröße (G643) | 8,2 |
| Kornstreckung | 95,5 % |

## 3.1. Statistische Analyse

| Statistische Daten | Länge |
|---|---|
| Anzahl Objekte | 518 |
| Minimum | 0,9 µm |
| Maximum | 71,9 µm |
| Mittelwert | 18,2 µm |
| Standardabweichung | 12,4 µm |
| Schiefe | 0,0 |
| Standardabweichung (n-1) | 12,4 µm |
| Varianz | 152,6 µm² |
| Varianz (n-1) | 152,9 µm² |
| Summe | 9'448,1 µm |
| Quadratsumme | 251'361,4 µm² |
| Kubiksumme | 8'430'264,3 µm³ |

### 3.1.1. Chord Lenght Distribution

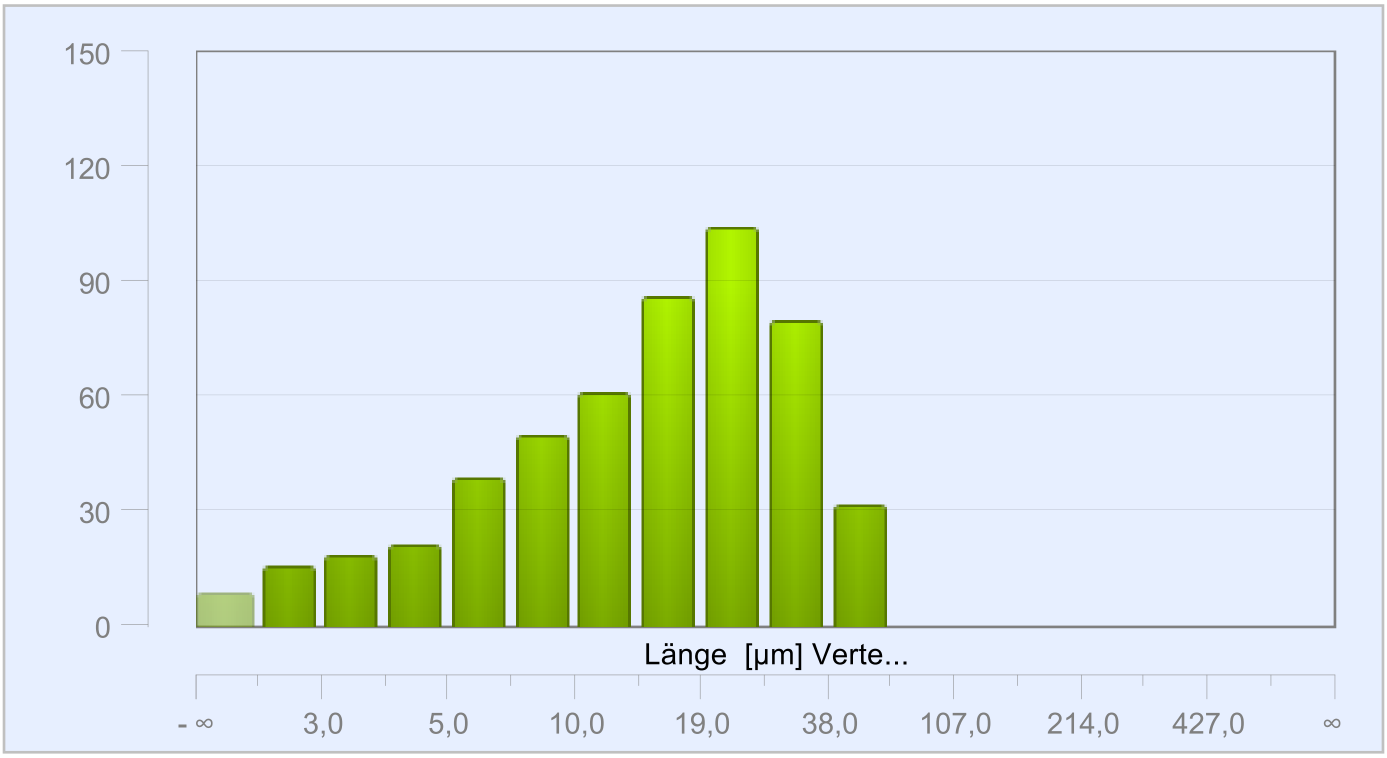


| Start | Ende | Absolute Häufigkeit | Absolute Häufigkeit (kumuliert) | Relative Häufigkeit [%] | Relative Häufigkeit (kumuliert) [%] |
|---|---|---|---|---|---|
| | 2,0 µm | 9 | 9 | 2 | 2 |
| 2,0 µm | 3,0 µm | 16 | 25 | 3 | 5 |
| 3,0 µm | 4,0 µm | 19 | 44 | 4 | 8 |
| 4,0 µm | 5,0 µm | 22 | 66 | 4 | 13 |
| 5,0 µm | 7,0 µm | 39 | 105 | 8 | 20 |
| 7,0 µm | 10,0 µm | 50 | 155 | 10 | 30 |
| 10,0 µm | 13,0 µm | 61 | 216 | 12 | 42 |
| 13,0 µm | 19,0 µm | 86 | 302 | 17 | 58 |
| 19,0 µm | 27,0 µm | 104 | 406 | 20 | 78 |
| 27,0 µm | 38,0 µm | 80 | 486 | 15 | 94 |
| 38,0 µm | 75,0 µm | 32 | 518 | 6 | 100 |
| 75,0 µm | 107,0 µm | 0 | 518 | 0 | 100 |

| Start | Ende | Absolute Häufigkeit | Absolute Häufigkeit (kumuliert) | Relative Häufigkeit [%] | Relative Häufigkeit (kumuliert) [%] |
|---|---|---|---|---|---|
| 107,0 µm | 151,0 µm | 0 | 518 | 0 | 100 |
| 151,0 µm | 214,0 µm | 0 | 518 | 0 | 100 |
| 214,0 µm | 302,0 µm | 0 | 518 | 0 | 100 |
| 302,0 µm | 427,0 µm | 0 | 518 | 0 | 100 |
| 427,0 µm | 600,0 µm | 0 | 518 | 0 | 100 |
| 600,0 µm | | 0 | 518 | 0 | 100 |

## 4. Single Result 3 (MnFeNi Semesterprojekt_MnFeNi_homogenized_8.1mmSW_900°C_30min_00135)

| | |
|---|---|
| Mittlere Sehnenlänge | 17,9 µm |
| Korngröße (ASTM) | 8,3 |
| Korngröße (G643) | 8,3 |
| Kornstreckung | 95,2 % |

### 4.1. Statistische Analyse

| Statistische Daten | Länge |
|---|---|
| Anzahl Objekte | 530 |
| Minimum | 0,9 µm |
| Maximum | 93,0 µm |
| Mittelwert | 17,9 µm |
| Standardabweichung | 13,2 µm |
| Schiefe | 0,0 |
| Standardabweichung (n-1) | 13,3 µm |
| Varianz | 175,4 µm² |
| Varianz (n-1) | 175,7 µm² |
| Summe | 9'460,5 µm |
| Quadratsumme | 261'824,5 µm² |
| Kubiksumme | 9'738'865,6 µm³ |

#### 4.1.1. Chord Lenght Distribution

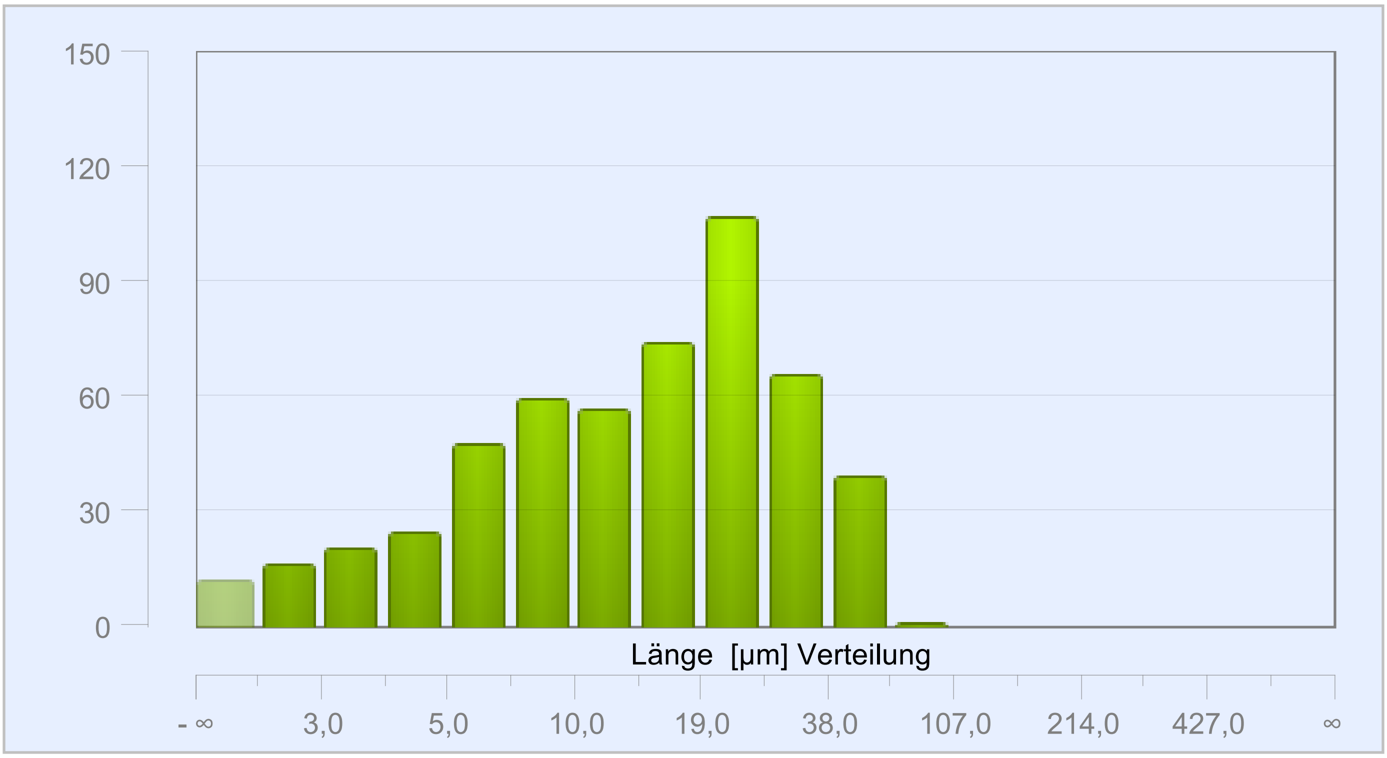

| Start | Ende | Absolute Häufigkeit | Absolute Häufigkeit (kumuliert) | Relative Häufigkeit [%] | Relative Häufigkeit (kumuliert) [%] |
|---|---|---|---|---|---|
| | 2,0 µm | 13 | 13 | 2 | 2 |
| 2,0 µm | 3,0 µm | 17 | 30 | 3 | 6 |
| 3,0 µm | 4,0 µm | 21 | 51 | 4 | 10 |
| 4,0 µm | 5,0 µm | 25 | 76 | 5 | 14 |
| 5,0 µm | 7,0 µm | 48 | 124 | 9 | 23 |
| 7,0 µm | 10,0 µm | 60 | 184 | 11 | 35 |
| 10,0 µm | 13,0 µm | 57 | 241 | 11 | 45 |
| 13,0 µm | 19,0 µm | 74 | 315 | 14 | 59 |
| 19,0 µm | 27,0 µm | 107 | 422 | 20 | 80 |
| 27,0 µm | 38,0 µm | 66 | 488 | 12 | 92 |
| 38,0 µm | 75,0 µm | 40 | 528 | 8 | 100 |
| 75,0 µm | 107,0 µm | 2 | 530 | 0 | 100 |
| 107,0 µm | 151,0 µm | 0 | 530 | 0 | 100 |
| 151,0 µm | 214,0 µm | 0 | 530 | 0 | 100 |
| 214,0 µm | 302,0 µm | 0 | 530 | 0 | 100 |
| 302,0 µm | 427,0 µm | 0 | 530 | 0 | 100 |
| 427,0 µm | 600,0 µm | 0 | 530 | 0 | 100 |
| 600,0 µm | | 0 | 530 | 0 | 100 |

## 5. Single Result 4 (MnFeNi Semesterprojekt_MnFeNi_homogenized_8.1mmSW_900°C_30min_00136)

| | |
|---|---|
| Mittlere Sehnenlänge | 17 µm |
| Korngröße (ASTM) | 8,5 |
| Korngröße (G643) | 8,4 |
| Kornstreckung | 96 % |

### 5.1. Statistische Analyse

| Statistische Daten | Länge |
|---|---|
| Anzahl Objekte | 558 |
| Minimum | 0,9 µm |
| Maximum | 74,1 µm |
| Mittelwert | 17,0 µm |
| Standardabweichung | 12,4 µm |
| Schiefe | 0,0 |
| Standardabweichung (n-1) | 12,4 µm |
| Varianz | 153,9 µm² |
| Varianz (n-1) | 154,2 µm² |
| Summe | 9'465,5 µm |
| Quadratsumme | 246'453,8 µm² |
| Kubiksumme | 8'459'305,4 µm³ |

#### 5.1.1. Chord Lenght Distribution

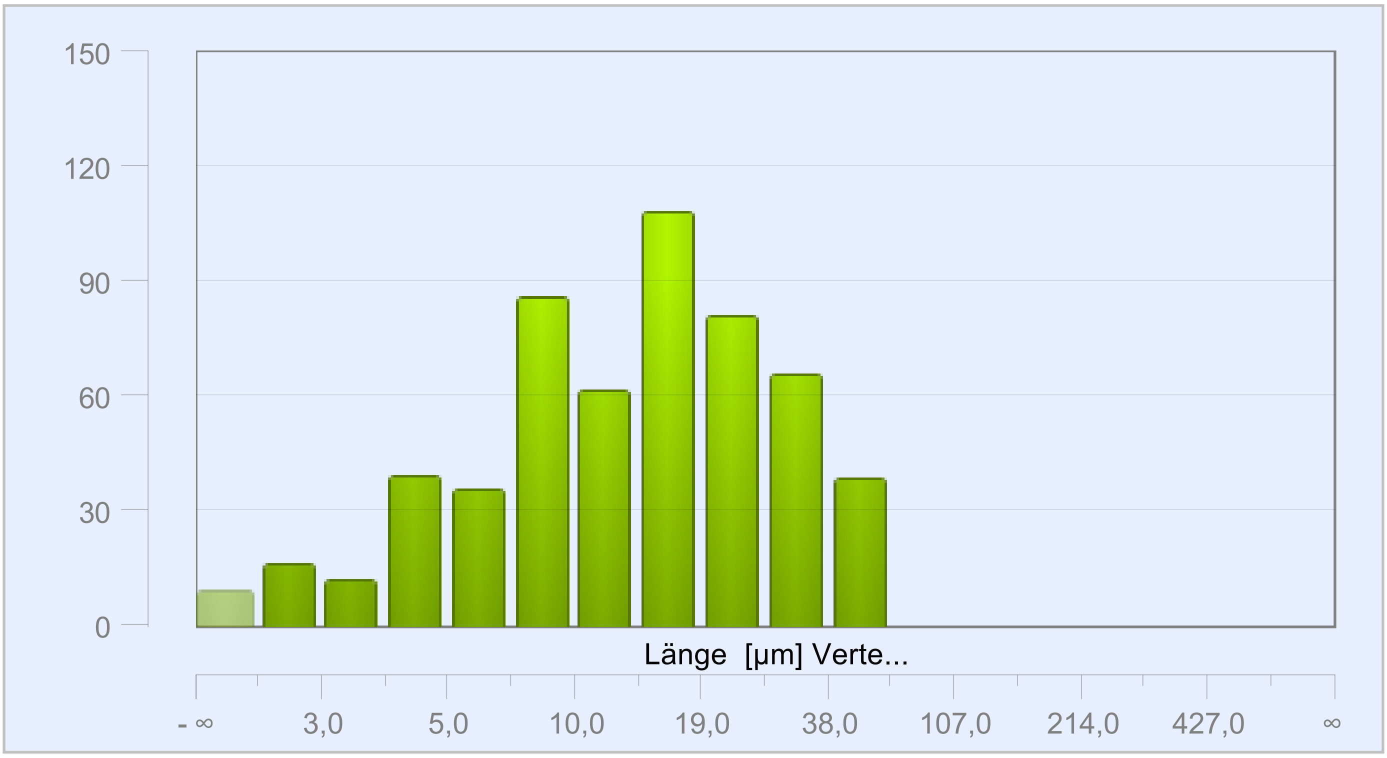


| Start | Ende | Absolute Häufigkeit | Absolute Häufigkeit (kumuliert) | Relative Häufigkeit [%] | Relative Häufigkeit (kumuliert) [%] |
|---|---|---|---|---|---|
| | 2,0 µm | 10 | 10 | 2 | 2 |
| 2,0 µm | 3,0 µm | 17 | 27 | 3 | 5 |
| 3,0 µm | 4,0 µm | 13 | 40 | 2 | 7 |
| 4,0 µm | 5,0 µm | 40 | 80 | 7 | 14 |
| 5,0 µm | 7,0 µm | 36 | 116 | 6 | 21 |
| 7,0 µm | 10,0 µm | 86 | 202 | 15 | 36 |
| 10,0 µm | 13,0 µm | 62 | 264 | 11 | 47 |
| 13,0 µm | 19,0 µm | 108 | 372 | 19 | 67 |
| 19,0 µm | 27,0 µm | 81 | 453 | 15 | 81 |
| 27,0 µm | 38,0 µm | 66 | 519 | 12 | 93 |
| 38,0 µm | 75,0 µm | 39 | 558 | 7 | 100 |
| 75,0 µm | 107,0 µm | 0 | 558 | 0 | 100 |
| 107,0 µm | 151,0 µm | 0 | 558 | 0 | 100 |
| 151,0 µm | 214,0 µm | 0 | 558 | 0 | 100 |
| 214,0 µm | 302,0 µm | 0 | 558 | 0 | 100 |
| 302,0 µm | 427,0 µm | 0 | 558 | 0 | 100 |
| 427,0 µm | 600,0 µm | 0 | 558 | 0 | 100 |
| 600,0 µm | | 0 | 558 | 0 | 100 |
