# Supplementary material for: Data compilation on the effect of grain size, temperature, and texture on the strength of a single-phase FCC MnFeNi medium-entropy alloy
Source: Data Brief. 2019 Nov 15;28:104807. doi: 10.1016/j.dib.2019.104807 (PMC6909151; doi:10.1016/j.dib.2019.104807)
Supplement: Multimedia component 1 [file mmc1.zip › MnFeNi_1173K_30min/MnFeNi_1173K_30min_d=30μm.pdf]

Analysebericht

02.10.2019 14:06:27

powered by imagic.ch

1. cumulative Result 1

Anzahl Bilder	4
Korngröße (ASTM)	6,8
Korngröße (G643)	6,8
Kornstreckung	93,3 %
Mittlere Sehnenlänge	30,2 µm

2. Single Result 1 (MnFeNi Semesterprojekt_MnFeNi_homogenized_8.1mmSW_900°C_30min_00133)

Mittlere Sehnenlänge	30,7 µm
Korngröße (ASTM)	6,8
Korngröße (G643)	6,7
Kornstreckung	96,2 %

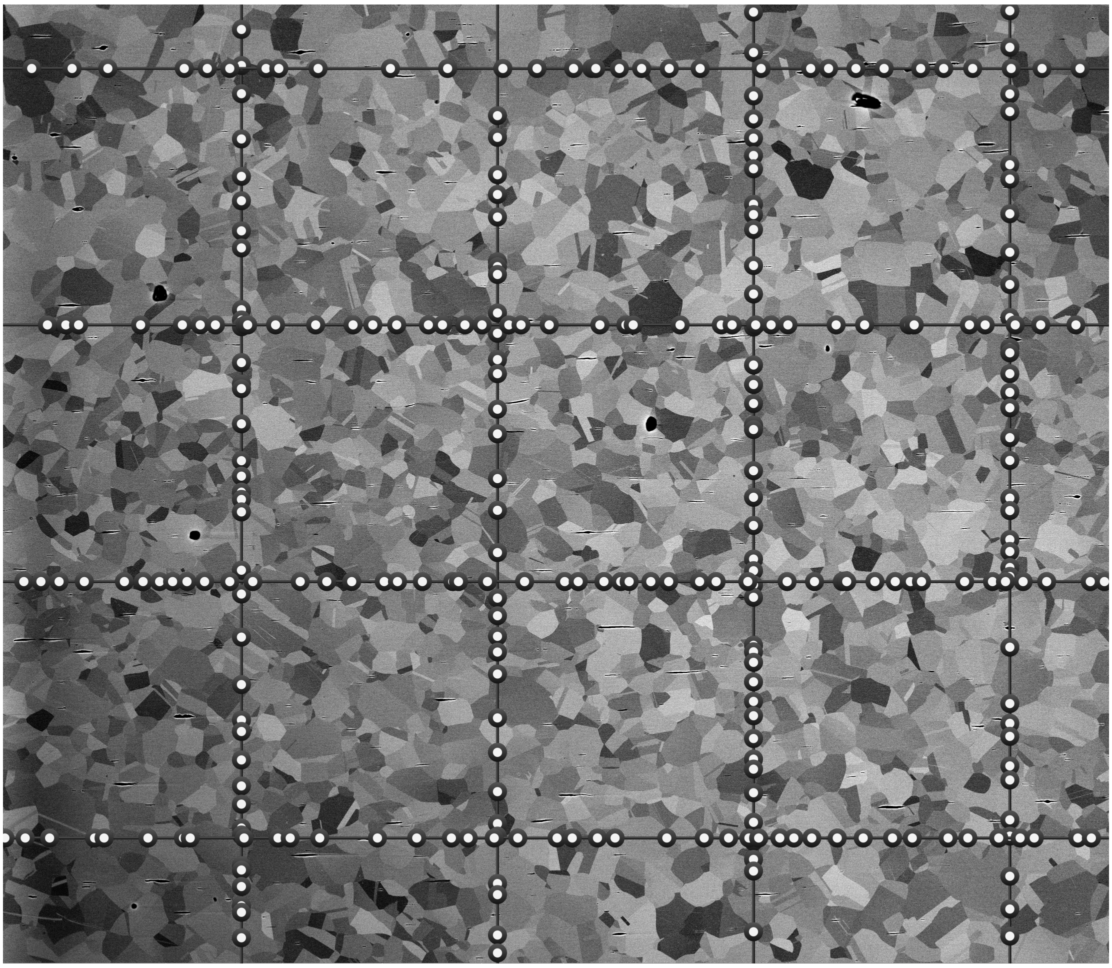

2.1. Statistische Analyse

Statistische Daten	Länge
Anzahl Objekte	308
Minimum	2,2 µm
Maximum	127,1 µm
Mittelwert	30,7 µm
Standardabweichung	16,6 µm
Schiefe	0,0
Standardabweichung (n-1)	16,6 µm
Varianz	276,0 µm²
Varianz (n-1)	276,9 µm²
Summe	9'448,1 µm

Statistische Daten	Länge
Quadratsumme	374'824,4 µm²
Kubiksumme	18'387'040,6 µm³

2.1.1. Chord Lenght Distribution

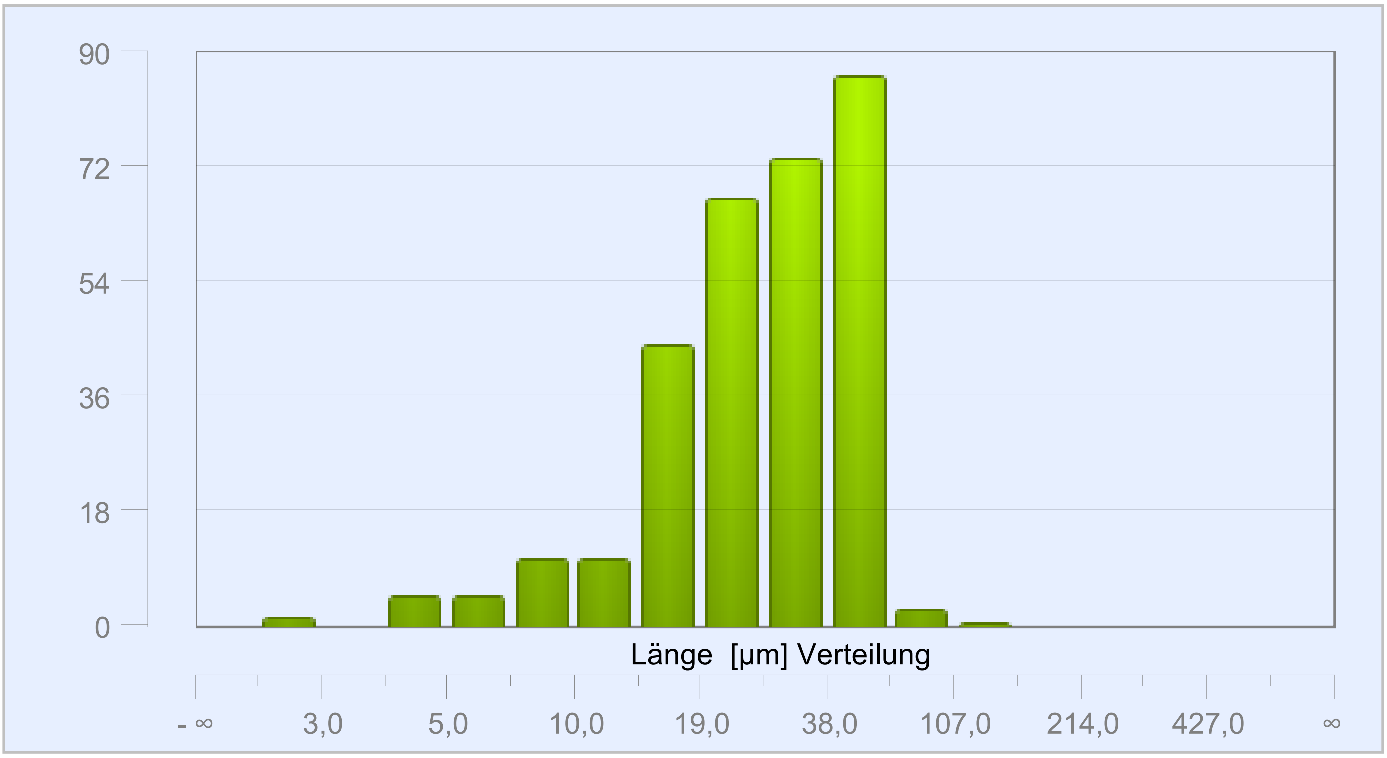

Start	Ende	Absolute Häufigkeit	Absolute Häufigkeit (kumuliert)	Relative Häufigkeit [%]	Relative Häufigkeit (kumuliert) [%]
	2,0 µm	0	0	0	0
2,0 µm	3,0 µm	2	2	1	1
3,0 µm	4,0 µm	0	2	0	1
4,0 µm	5,0 µm	5	7	2	2
5,0 µm	7,0 µm	5	12	2	4
7,0 µm	10,0 µm	11	23	4	7
10,0 µm	13,0 µm	11	34	4	11
13,0 µm	19,0 µm	44	78	14	25
19,0 µm	27,0 µm	67	145	22	47
27,0 µm	38,0 µm	73	218	24	71
38,0 µm	75,0 µm	86	304	28	99
75,0 µm	107,0 µm	3	307	1	100
107,0 µm	151,0 µm	1	308	0	100
151,0 µm	214,0 µm	0	308	0	100
214,0 µm	302,0 µm	0	308	0	100
302,0 µm	427,0 µm	0	308	0	100
427,0 µm	600,0 µm	0	308	0	100
600,0 µm		0	308	0	100

3. Single Result 2 (MnFeNi Semesterprojekt_MnFeNi_homogenized_8.1mmSW_900°C_30min_00134)

Mittlere Sehnenlänge	31,4 µm
Korngröße (ASTM)	6,7
Korngröße (G643)	6,7
Kornstreckung	94 %

3.1. Statistische Analyse

Statistische Daten	Länge
Anzahl Objekte	301
Minimum	1,2 µm
Maximum	85,6 µm
Mittelwert	31,4 µm
Standardabweichung	15,4 µm
Schiefe	0,0
Standardabweichung (n-1)	15,5 µm
Varianz	238,3 µm²
Varianz (n-1)	239,1 µm²
Summe	9'448,1 µm
Quadratsumme	368'291,6 µm²
Kubiksumme	16'977'924,2 µm³

3.1.1. Chord Lenght Distribution

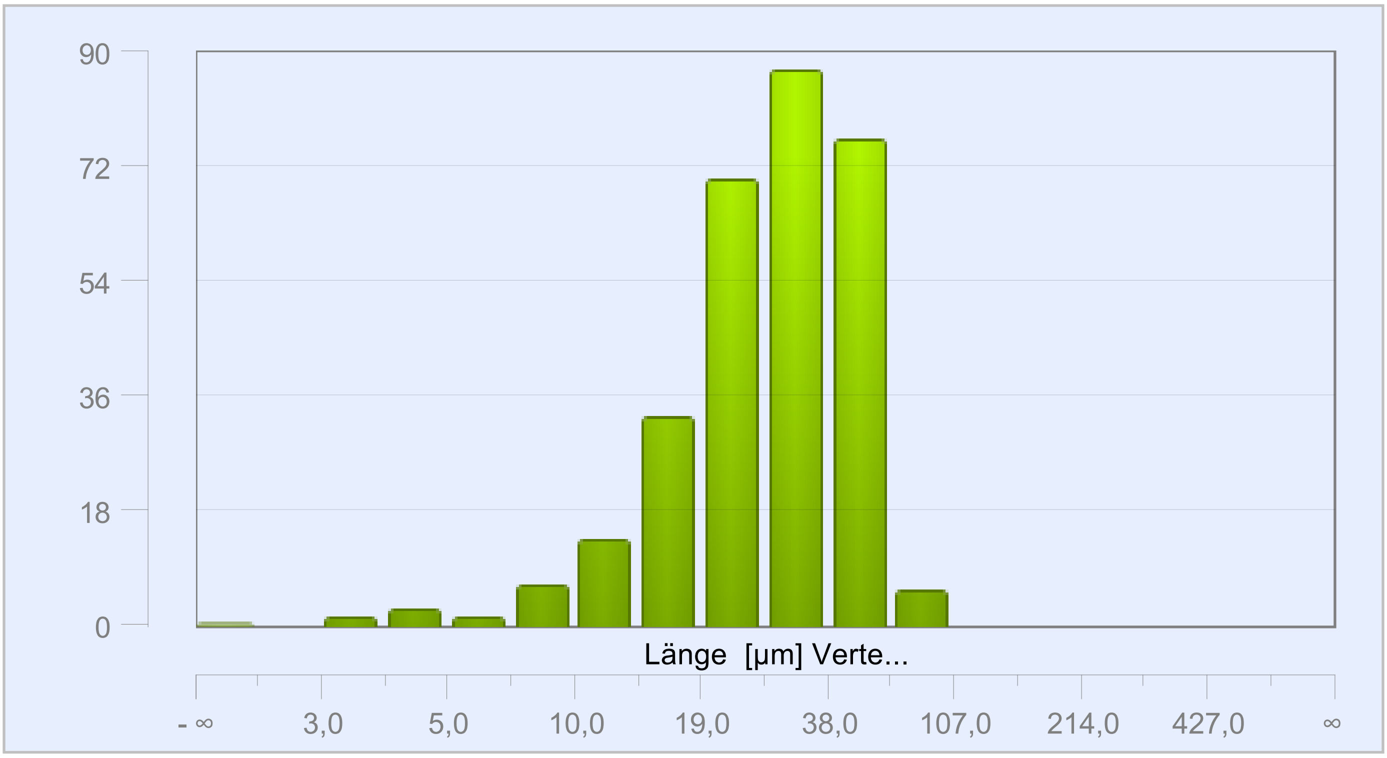

Start	Ende	Absolute Häufigkeit	Absolute Häufigkeit (kumuliert)	Relative Häufigkeit [%]	Relative Häufigkeit (kumuliert) [%]
	2,0 µm	1	1	0	0
2,0 µm	3,0 µm	0	1	0	0
3,0 µm	4,0 µm	2	3	1	1
4,0 µm	5,0 µm	3	6	1	2
5,0 µm	7,0 µm	2	8	1	3
7,0 µm	10,0 µm	7	15	2	5
10,0 µm	13,0 µm	14	29	5	10
13,0 µm	19,0 µm	33	62	11	21
19,0 µm	27,0 µm	70	132	23	44
27,0 µm	38,0 µm	87	219	29	73
38,0 µm	75,0 µm	76	295	25	98
75,0 µm	107,0 µm	6	301	2	100

Start	Ende	Absolute Häufigkeit	Absolute Häufigkeit (kumuliert)	Relative Häufigkeit [%]	Relative Häufigkeit (kumuliert) [%]
107,0 µm	151,0 µm	0	301	0	100
151,0 µm	214,0 µm	0	301	0	100
214,0 µm	302,0 µm	0	301	0	100
302,0 µm	427,0 µm	0	301	0	100
427,0 µm	600,0 µm	0	301	0	100
600,0 µm		0	301	0	100

4. Single Result 3 (MnFeNi Semesterprojekt_MnFeNi_homogenized_8.1mmSW_900°C_30min_00135)

Mittlere Sehnenlänge	28,8 µm
Korngröße (ASTM)	7
Korngröße (G643)	6,9
Kornstreckung	87,9 %

4.1. Statistische Analyse

Statistische Daten	Länge
Anzahl Objekte	329
Minimum	4,0 µm
Maximum	70,7 µm
Mittelwert	28,8 µm
Standardabweichung	14,5 µm
Schiefe	0,0
Standardabweichung (n-1)	14,6 µm
Varianz	211,2 µm²
Varianz (n-1)	211,8 µm²
Summe	9'460,5 µm
Quadratsumme	341'517,1 µm²
Kubiksumme	14'222'844,1 µm³

4.1.1. Chord Lenght Distribution

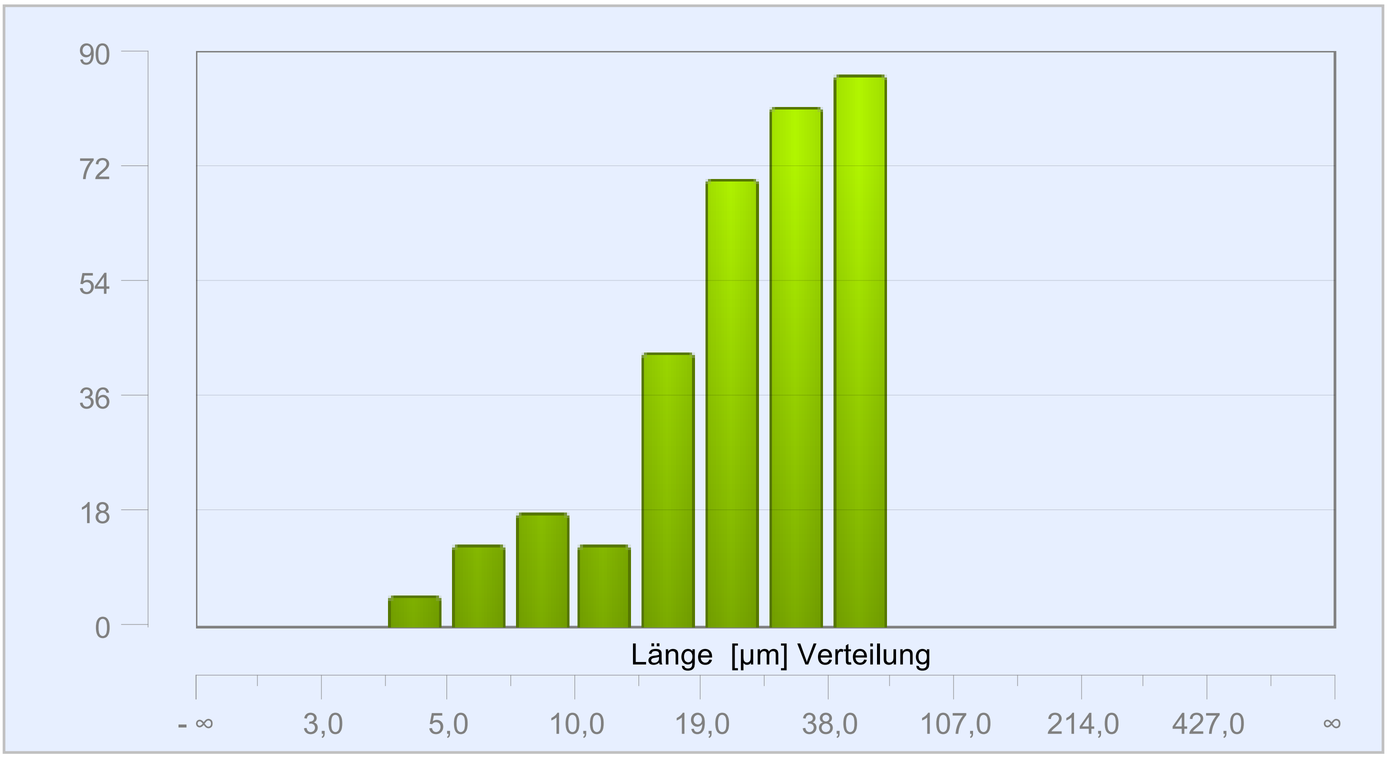

Start	Ende	Absolute Häufigkeit	Absolute Häufigkeit (kumuliert)	Relative Häufigkeit [%]	Relative Häufigkeit (kumuliert) [%]
	2,0 µm	0	0	0	0
2,0 µm	3,0 µm	0	0	0	0
3,0 µm	4,0 µm	0	0	0	0
4,0 µm	5,0 µm	5	5	2	2
5,0 µm	7,0 µm	13	18	4	5
7,0 µm	10,0 µm	18	36	5	11
10,0 µm	13,0 µm	13	49	4	15
13,0 µm	19,0 µm	43	92	13	28
19,0 µm	27,0 µm	70	162	21	49
27,0 µm	38,0 µm	81	243	25	74
38,0 µm	75,0 µm	86	329	26	100
75,0 µm	107,0 µm	0	329	0	100
107,0 µm	151,0 µm	0	329	0	100
151,0 µm	214,0 µm	0	329	0	100
214,0 µm	302,0 µm	0	329	0	100
302,0 µm	427,0 µm	0	329	0	100
427,0 µm	600,0 µm	0	329	0	100
600,0 µm		0	329	0	100

5. Single Result 4 (MnFeNi Semesterprojekt_MnFeNi_homogenized_8.1mmSW_900°C_30min_00136)

Mittlere Sehnenlänge	30,1 µm
Korngröße (ASTM)	6,8
Korngröße (G643)	6,8
Kornstreckung	95,6 %

5.1. Statistische Analyse

Statistische Daten	Länge
Anzahl Objekte	314
Minimum	4,0 µm
Maximum	88,4 µm
Mittelwert	30,1 µm
Standardabweichung	15,2 µm
Schiefe	0,0
Standardabweichung (n-1)	15,2 µm
Varianz	230,5 µm²
Varianz (n-1)	231,3 µm²
Summe	9'465,5 µm
Quadratsumme	357'725,0 µm²
Kubiksumme	15'731'837,1 µm³

5.1.1. Chord Lenght Distribution

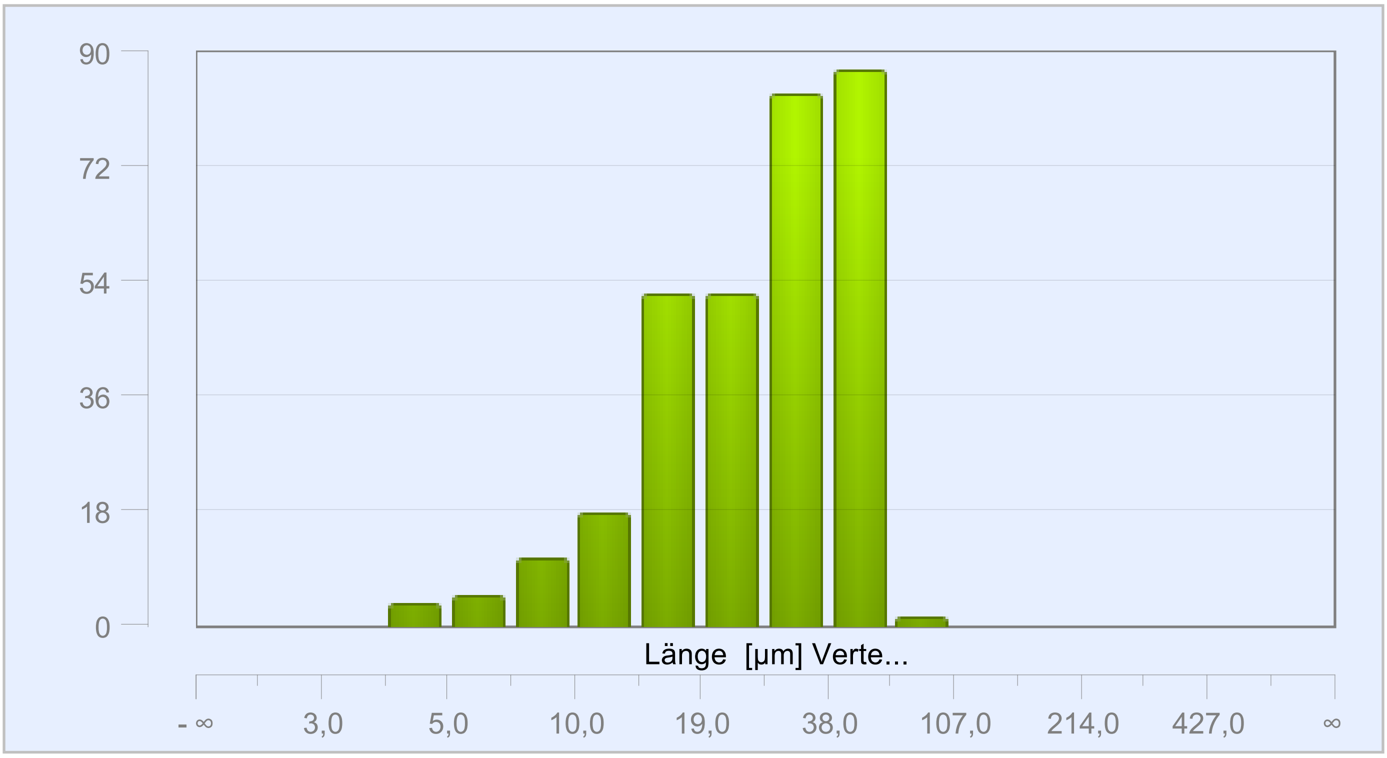

Start	Ende	Absolute Häufigkeit	Absolute Häufigkeit (kumuliert)	Relative Häufigkeit [%]	Relative Häufigkeit (kumuliert) [%]
	2,0 µm	0	0	0	0
2,0 µm	3,0 µm	0	0	0	0
3,0 µm	4,0 µm	0	0	0	0
4,0 µm	5,0 µm	4	4	1	1
5,0 µm	7,0 µm	5	9	2	3
7,0 µm	10,0 µm	11	20	4	6
10,0 µm	13,0 µm	18	38	6	12
13,0 µm	19,0 µm	52	90	17	29
19,0 µm	27,0 µm	52	142	17	45
27,0 µm	38,0 µm	83	225	26	72
38,0 µm	75,0 µm	87	312	28	99
75,0 µm	107,0 µm	2	314	1	100
107,0 µm	151,0 µm	0	314	0	100
151,0 µm	214,0 µm	0	314	0	100
214,0 µm	302,0 µm	0	314	0	100
302,0 µm	427,0 µm	0	314	0	100
427,0 µm	600,0 µm	0	314	0	100
600,0 µm		0	314	0	100
