# Supplementary material for: Data compilation on the effect of grain size, temperature, and texture on the strength of a single-phase FCC MnFeNi medium-entropy alloy
Source: Data Brief. 2019 Nov 15;28:104807. doi: 10.1016/j.dib.2019.104807 (PMC6909151; doi:10.1016/j.dib.2019.104807)
Supplement: Multimedia component 1 [file mmc1.zip › MnFeNi_1173K_60min/MnFeNi_1173K_60min_c=25μm.pdf]

Analysebericht

Mar 21, 2018 12:16:19 PM

powered by imagic.ch

1. cumulative Result 1

Number of images	4
Grain size (ASTM)	7.3
Grain size (G643)	7.3
Grain stretching	95 %
Mean chord length	25.3 µm

2. Single Result 1 (MnFeNi Semesterprojekt_MnFeNi_homogenized_8.1mmSW_900°_60min_00064)

Mean chord length	24.2 µm
Grain size (ASTM)	7.5
Grain size (G643)	7.4
Grain stretching	99.7 %

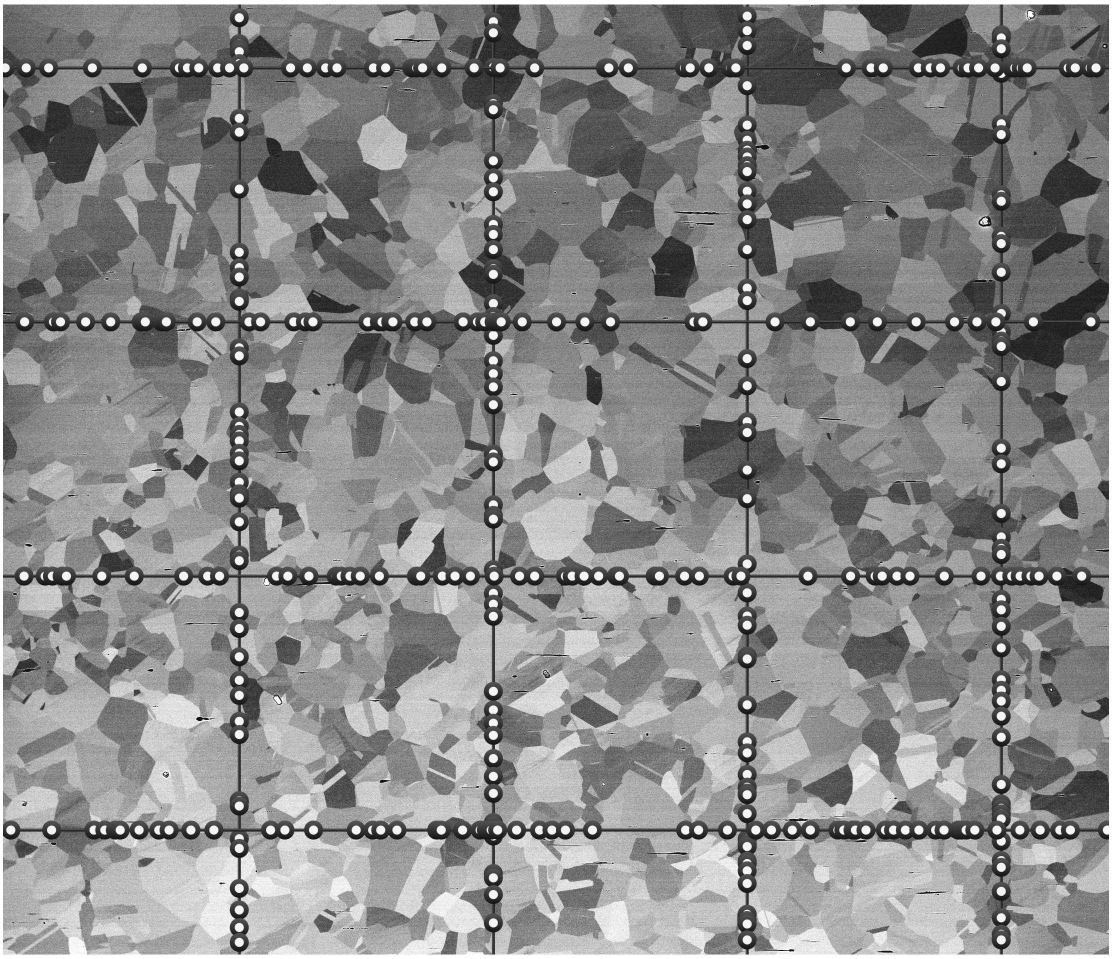

2.1. Statistical Analysis

Statistical Data	Length
Object Count	390
Minimum	1.5 µm
Maximum	126.7 µm
Average	24.2 µm
Standard deviation	18.6 µm
Skewness	0.0
Standard deviation (n-1)	18.6 µm
Variance	345.6 µm²
Variance (n-1)	346.5 µm²
Sum	9'434.9 µm
Sum of squares	363'051.4 µm²

Statistical Data	Length
Sum of cubes	19'423'517.9 µm³

2.1.1. Chord Lenght Distribution

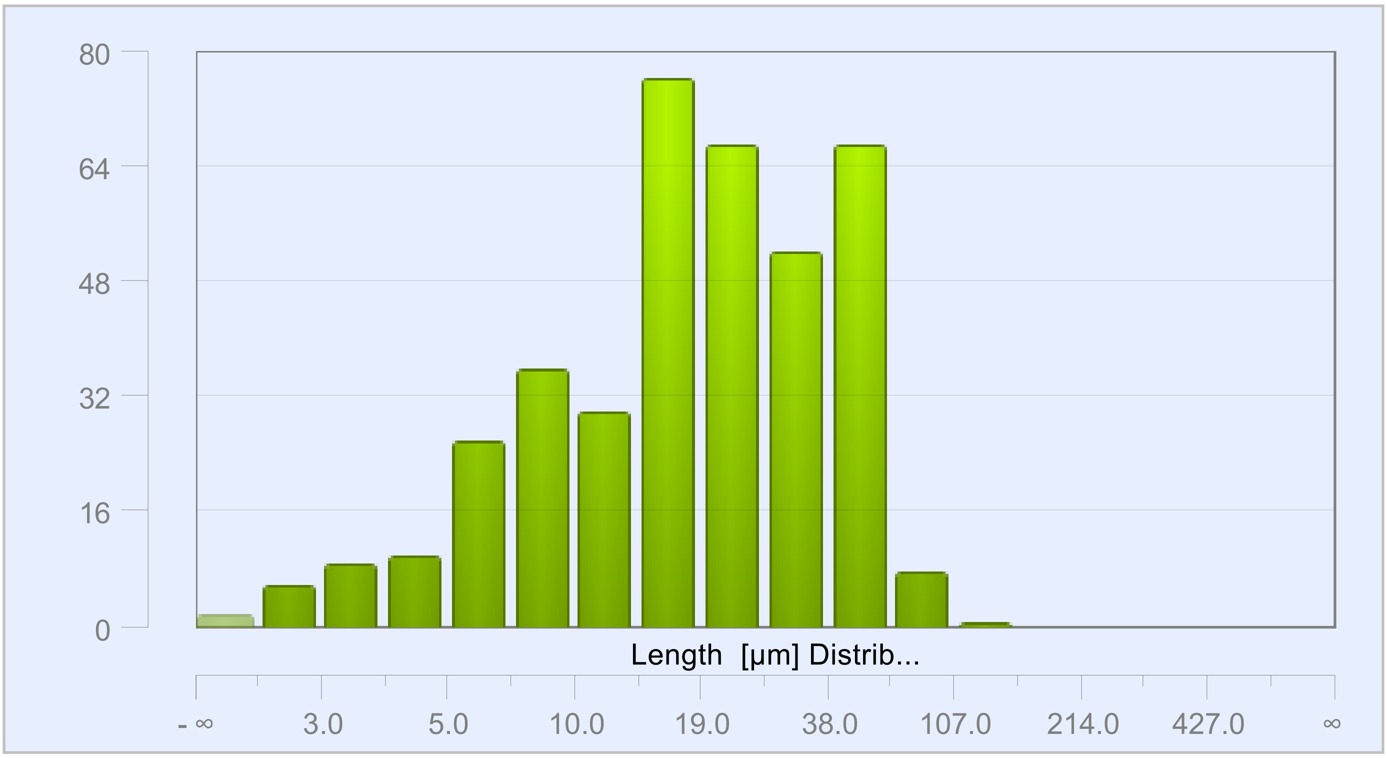

Start	End	Absolute Frequency	Absolute Frequency (accumulated)	Relative Frequency [%]	Relative Frequency (accumulated) [%]
	2.0 µm	2	2	1	1
2.0 µm	3.0 µm	6	8	2	2
3.0 µm	4.0 µm	9	17	2	4
4.0 µm	5.0 µm	10	27	3	7
5.0 µm	7.0 µm	26	53	7	14
7.0 µm	10.0 µm	36	89	9	23
10.0 µm	13.0 µm	30	119	8	31
13.0 µm	19.0 µm	76	195	19	50
19.0 µm	27.0 µm	67	262	17	67
27.0 µm	38.0 µm	52	314	13	81
38.0 µm	75.0 µm	67	381	17	98
75.0 µm	107.0 µm	8	389	2	100
107.0 µm	151.0 µm	1	390	0	100
151.0 µm	214.0 µm	0	390	0	100
214.0 µm	302.0 µm	0	390	0	100
302.0 µm	427.0 µm	0	390	0	100
427.0 µm	600.0 µm	0	390	0	100
600.0 µm		0	390	0	100

3. Single Result 2 (MnFeNi Semesterprojekt_MnFeNi_homogenized_8.1mmSW_900°_60min_00065)

Mean chord length	24.3 µm
Grain size (ASTM)	7.4
Grain size (G643)	7.4
Grain stretching	80.6 %

3.1. Statistical Analysis

Statistical Data	Length
Object Count	388
Minimum	1.5 µm
Maximum	101.0 µm
Average	24.3 µm
Standard deviation	18.6 µm
Skewness	0.0
Standard deviation (n-1)	18.6 µm
Variance	345.5 µm²
Variance (n-1)	346.4 µm²
Sum	9'423.8 µm
Sum of squares	362'956.2 µm²
Sum of cubes	18'804'571.2 µm³

3.1.1. Chord Lenght Distribution

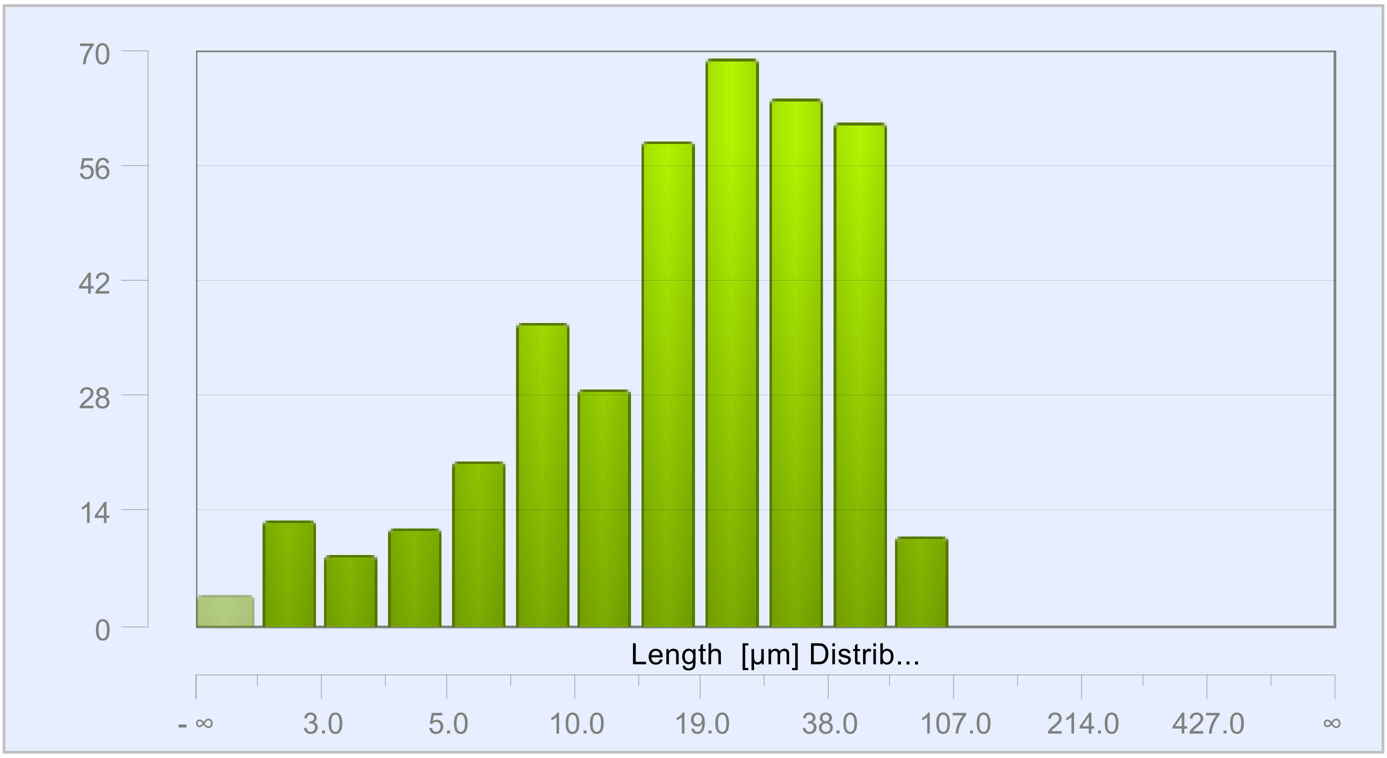

Start	End	Absolute Frequency	Absolute Frequency (accumulated)	Relative Frequency [%]	Relative Frequency (accumulated) [%]
	2.0 µm	4	4	1	1
2.0 µm	3.0 µm	13	17	3	4
3.0 µm	4.0 µm	9	26	2	7
4.0 µm	5.0 µm	12	38	3	10
5.0 µm	7.0 µm	20	58	5	15
7.0 µm	10.0 µm	37	95	10	24
10.0 µm	13.0 µm	29	124	7	32
13.0 µm	19.0 µm	59	183	15	47
19.0 µm	27.0 µm	69	252	18	65
27.0 µm	38.0 µm	64	316	16	81
38.0 µm	75.0 µm	61	377	16	97
75.0 µm	107.0 µm	11	388	3	100

Start	End	Absolute Frequency	Absolute Frequency (accumulated)	Relative Frequency [%]	Relative Frequency (accumulated) [%]
107.0 µm	151.0 µm	0	388	0	100
151.0 µm	214.0 µm	0	388	0	100
214.0 µm	302.0 µm	0	388	0	100
302.0 µm	427.0 µm	0	388	0	100
427.0 µm	600.0 µm	0	388	0	100
600.0 µm		0	388	0	100

4. Single Result 3 (MnFeNi Semesterprojekt_MnFeNi_homogenized_8.1mmSW_900°_60min_00066)

Mean chord length	25.5 µm
Grain size (ASTM)	7.3
Grain size (G643)	7.2
Grain stretching	94.4 %

4.1. Statistical Analysis

Statistical Data	Length
Object Count	369
Minimum	2.2 µm
Maximum	139.7 µm
Average	25.5 µm
Standard deviation	19.2 µm
Skewness	0.0
Standard deviation (n-1)	19.2 µm
Variance	368.6 µm²
Variance (n-1)	369.6 µm²
Sum	9'423.8 µm
Sum of squares	376'694.1 µm²
Sum of cubes	21'770'395.3 µm³

4.1.1. Chord Lenght Distribution

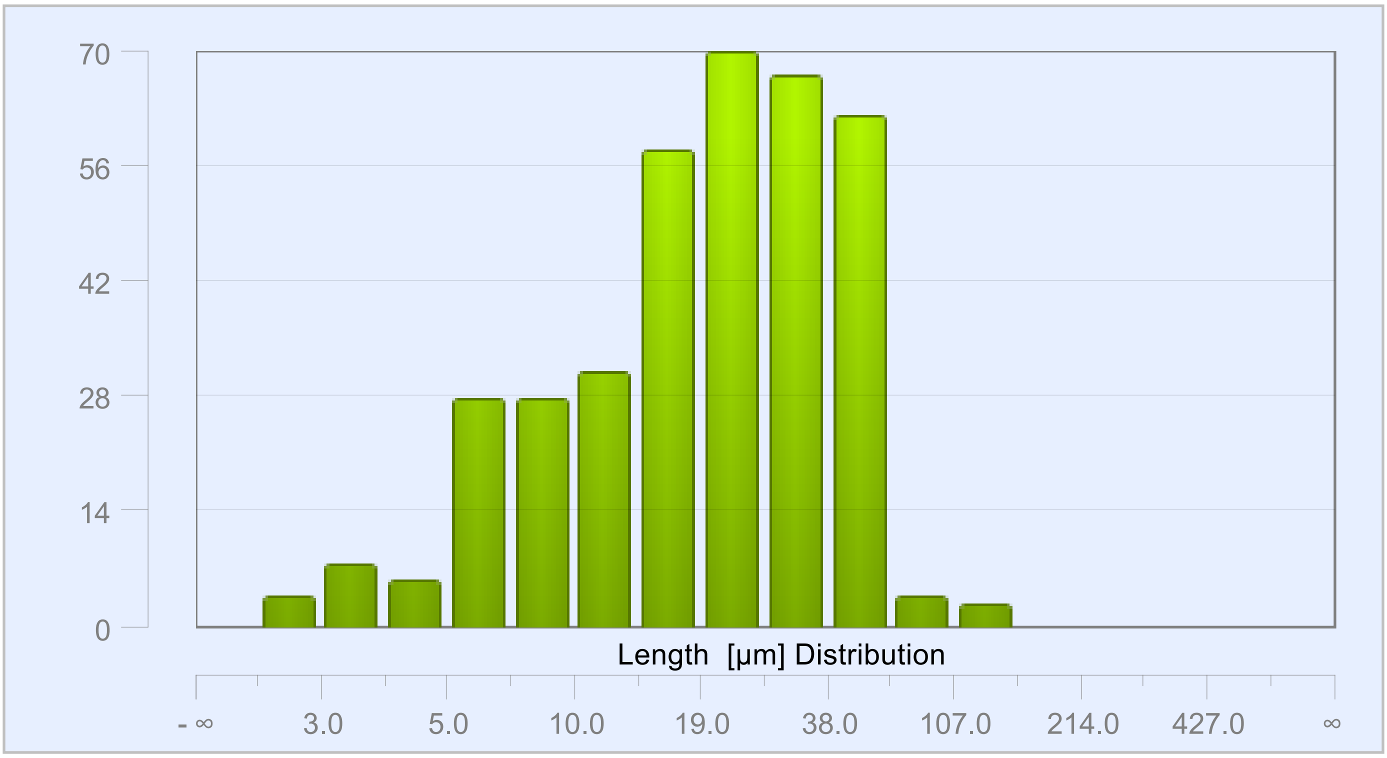

Start	End	Absolute Frequency	Absolute Frequency (accumulated)	Relative Frequency [%]	Relative Frequency (accumulated) [%]
	2.0 µm	0	0	0	0
2.0 µm	3.0 µm	4	4	1	1
3.0 µm	4.0 µm	8	12	2	3
4.0 µm	5.0 µm	6	18	2	5
5.0 µm	7.0 µm	28	46	8	12
7.0 µm	10.0 µm	28	74	8	20
10.0 µm	13.0 µm	31	105	8	28
13.0 µm	19.0 µm	58	163	16	44
19.0 µm	27.0 µm	70	233	19	63
27.0 µm	38.0 µm	67	300	18	81
38.0 µm	75.0 µm	62	362	17	98
75.0 µm	107.0 µm	4	366	1	99
107.0 µm	151.0 µm	3	369	1	100
151.0 µm	214.0 µm	0	369	0	100
214.0 µm	302.0 µm	0	369	0	100
302.0 µm	427.0 µm	0	369	0	100
427.0 µm	600.0 µm	0	369	0	100
600.0 µm		0	369	0	100

5. Single Result 4 (MnFeNi Semesterprojekt_MnFeNi_homogenized_8.1mmSW_900°_60min_00067)

Mean chord length	27.2 µm
Grain size (ASTM)	7.1
Grain size (G643)	7.1
Grain stretching	95.7 %

5.1. Statistical Analysis

Statistical Data	Length
Object Count	348
Minimum	1.6 µm
Maximum	135.4 µm
Average	27.2 µm
Standard deviation	21.0 µm
Skewness	0.0
Standard deviation (n-1)	21.0 µm
Variance	441.7 µm²
Variance (n-1)	442.9 µm²
Sum	9'474.5 µm
Sum of squares	411'647.7 µm²
Sum of cubes	24'569'930.1 µm³

5.1.1. Chord Lenght Distribution

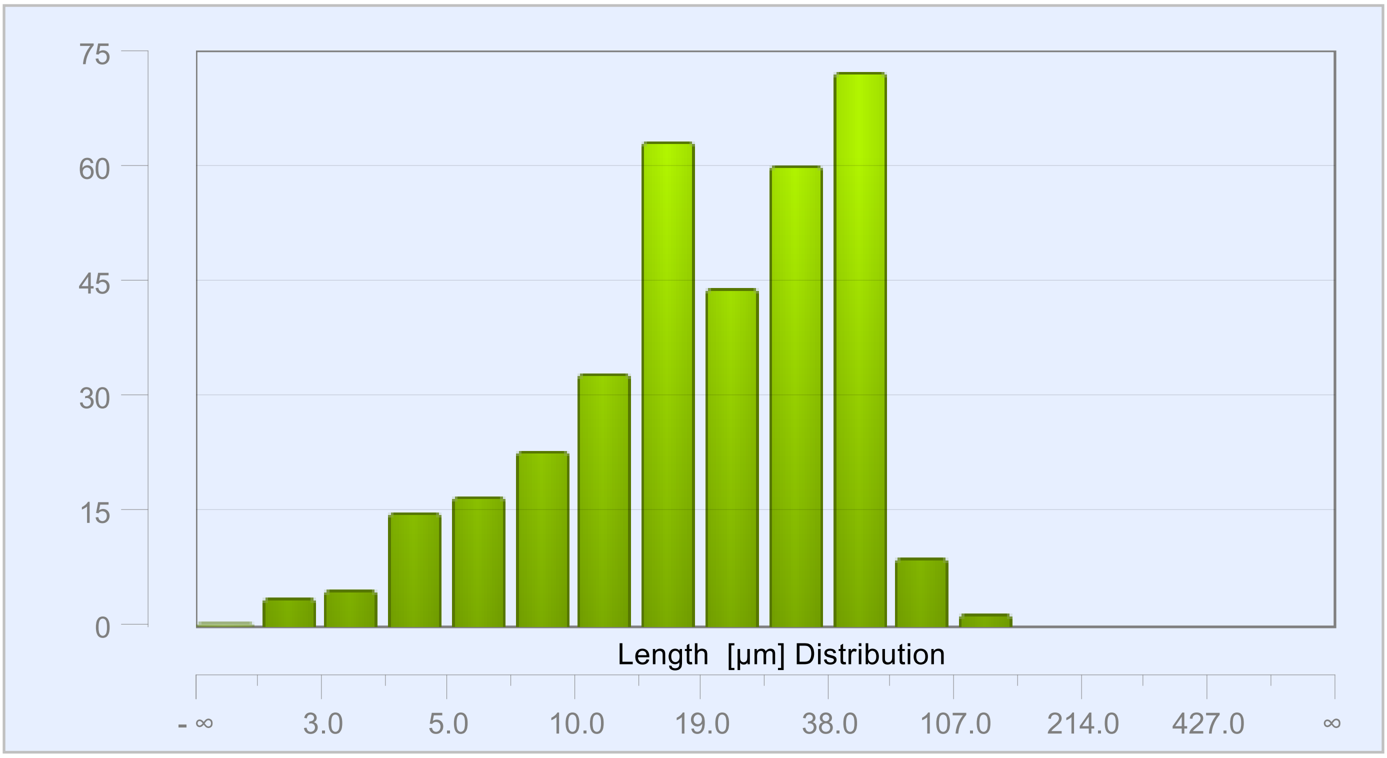

Start	End	Absolute Frequency	Absolute Frequency (accumulated)	Relative Frequency [%]	Relative Frequency (accumulated) [%]
	2.0 µm	1	1	0	0
2.0 µm	3.0 µm	4	5	1	1
3.0 µm	4.0 µm	5	10	1	3
4.0 µm	5.0 µm	15	25	4	7
5.0 µm	7.0 µm	17	42	5	12
7.0 µm	10.0 µm	23	65	7	19
10.0 µm	13.0 µm	33	98	9	28
13.0 µm	19.0 µm	63	161	18	46
19.0 µm	27.0 µm	44	205	13	59
27.0 µm	38.0 µm	60	265	17	76
38.0 µm	75.0 µm	72	337	21	97
75.0 µm	107.0 µm	9	346	3	99
107.0 µm	151.0 µm	2	348	1	100
151.0 µm	214.0 µm	0	348	0	100
214.0 µm	302.0 µm	0	348	0	100
302.0 µm	427.0 µm	0	348	0	100
427.0 µm	600.0 µm	0	348	0	100
600.0 µm		0	348	0	100
