# Supplementary material for: Data compilation on the effect of grain size, temperature, and texture on the strength of a single-phase FCC MnFeNi medium-entropy alloy
Source: Data Brief. 2019 Nov 15;28:104807. doi: 10.1016/j.dib.2019.104807 (PMC6909151; doi:10.1016/j.dib.2019.104807)
Supplement: Multimedia component 1 [file mmc1.zip › MnFeNi_1173K_60min/MnFeNi_1173K_60min_d=33μm.pdf]

Analysebericht

Mar 21, 2018 11:33:17 AM

powered by imagic.ch

1. cumulative Result 1

Number of images	4
Grain size (ASTM)	6.5
Grain size (G643)	6.5
Grain stretching	96.7 %
Mean chord length	33.3 µm

2. Single Result 1 (MnFeNi Semesterprojekt_MnFeNi_homogenized_8.1mmSW_900°_60min_00064)

Mean chord length	31.8 µm
Grain size (ASTM)	6.7
Grain size (G643)	6.6
Grain stretching	92.5 %

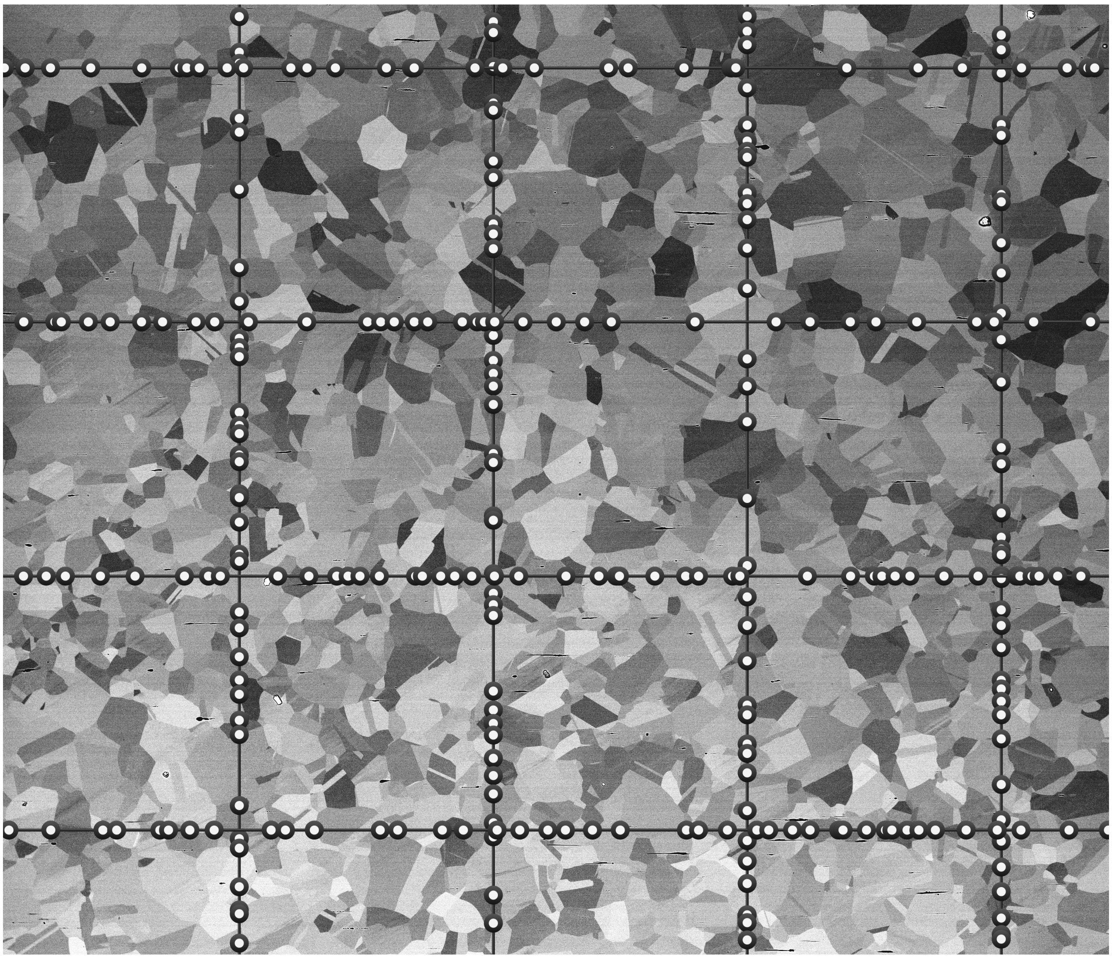

2.1. Statistical Analysis

Statistical Data	Length
Object Count	297
Minimum	0.3 µm
Maximum	127.3 µm
Average	31.8 µm
Standard deviation	20.8 µm
Skewness	0.0
Standard deviation (n-1)	20.9 µm
Variance	433.3 µm²
Variance (n-1)	434.8 µm²
Sum	9'434.9 µm
Sum of squares	428'418.7 µm²

Statistical Data	Length
Sum of cubes	24'877'098.5 µm³

2.1.1. Chord Lenght Distribution

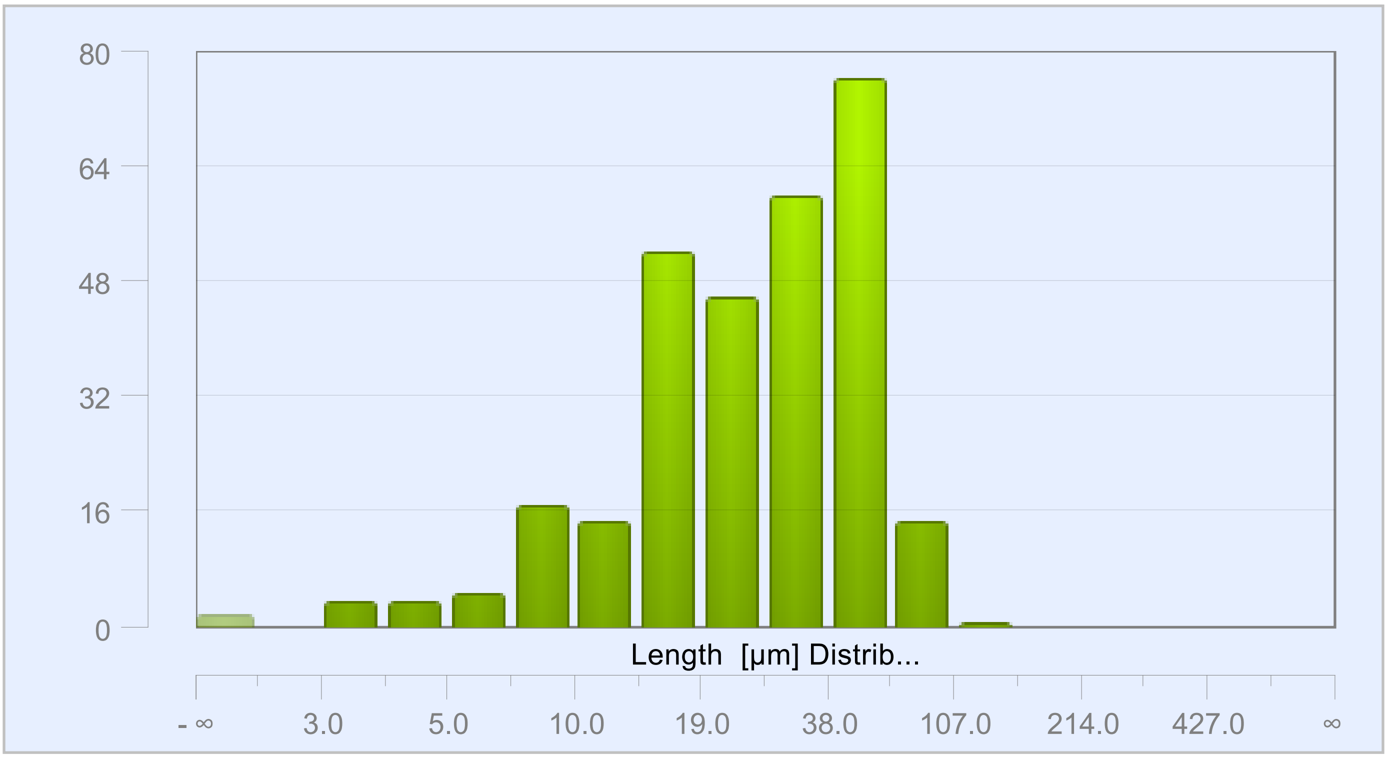

Start	End	Absolute Frequency	Absolute Frequency (accumulated)	Relative Frequency [%]	Relative Frequency (accumulated) [%]
	2.0 µm	2	2	1	1
2.0 µm	3.0 µm	0	2	0	1
3.0 µm	4.0 µm	4	6	1	2
4.0 µm	5.0 µm	4	10	1	3
5.0 µm	7.0 µm	5	15	2	5
7.0 µm	10.0 µm	17	32	6	11
10.0 µm	13.0 µm	15	47	5	16
13.0 µm	19.0 µm	52	99	18	33
19.0 µm	27.0 µm	46	145	15	49
27.0 µm	38.0 µm	60	205	20	69
38.0 µm	75.0 µm	76	281	26	95
75.0 µm	107.0 µm	15	296	5	100
107.0 µm	151.0 µm	1	297	0	100
151.0 µm	214.0 µm	0	297	0	100
214.0 µm	302.0 µm	0	297	0	100
302.0 µm	427.0 µm	0	297	0	100
427.0 µm	600.0 µm	0	297	0	100
600.0 µm		0	297	0	100

3. Single Result 2 (MnFeNi Semesterprojekt_MnFeNi_homogenized_8.1mmSW_900°_60min_00065)

Mean chord length	33 µm
Grain size (ASTM)	6.6
Grain size (G643)	6.5
Grain stretching	86.9 %

3.1. Statistical Analysis

Statistical Data	Length
Object Count	286
Minimum	2.8 µm
Maximum	106.9 µm
Average	33.0 µm
Standard deviation	21.5 µm
Skewness	0.0
Standard deviation (n-1)	21.6 µm
Variance	464.0 µm²
Variance (n-1)	465.6 µm²
Sum	9'423.8 µm
Sum of squares	443'214.3 µm²
Sum of cubes	26'651'435.3 µm³

3.1.1. Chord Lenght Distribution

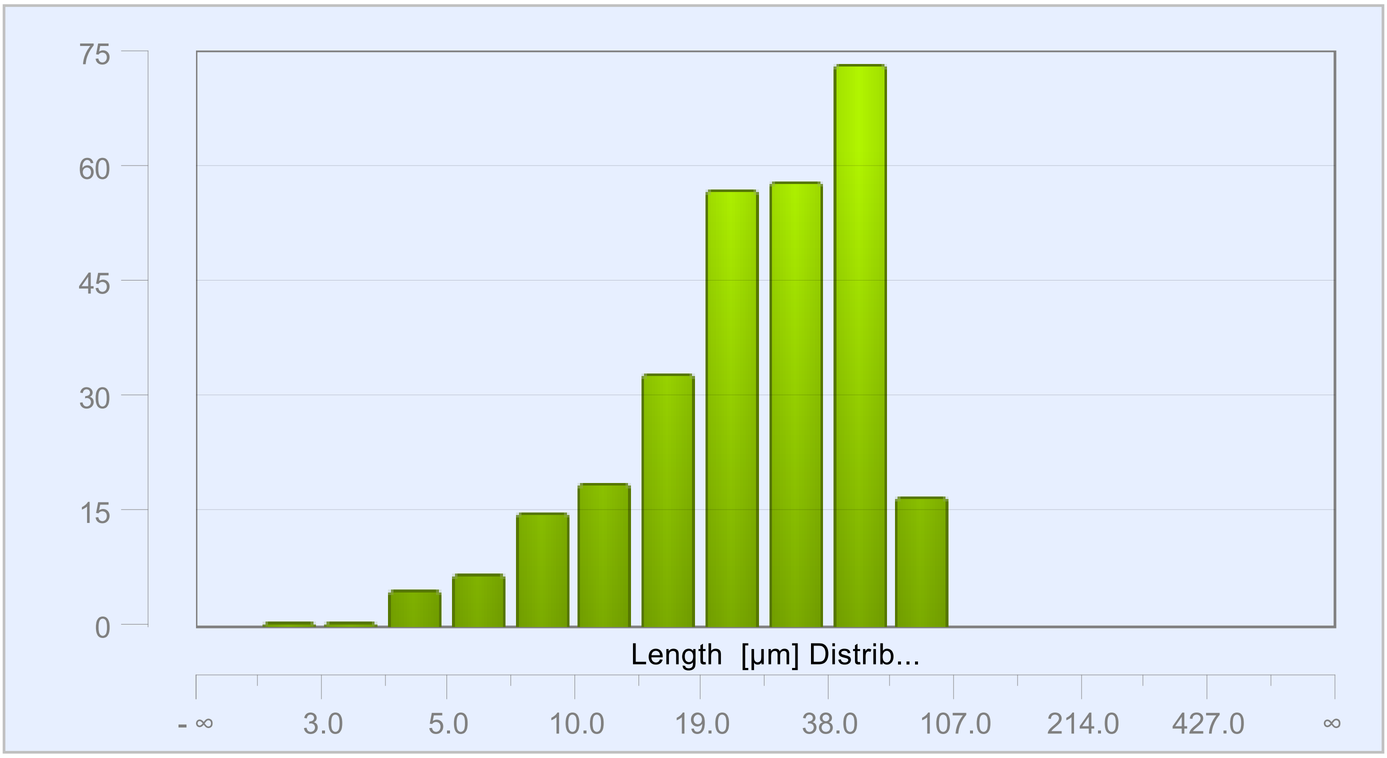

Start	End	Absolute Frequency	Absolute Frequency (accumulated)	Relative Frequency [%]	Relative Frequency (accumulated) [%]
	2.0 µm	0	0	0	0
2.0 µm	3.0 µm	1	1	0	0
3.0 µm	4.0 µm	1	2	0	1
4.0 µm	5.0 µm	5	7	2	2
5.0 µm	7.0 µm	7	14	2	5
7.0 µm	10.0 µm	15	29	5	10
10.0 µm	13.0 µm	19	48	7	17
13.0 µm	19.0 µm	33	81	12	28
19.0 µm	27.0 µm	57	138	20	48
27.0 µm	38.0 µm	58	196	20	69
38.0 µm	75.0 µm	73	269	26	94
75.0 µm	107.0 µm	17	286	6	100

Start	End	Absolute Frequency	Absolute Frequency (accumulated)	Relative Frequency [%]	Relative Frequency (accumulated) [%]
107.0 µm	151.0 µm	0	286	0	100
151.0 µm	214.0 µm	0	286	0	100
214.0 µm	302.0 µm	0	286	0	100
302.0 µm	427.0 µm	0	286	0	100
427.0 µm	600.0 µm	0	286	0	100
600.0 µm		0	286	0	100

4. Single Result 3 (MnFeNi Semesterprojekt_MnFeNi_homogenized_8.1mmSW_900°_60min_00066)

Mean chord length	33.1 µm
Grain size (ASTM)	6.5
Grain size (G643)	6.5
Grain stretching	93.9 %

4.1. Statistical Analysis

Statistical Data	Length
Object Count	285
Minimum	1.5 µm
Maximum	114.9 µm
Average	33.1 µm
Standard deviation	19.8 µm
Skewness	0.0
Standard deviation (n-1)	19.8 µm
Variance	390.9 µm²
Variance (n-1)	392.3 µm²
Sum	9'423.8 µm
Sum of squares	423'022.3 µm²
Sum of cubes	23'312'220.5 µm³

4.1.1. Chord Lenght Distribution

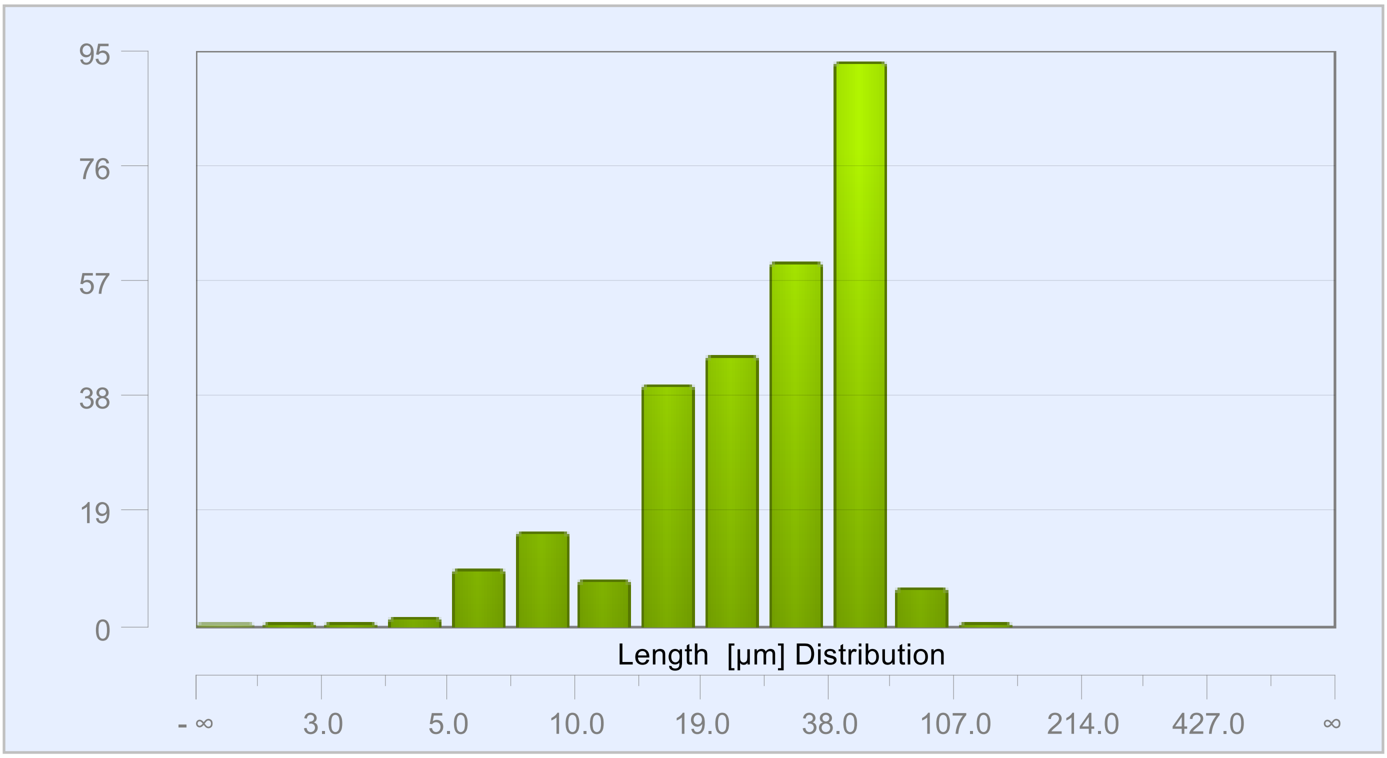

Start	End	Absolute Frequency	Absolute Frequency (accumulated)	Relative Frequency [%]	Relative Frequency (accumulated) [%]
	2.0 µm	1	1	0	0
2.0 µm	3.0 µm	1	2	0	1
3.0 µm	4.0 µm	1	3	0	1
4.0 µm	5.0 µm	2	5	1	2
5.0 µm	7.0 µm	10	15	4	5
7.0 µm	10.0 µm	16	31	6	11
10.0 µm	13.0 µm	8	39	3	14
13.0 µm	19.0 µm	40	79	14	28
19.0 µm	27.0 µm	45	124	16	44
27.0 µm	38.0 µm	60	184	21	65
38.0 µm	75.0 µm	93	277	33	97
75.0 µm	107.0 µm	7	284	2	100
107.0 µm	151.0 µm	1	285	0	100
151.0 µm	214.0 µm	0	285	0	100
214.0 µm	302.0 µm	0	285	0	100
302.0 µm	427.0 µm	0	285	0	100
427.0 µm	600.0 µm	0	285	0	100
600.0 µm		0	285	0	100

5. Single Result 4 (MnFeNi Semesterprojekt_MnFeNi_homogenized_8.1mmSW_900°_60min_00067)

Mean chord length	35.8 µm
Grain size (ASTM)	6.3
Grain size (G643)	6.3
Grain stretching	84.9 %

5.1. Statistical Analysis

Statistical Data	Length
Object Count	265
Minimum	2.2 µm
Maximum	140.1 µm
Average	35.8 µm
Standard deviation	21.5 µm
Skewness	0.0
Standard deviation (n-1)	21.5 µm
Variance	462.4 µm²
Variance (n-1)	464.1 µm²
Sum	9'474.5 µm
Sum of squares	461'271.9 µm²
Sum of cubes	27'935'921.1 µm³

5.1.1. Chord Lenght Distribution

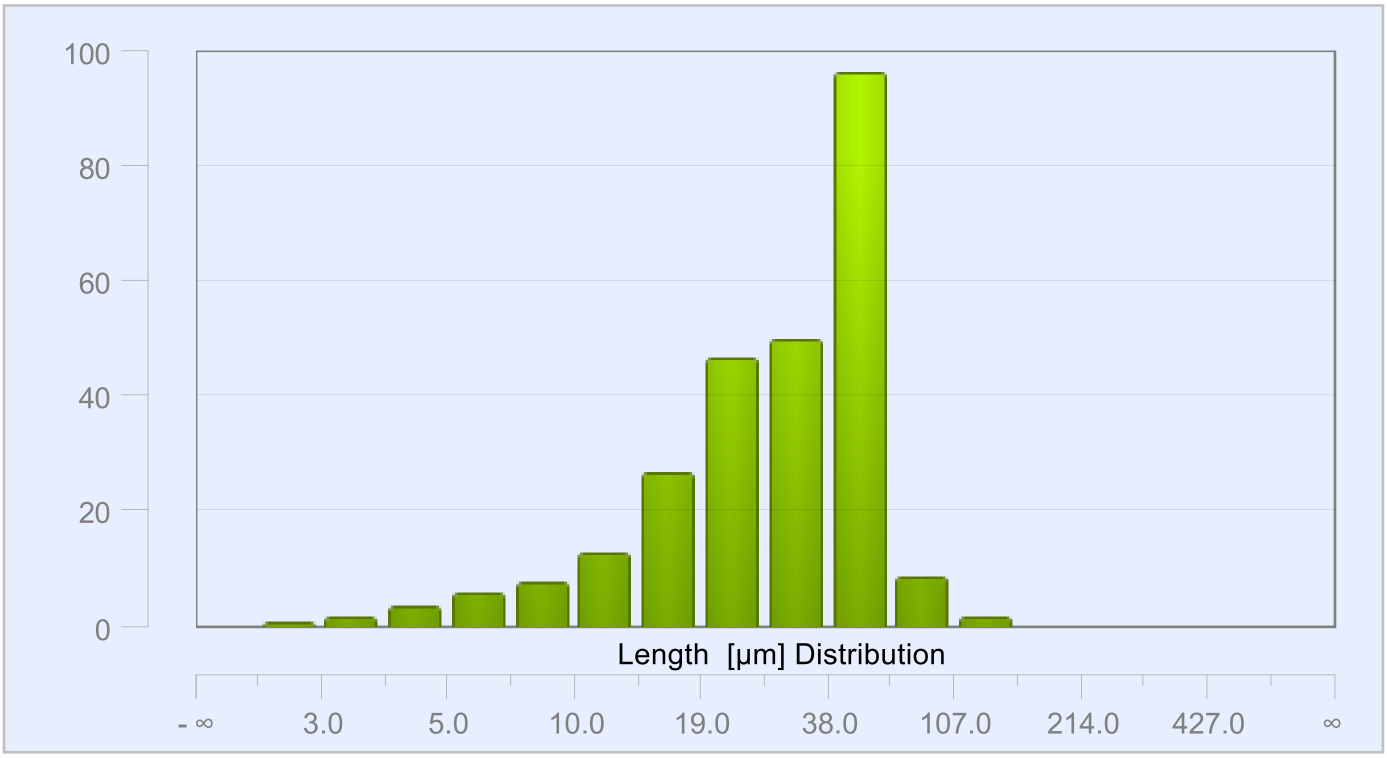

Start	End	Absolute Frequency	Absolute Frequency (accumulated)	Relative Frequency [%]	Relative Frequency (accumulated) [%]
	2.0 µm	0	0	0	0
2.0 µm	3.0 µm	1	1	0	0
3.0 µm	4.0 µm	2	3	1	1
4.0 µm	5.0 µm	4	7	2	3
5.0 µm	7.0 µm	6	13	2	5
7.0 µm	10.0 µm	8	21	3	8
10.0 µm	13.0 µm	13	34	5	13
13.0 µm	19.0 µm	27	61	10	23
19.0 µm	27.0 µm	47	108	18	41
27.0 µm	38.0 µm	50	158	19	60
38.0 µm	75.0 µm	96	254	36	96
75.0 µm	107.0 µm	9	263	3	99
107.0 µm	151.0 µm	2	265	1	100
151.0 µm	214.0 µm	0	265	0	100
214.0 µm	302.0 µm	0	265	0	100
302.0 µm	427.0 µm	0	265	0	100
427.0 µm	600.0 µm	0	265	0	100
600.0 µm		0	265	0	100
