# Supplementary material for: Data compilation on the effect of grain size, temperature, and texture on the strength of a single-phase FCC MnFeNi medium-entropy alloy
Source: Data Brief. 2019 Nov 15;28:104807. doi: 10.1016/j.dib.2019.104807 (PMC6909151; doi:10.1016/j.dib.2019.104807)
Supplement: Multimedia component 1 [file mmc1.zip › MnFeNi_1273K_60min/MnFeNi_1273K_60min_c=49μm.pdf]

Analysebericht

Mar 21, 2018 12:59:25 PM

powered by imagic.ch

1. cumulative Result 1

Number of images	4
Grain size (ASTM)	5.4
Grain size (G643)	5.4
Grain stretching	88.6 %
Mean chord length	48.5 µm

2. Single Result 1 (MnFeNi Semesterprojekt_MnFeNi_homogenized_8.1mmSW_1000°C_60min_00060)

Mean chord length	45.3 µm
Grain size (ASTM)	5.6
Grain size (G643)	5.6
Grain stretching	91.5 %

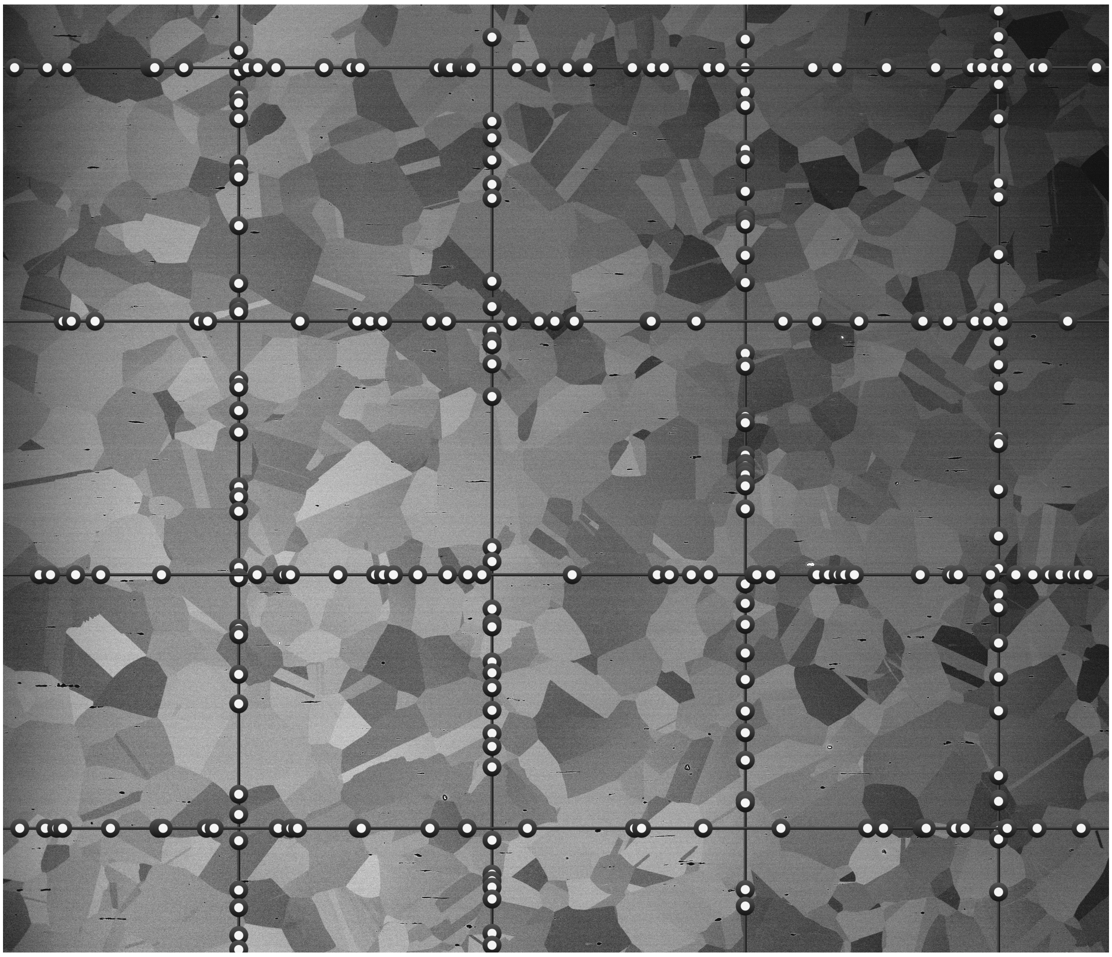

2.1. Statistical Analysis

Statistical Data	Length
Object Count	277
Minimum	2.5 µm
Maximum	230.4 µm
Average	45.3 µm
Standard deviation	37.1 µm
Skewness	0.0
Standard deviation (n-1)	37.2 µm
Variance	1'376.7 µm²
Variance (n-1)	1'381.7 µm²
Sum	12'539.2 µm
Sum of squares	948'974.1 µm²

Statistical Data	Length
Sum of cubes	96'963'501.8 µm³

2.1.1. Chord Lenght Distribution

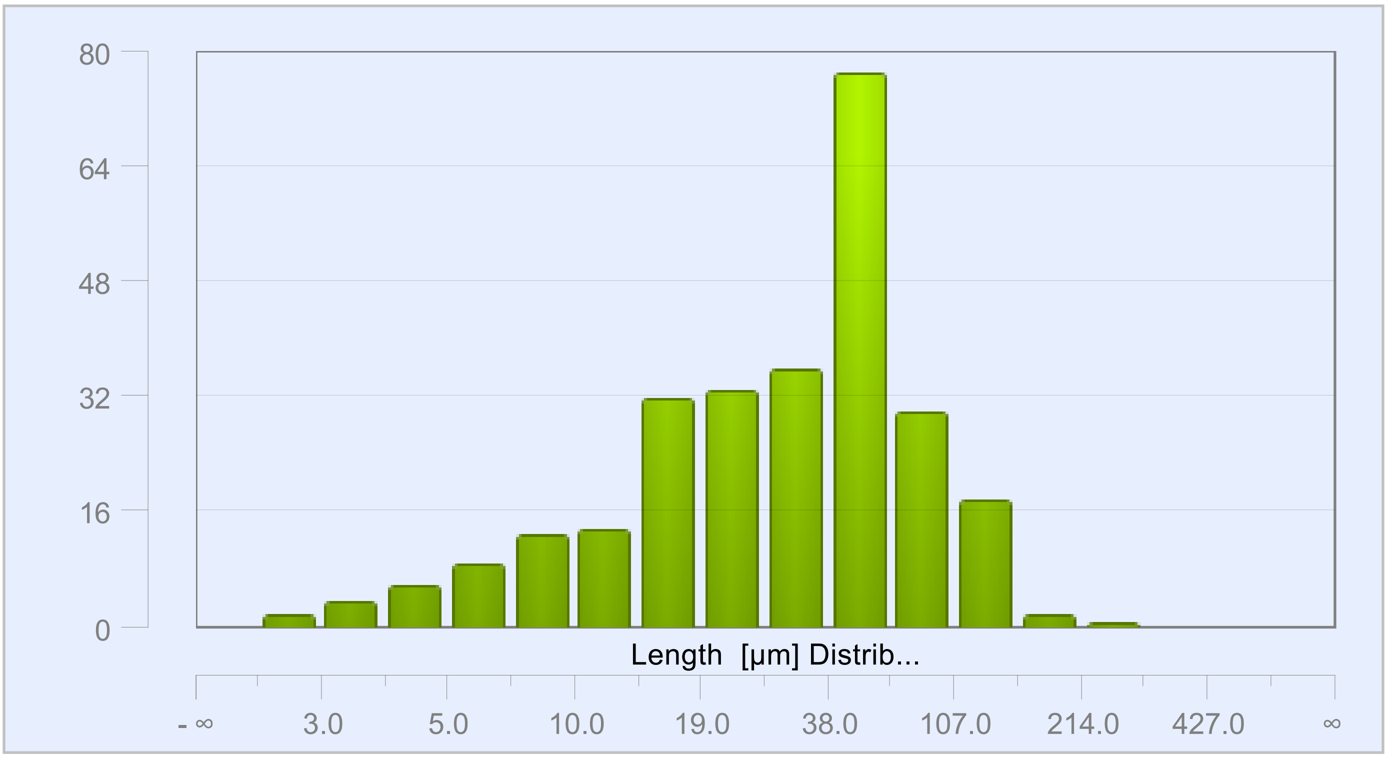

Start	End	Absolute Frequency	Absolute Frequency (accumulated)	Relative Frequency [%]	Relative Frequency (accumulated) [%]
	2.0 µm	0	0	0	0
2.0 µm	3.0 µm	2	2	1	1
3.0 µm	4.0 µm	4	6	1	2
4.0 µm	5.0 µm	6	12	2	4
5.0 µm	7.0 µm	9	21	3	8
7.0 µm	10.0 µm	13	34	5	12
10.0 µm	13.0 µm	14	48	5	17
13.0 µm	19.0 µm	32	80	12	29
19.0 µm	27.0 µm	33	113	12	41
27.0 µm	38.0 µm	36	149	13	54
38.0 µm	75.0 µm	77	226	28	82
75.0 µm	107.0 µm	30	256	11	92
107.0 µm	151.0 µm	18	274	6	99
151.0 µm	214.0 µm	2	276	1	100
214.0 µm	302.0 µm	1	277	0	100
302.0 µm	427.0 µm	0	277	0	100
427.0 µm	600.0 µm	0	277	0	100
600.0 µm		0	277	0	100

3. Single Result 2 (MnFeNi Semesterprojekt_MnFeNi_homogenized_8.1mmSW_1000°C_60min_00061)

Mean chord length	47.6 µm
Grain size (ASTM)	5.5
Grain size (G643)	5.5
Grain stretching	93 %

3.1. Statistical Analysis

Statistical Data	Length
Object Count	264
Minimum	3.3 µm
Maximum	309.2 µm
Average	47.6 µm
Standard deviation	41.1 µm
Skewness	0.0
Standard deviation (n-1)	41.2 µm
Variance	1'687.0 µm²
Variance (n-1)	1'693.5 µm²
Sum	12'575.4 µm
Sum of squares	1'044'398.8 µm²
Sum of cubes	126'702'146.3 µm³

3.1.1. Chord Lenght Distribution

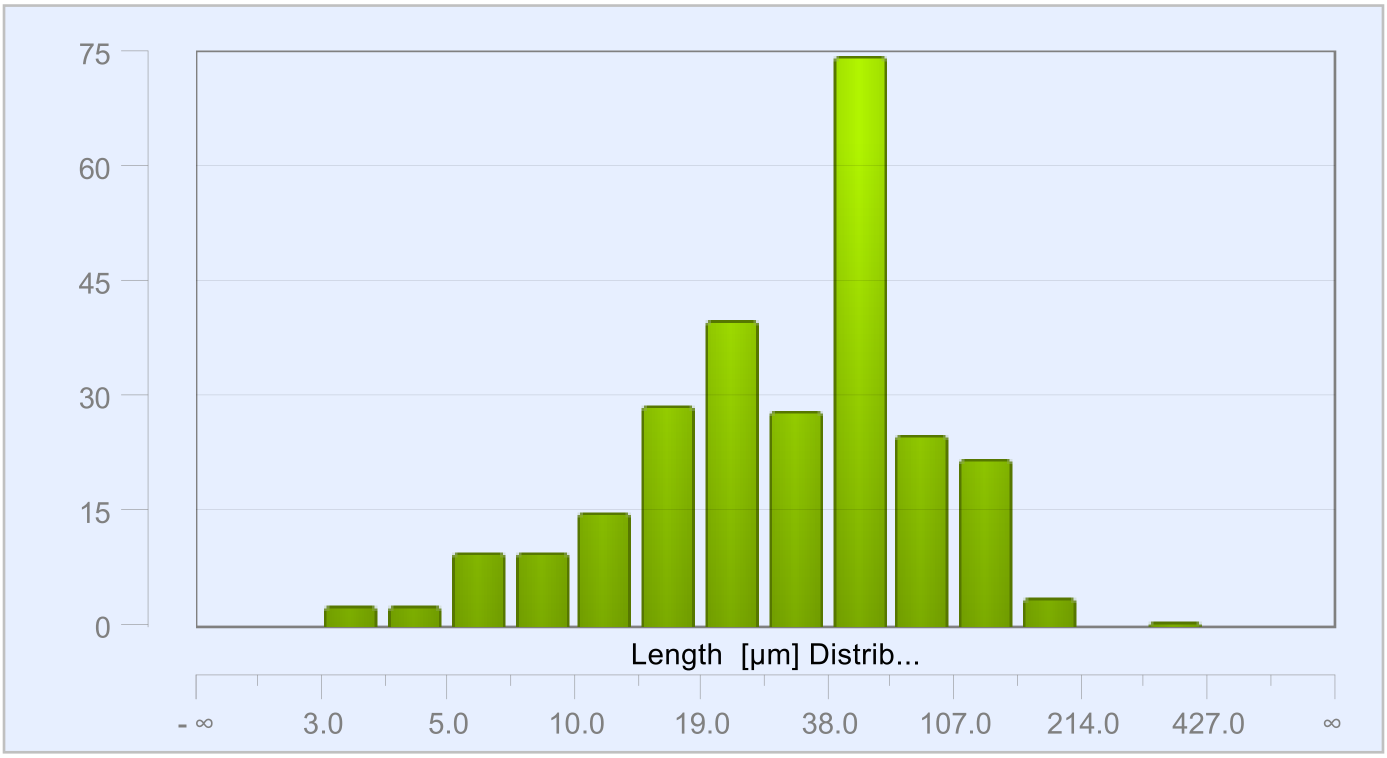

Start	End	Absolute Frequency	Absolute Frequency (accumulated)	Relative Frequency [%]	Relative Frequency (accumulated) [%]
	2.0 µm	0	0	0	0
2.0 µm	3.0 µm	0	0	0	0
3.0 µm	4.0 µm	3	3	1	1
4.0 µm	5.0 µm	3	6	1	2
5.0 µm	7.0 µm	10	16	4	6
7.0 µm	10.0 µm	10	26	4	10
10.0 µm	13.0 µm	15	41	6	16
13.0 µm	19.0 µm	29	70	11	27
19.0 µm	27.0 µm	40	110	15	42
27.0 µm	38.0 µm	28	138	11	52
38.0 µm	75.0 µm	74	212	28	80
75.0 µm	107.0 µm	25	237	9	90

Start	End	Absolute Frequency	Absolute Frequency (accumulated)	Relative Frequency [%]	Relative Frequency (accumulated) [%]
107.0 µm	151.0 µm	22	259	8	98
151.0 µm	214.0 µm	4	263	2	100
214.0 µm	302.0 µm	0	263	0	100
302.0 µm	427.0 µm	1	264	0	100
427.0 µm	600.0 µm	0	264	0	100
600.0 µm		0	264	0	100

4. Single Result 3 (MnFeNi Semesterprojekt_MnFeNi_homogenized_8.1mmSW_1000°C_60min_00062)

Mean chord length	54.6 µm
Grain size (ASTM)	5.1
Grain size (G643)	5.1
Grain stretching	94.8 %

4.1. Statistical Analysis

Statistical Data	Length
Object Count	231
Minimum	3.7 µm
Maximum	233.7 µm
Average	54.6 µm
Standard deviation	41.0 µm
Skewness	0.0
Standard deviation (n-1)	41.1 µm
Variance	1'678.2 µm²
Variance (n-1)	1'685.5 µm²
Sum	12'610.4 µm
Sum of squares	1'076'081.2 µm²
Sum of cubes	123'408'816.7 µm³

4.1.1. Chord Lenght Distribution

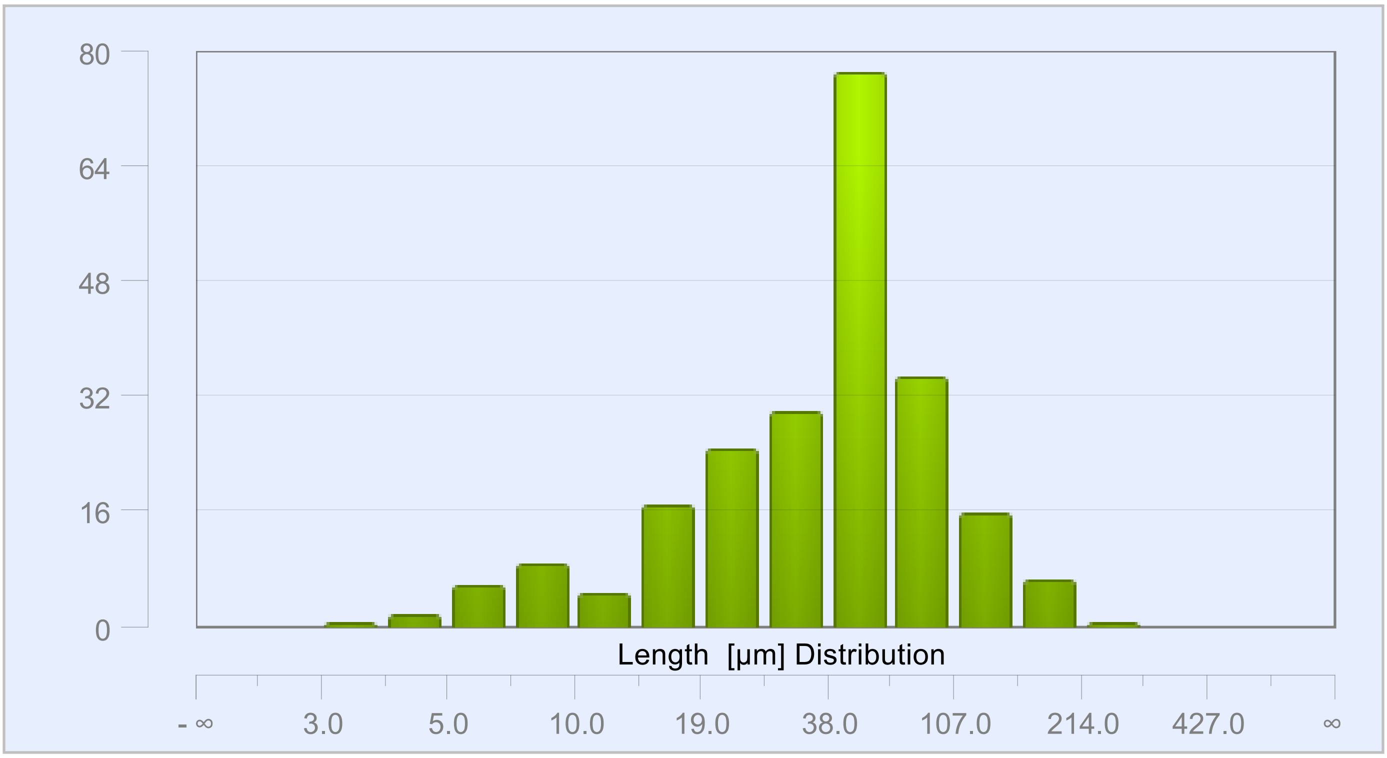

Start	End	Absolute Frequency	Absolute Frequency (accumulated)	Relative Frequency [%]	Relative Frequency (accumulated) [%]
	2.0 µm	0	0	0	0
2.0 µm	3.0 µm	0	0	0	0
3.0 µm	4.0 µm	1	1	0	0
4.0 µm	5.0 µm	2	3	1	1
5.0 µm	7.0 µm	6	9	3	4
7.0 µm	10.0 µm	9	18	4	8
10.0 µm	13.0 µm	5	23	2	10
13.0 µm	19.0 µm	17	40	7	17
19.0 µm	27.0 µm	25	65	11	28
27.0 µm	38.0 µm	30	95	13	41
38.0 µm	75.0 µm	77	172	33	74
75.0 µm	107.0 µm	35	207	15	90
107.0 µm	151.0 µm	16	223	7	97
151.0 µm	214.0 µm	7	230	3	100
214.0 µm	302.0 µm	1	231	0	100
302.0 µm	427.0 µm	0	231	0	100
427.0 µm	600.0 µm	0	231	0	100
600.0 µm		0	231	0	100

5. Single Result 4 (MnFeNi Semesterprojekt_MnFeNi_homogenized_8.1mmSW_1000°C_60min_00063)

Mean chord length	47.6 µm
Grain size (ASTM)	5.5
Grain size (G643)	5.5
Grain stretching	77 %

5.1. Statistical Analysis

Statistical Data	Length
Object Count	265
Minimum	0.8 µm
Maximum	205.5 µm
Average	47.6 µm
Standard deviation	39.2 µm
Skewness	0.0
Standard deviation (n-1)	39.2 µm
Variance	1'533.3 µm²
Variance (n-1)	1'539.1 µm²
Sum	12'618.7 µm
Sum of squares	1'007'199.6 µm²
Sum of cubes	110'822'091.9 µm³

5.1.1. Chord Lenght Distribution

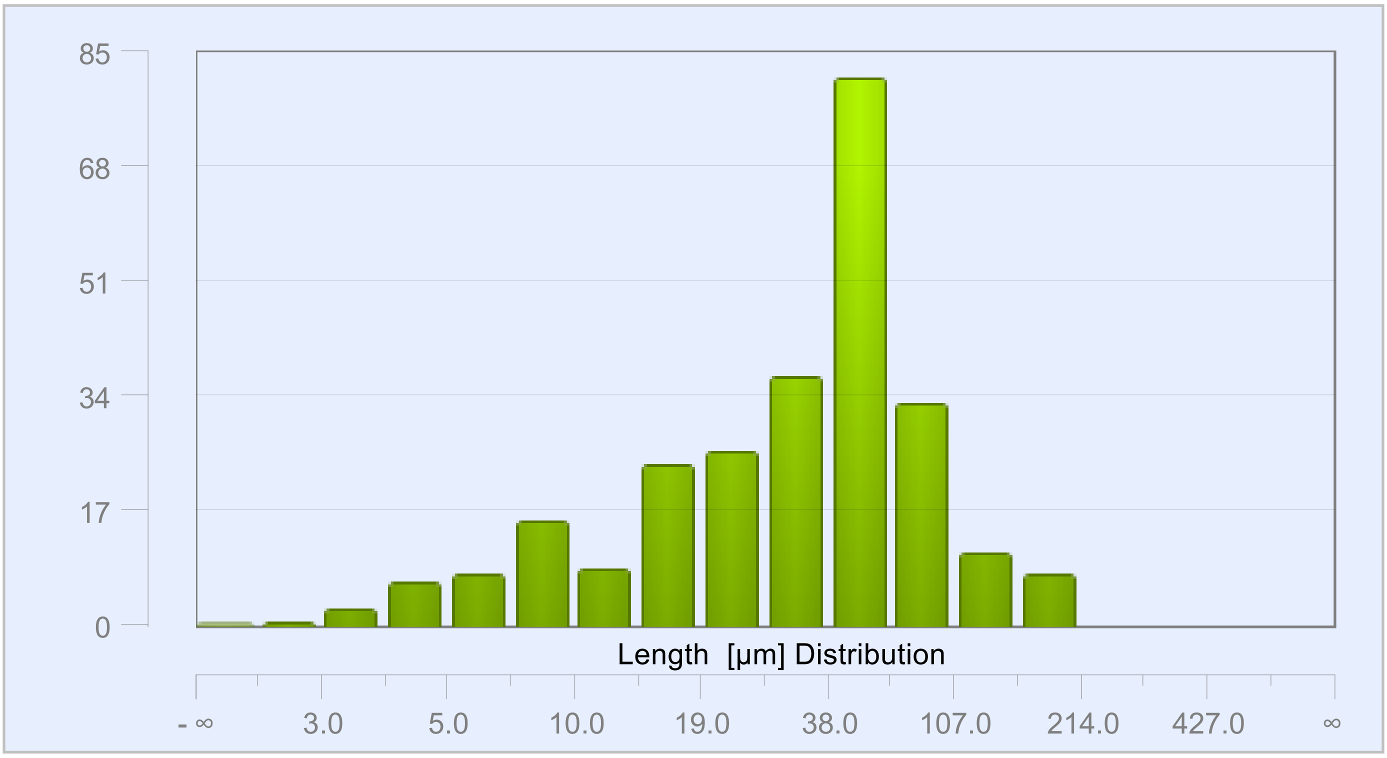

Start	End	Absolute Frequency	Absolute Frequency (accumulated)	Relative Frequency [%]	Relative Frequency (accumulated) [%]
	2.0 µm	1	1	0	0
2.0 µm	3.0 µm	1	2	0	1
3.0 µm	4.0 µm	3	5	1	2
4.0 µm	5.0 µm	7	12	3	5
5.0 µm	7.0 µm	8	20	3	8
7.0 µm	10.0 µm	16	36	6	14
10.0 µm	13.0 µm	9	45	3	17
13.0 µm	19.0 µm	24	69	9	26
19.0 µm	27.0 µm	26	95	10	36
27.0 µm	38.0 µm	37	132	14	50
38.0 µm	75.0 µm	81	213	31	80
75.0 µm	107.0 µm	33	246	12	93
107.0 µm	151.0 µm	11	257	4	97
151.0 µm	214.0 µm	8	265	3	100
214.0 µm	302.0 µm	0	265	0	100
302.0 µm	427.0 µm	0	265	0	100
427.0 µm	600.0 µm	0	265	0	100
600.0 µm		0	265	0	100
