# Supplementary material for: Data compilation on the effect of grain size, temperature, and texture on the strength of a single-phase FCC MnFeNi medium-entropy alloy
Source: Data Brief. 2019 Nov 15;28:104807. doi: 10.1016/j.dib.2019.104807 (PMC6909151; doi:10.1016/j.dib.2019.104807)
Supplement: Multimedia component 1 [file mmc1.zip › MnFeNi_1273K_60min/MnFeNi_1273K_60min_d=66μm.pdf]

Analysebericht

Mar 2, 2018 4:24:11 PM

powered by imagic.ch

1. cumulative Result 1

Number of images	4
Grain size (ASTM)	4.6
Grain size (G643)	4.5
Grain stretching	93.3 %
Mean chord length	65.5 µm

2. Single Result 1 (MnFeNi Semesterprojekt_MnFeNi_homogenized_8.1mmSW_1000°C_60min_00060)

Mean chord length	68.1 µm
Grain size (ASTM)	4.5
Grain size (G643)	4.4
Grain stretching	87.6 %

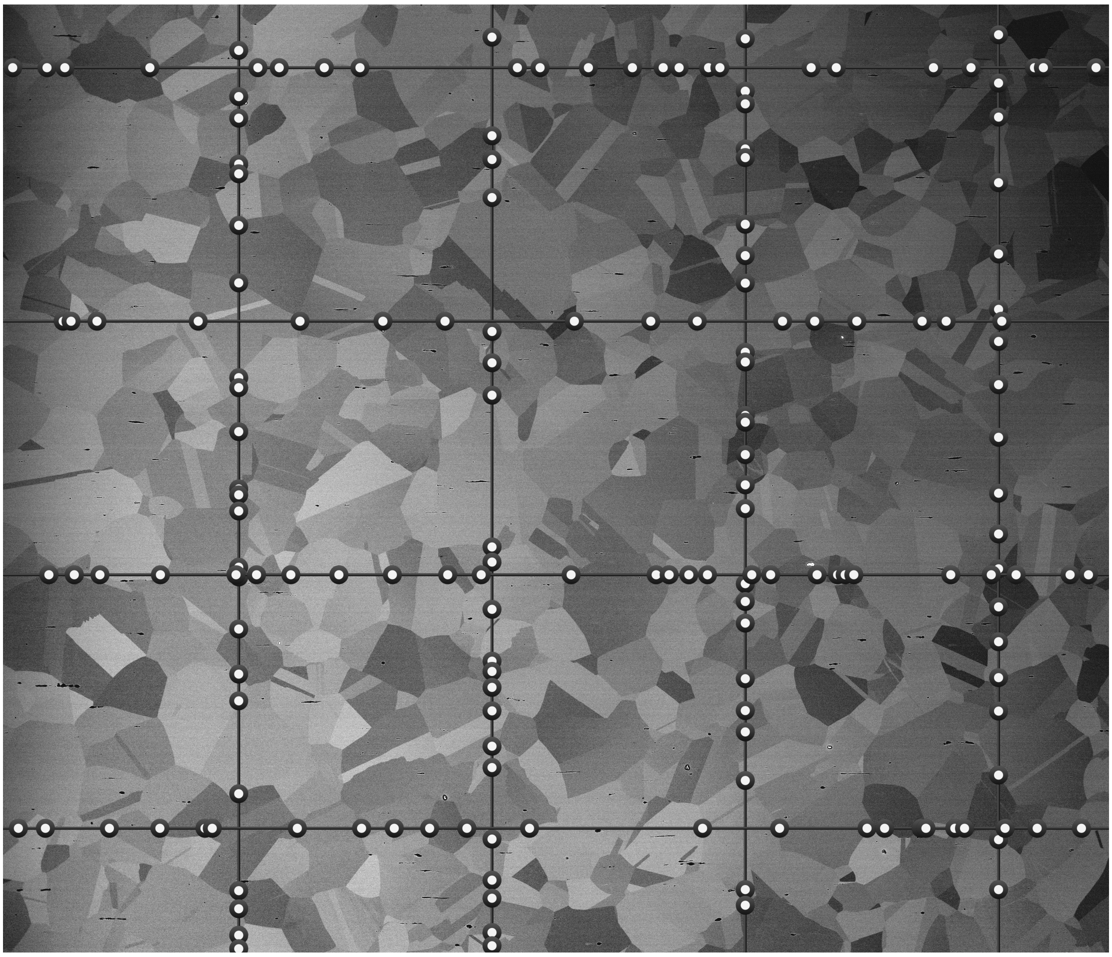

2.1. Statistical Analysis

Statistical Data	Length
Object Count	184
Minimum	5.4 µm
Maximum	264.2 µm
Average	68.1 µm
Standard deviation	46.8 µm
Skewness	0.0
Standard deviation (n-1)	46.9 µm
Variance	2'188.0 µm²
Variance (n-1)	2'199.9 µm²
Sum	12'539.2 µm
Sum of squares	1'257'096.6 µm²

Statistical Data	Length
Sum of cubes	166'436'087.4 µm³

2.1.1. Chord Lenght Distribution

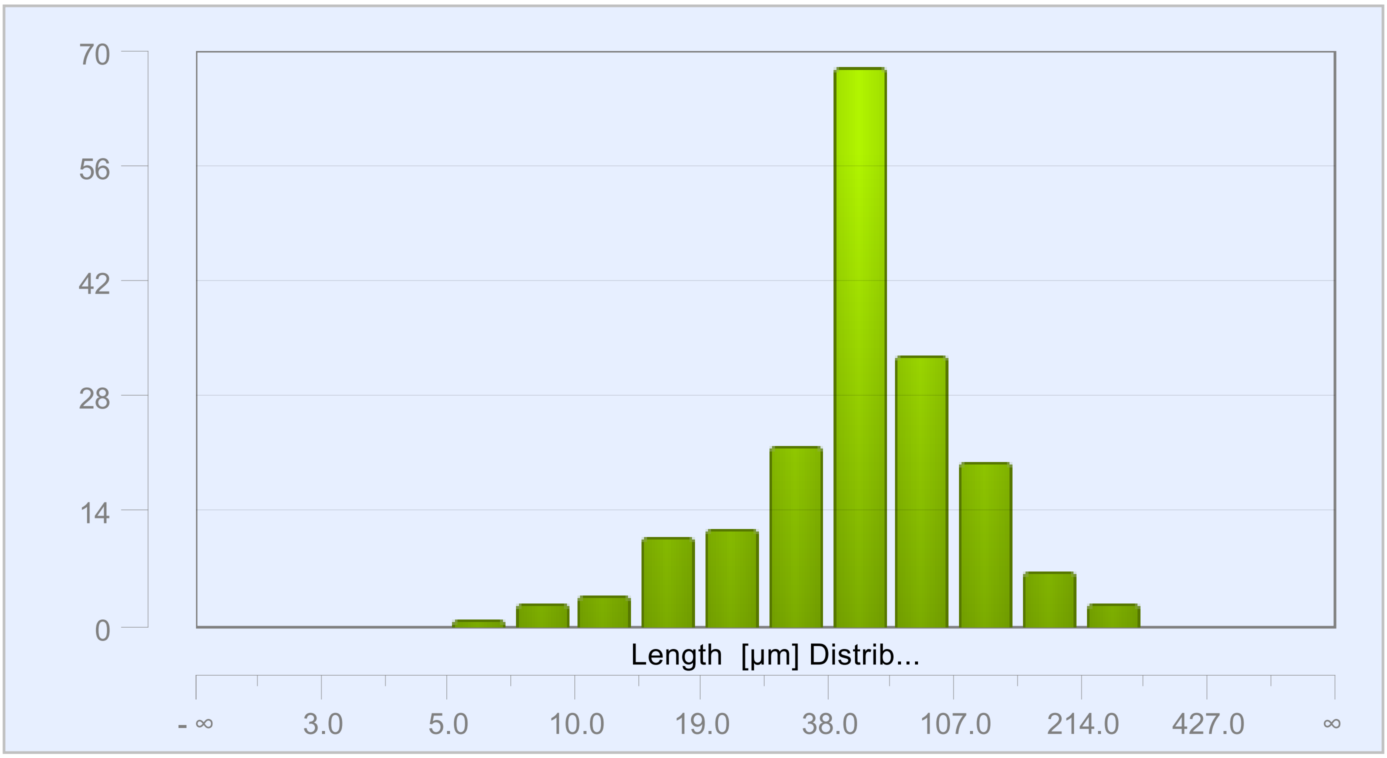

Start	End	Absolute Frequency	Absolute Frequency (accumulated)	Relative Frequency [%]	Relative Frequency (accumulated) [%]
	2.0 µm	0	0	0	0
2.0 µm	3.0 µm	0	0	0	0
3.0 µm	4.0 µm	0	0	0	0
4.0 µm	5.0 µm	0	0	0	0
5.0 µm	7.0 µm	1	1	1	1
7.0 µm	10.0 µm	3	4	2	2
10.0 µm	13.0 µm	4	8	2	4
13.0 µm	19.0 µm	11	19	6	10
19.0 µm	27.0 µm	12	31	7	17
27.0 µm	38.0 µm	22	53	12	29
38.0 µm	75.0 µm	68	121	37	66
75.0 µm	107.0 µm	33	154	18	84
107.0 µm	151.0 µm	20	174	11	95
151.0 µm	214.0 µm	7	181	4	98
214.0 µm	302.0 µm	3	184	2	100
302.0 µm	427.0 µm	0	184	0	100
427.0 µm	600.0 µm	0	184	0	100
600.0 µm		0	184	0	100

3. Single Result 2 (MnFeNi Semesterprojekt_MnFeNi_homogenized_8.1mmSW_1000°C_60min_00061)

Mean chord length	58.8 µm
Grain size (ASTM)	4.9
Grain size (G643)	4.8
Grain stretching	96.5 %

3.1. Statistical Analysis

Statistical Data	Length
Object Count	214
Minimum	2.1 µm
Maximum	181.8 µm
Average	58.8 µm
Standard deviation	41.3 µm
Skewness	0.0
Standard deviation (n-1)	41.4 µm
Variance	1'707.5 µm²
Variance (n-1)	1'715.5 µm²
Sum	12'575.4 µm
Sum of squares	1'104'386.8 µm²
Sum of cubes	121'389'712.3 µm³

3.1.1. Chord Lenght Distribution

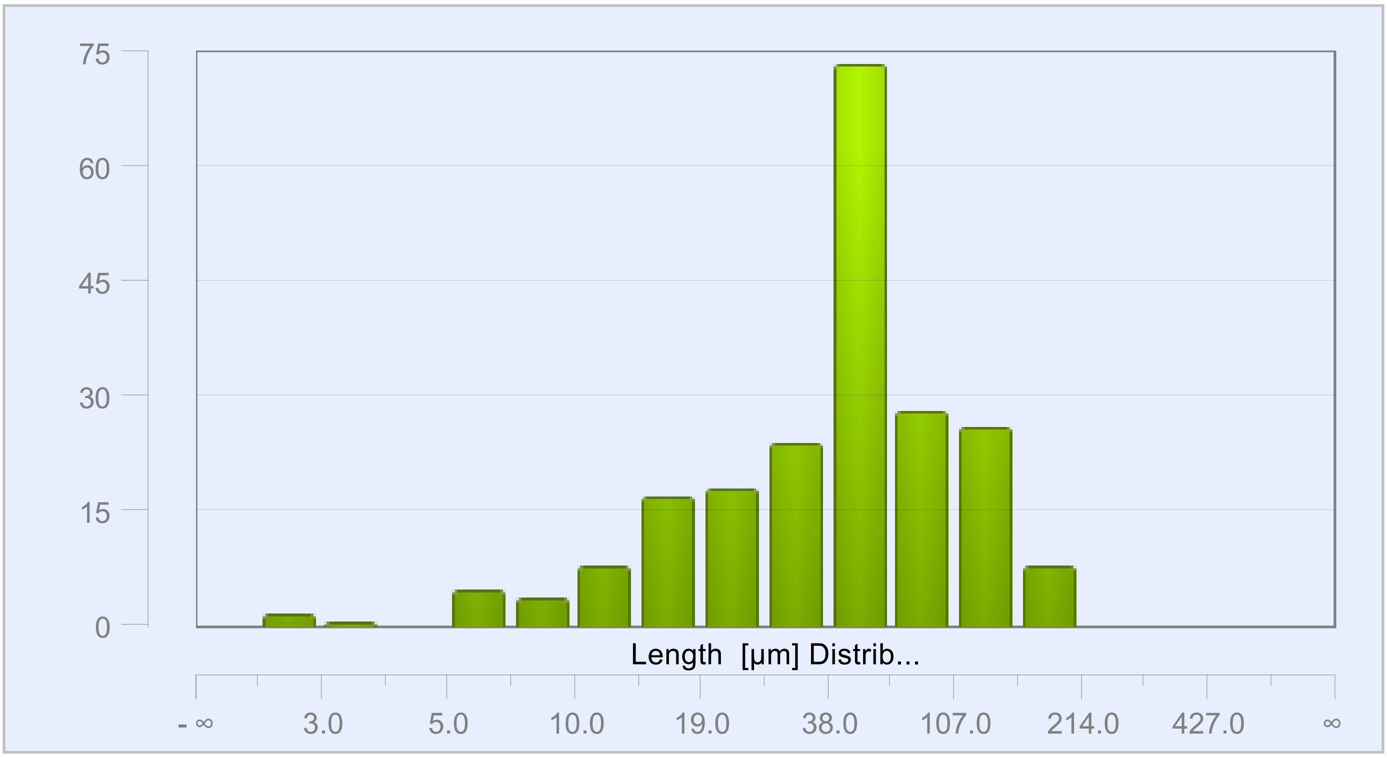

Start	End	Absolute Frequency	Absolute Frequency (accumulated)	Relative Frequency [%]	Relative Frequency (accumulated) [%]
	2.0 µm	0	0	0	0
2.0 µm	3.0 µm	2	2	1	1
3.0 µm	4.0 µm	1	3	0	1
4.0 µm	5.0 µm	0	3	0	1
5.0 µm	7.0 µm	5	8	2	4
7.0 µm	10.0 µm	4	12	2	6
10.0 µm	13.0 µm	8	20	4	9
13.0 µm	19.0 µm	17	37	8	17
19.0 µm	27.0 µm	18	55	8	26
27.0 µm	38.0 µm	24	79	11	37
38.0 µm	75.0 µm	73	152	34	71
75.0 µm	107.0 µm	28	180	13	84

Start	End	Absolute Frequency	Absolute Frequency (accumulated)	Relative Frequency [%]	Relative Frequency (accumulated) [%]
107.0 µm	151.0 µm	26	206	12	96
151.0 µm	214.0 µm	8	214	4	100
214.0 µm	302.0 µm	0	214	0	100
302.0 µm	427.0 µm	0	214	0	100
427.0 µm	600.0 µm	0	214	0	100
600.0 µm		0	214	0	100

4. Single Result 3 (MnFeNi Semesterprojekt_MnFeNi_homogenized_8.1mmSW_1000°C_60min_00062)

Mean chord length	68.5 µm
Grain size (ASTM)	4.4
Grain size (G643)	4.4
Grain stretching	92 %

4.1. Statistical Analysis

Statistical Data	Length
Object Count	184
Minimum	0.4 µm
Maximum	287.0 µm
Average	68.5 µm
Standard deviation	45.0 µm
Skewness	0.0
Standard deviation (n-1)	45.1 µm
Variance	2'021.6 µm²
Variance (n-1)	2'032.7 µm²
Sum	12'610.4 µm
Sum of squares	1'236'231.8 µm²
Sum of cubes	153'693'070.3 µm³

4.1.1. Chord Lenght Distribution

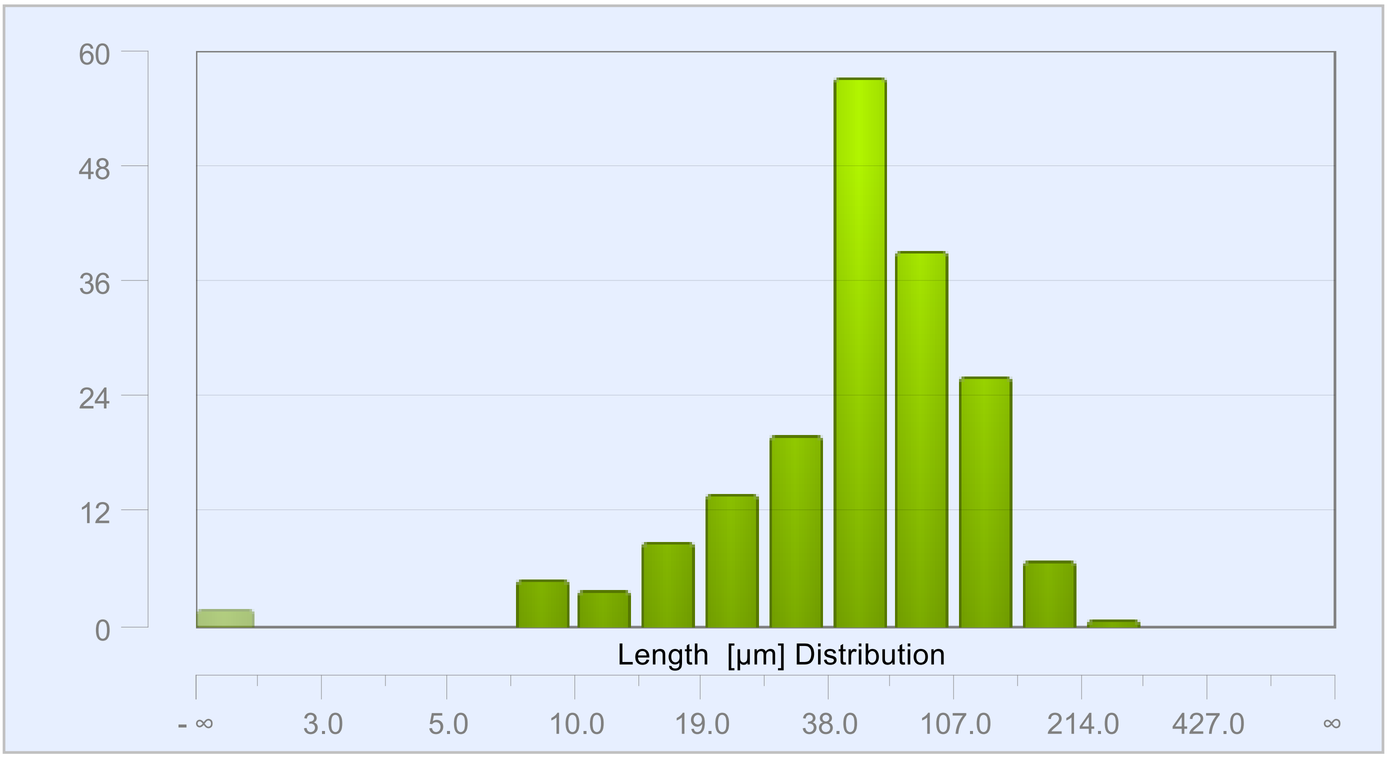

Start	End	Absolute Frequency	Absolute Frequency (accumulated)	Relative Frequency [%]	Relative Frequency (accumulated) [%]
	2.0 µm	2	2	1	1
2.0 µm	3.0 µm	0	2	0	1
3.0 µm	4.0 µm	0	2	0	1
4.0 µm	5.0 µm	0	2	0	1
5.0 µm	7.0 µm	0	2	0	1
7.0 µm	10.0 µm	5	7	3	4
10.0 µm	13.0 µm	4	11	2	6
13.0 µm	19.0 µm	9	20	5	11
19.0 µm	27.0 µm	14	34	8	18
27.0 µm	38.0 µm	20	54	11	29
38.0 µm	75.0 µm	57	111	31	60
75.0 µm	107.0 µm	39	150	21	82
107.0 µm	151.0 µm	26	176	14	96
151.0 µm	214.0 µm	7	183	4	99
214.0 µm	302.0 µm	1	184	1	100
302.0 µm	427.0 µm	0	184	0	100
427.0 µm	600.0 µm	0	184	0	100
600.0 µm		0	184	0	100

5. Single Result 4 (MnFeNi Semesterprojekt_MnFeNi_homogenized_8.1mmSW_1000°C_60min_00063)

Mean chord length	67.5 µm
Grain size (ASTM)	4.5
Grain size (G643)	4.4
Grain stretching	97.1 %

5.1. Statistical Analysis

Statistical Data	Length
Object Count	187
Minimum	0.8 µm
Maximum	228.7 µm
Average	67.5 µm
Standard deviation	46.0 µm
Skewness	0.0
Standard deviation (n-1)	46.1 µm
Variance	2'115.5 µm²
Variance (n-1)	2'126.8 µm²
Sum	12'618.7 µm
Sum of squares	1'247'096.3 µm²
Sum of cubes	155'822'049.6 µm³

5.1.1. Chord Lenght Distribution

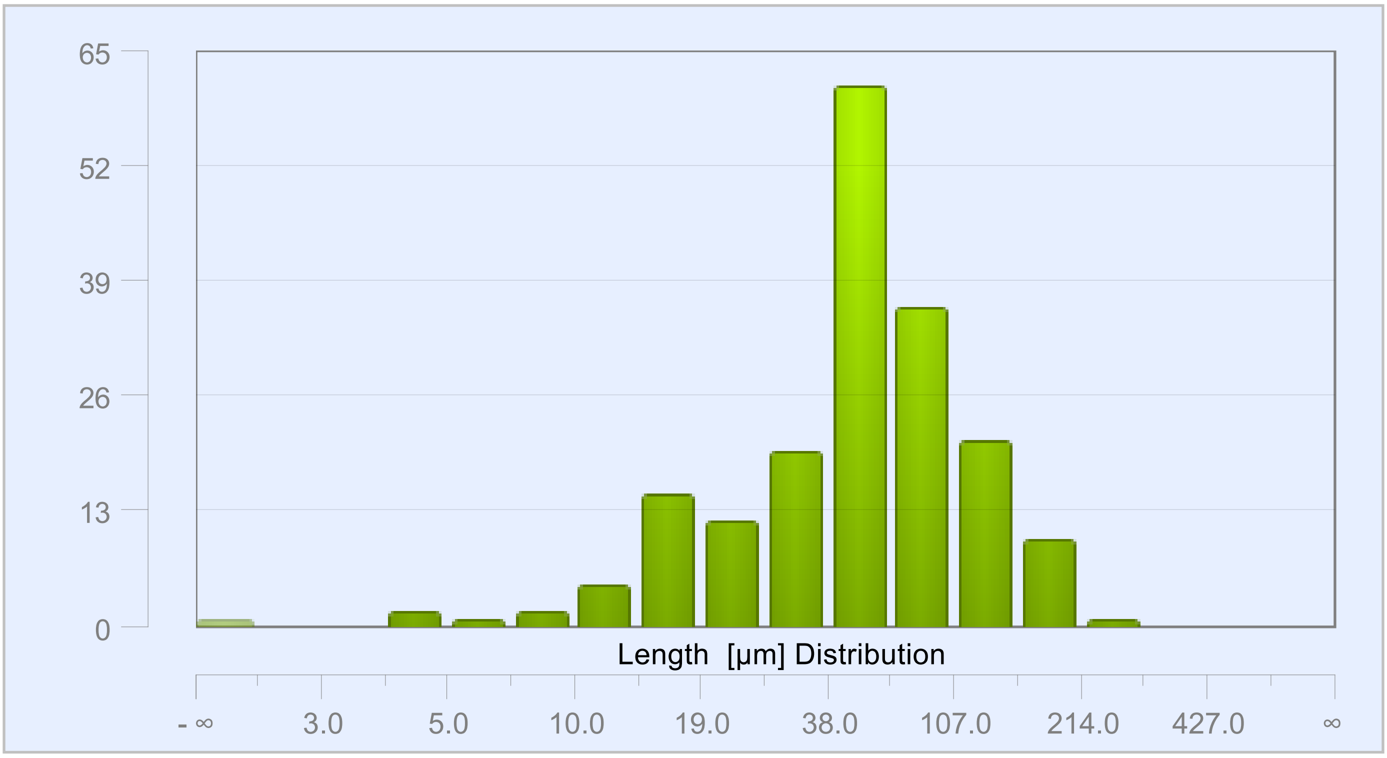

Start	End	Absolute Frequency	Absolute Frequency (accumulated)	Relative Frequency [%]	Relative Frequency (accumulated) [%]
	2.0 µm	1	1	1	1
2.0 µm	3.0 µm	0	1	0	1
3.0 µm	4.0 µm	0	1	0	1
4.0 µm	5.0 µm	2	3	1	2
5.0 µm	7.0 µm	1	4	1	2
7.0 µm	10.0 µm	2	6	1	3
10.0 µm	13.0 µm	5	11	3	6
13.0 µm	19.0 µm	15	26	8	14
19.0 µm	27.0 µm	12	38	6	20
27.0 µm	38.0 µm	20	58	11	31
38.0 µm	75.0 µm	61	119	33	64
75.0 µm	107.0 µm	36	155	19	83
107.0 µm	151.0 µm	21	176	11	94
151.0 µm	214.0 µm	10	186	5	99
214.0 µm	302.0 µm	1	187	1	100
302.0 µm	427.0 µm	0	187	0	100
427.0 µm	600.0 µm	0	187	0	100
600.0 µm		0	187	0	100
