# Supplementary material for: Data compilation on the effect of grain size, temperature, and texture on the strength of a single-phase FCC MnFeNi medium-entropy alloy
Source: Data Brief. 2019 Nov 15;28:104807. doi: 10.1016/j.dib.2019.104807 (PMC6909151; doi:10.1016/j.dib.2019.104807)
Supplement: Multimedia component 1 [file mmc1.zip › MnFeNi_1373K_30min/MnFeNi_1373K_30min_c=86μm.pdf]

Analysebericht

29.05.2018 15:34:33

powered by imagic.ch

1. cumulative Result 1

Anzahl Bilder	1
Korngröße (ASTM)	3,8
Korngröße (G643)	3,8
Kornstreckung	99,1 %
Mittlere Sehnenlänge	85,8 µm

2. Single Result 1 (MnFeNi Semesterprojekt_MnFeNi_homogenized_8.1mmSW_1100°C_30min_00097)

Mittlere Sehnenlänge	85,8 µm
Korngröße (ASTM)	3,8
Korngröße (G643)	3,8
Kornstreckung	99,1 %

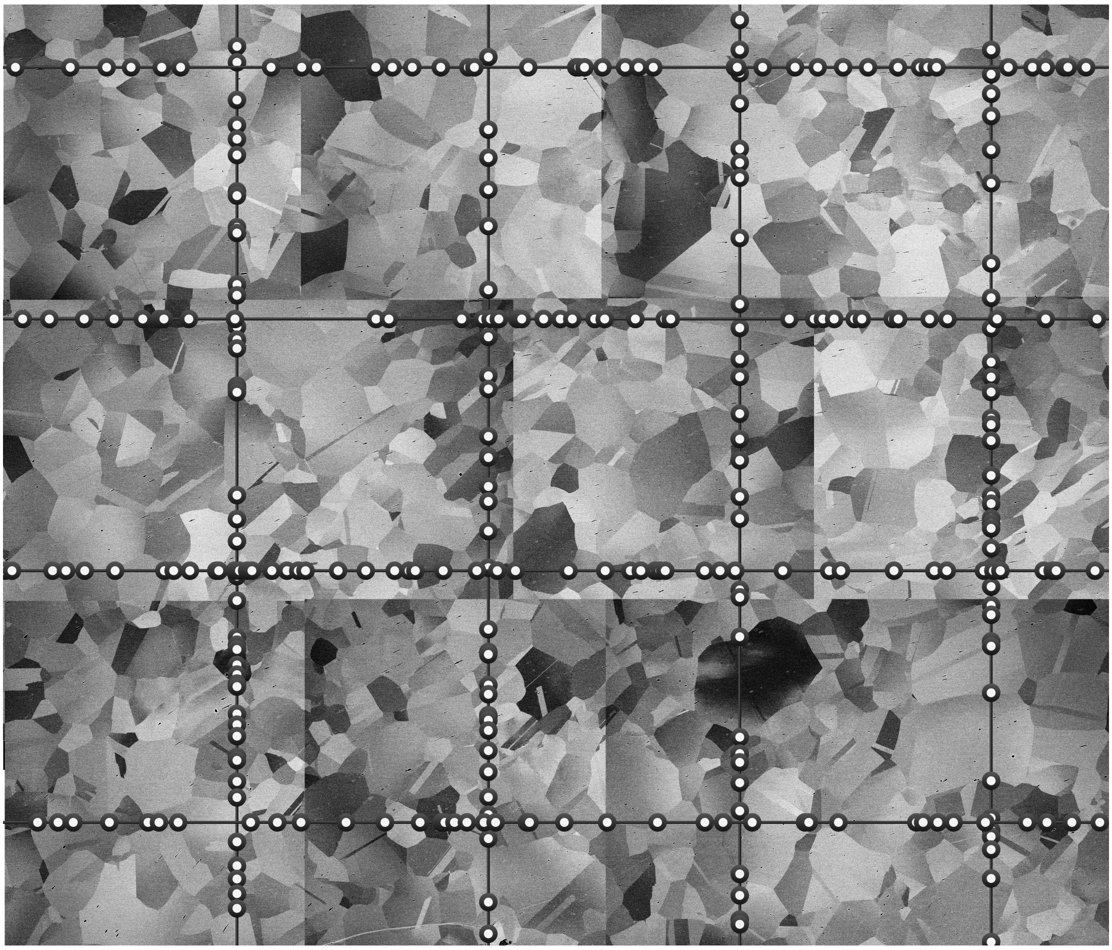

2.1. Statistische Analyse

Statistische Daten	Länge
Anzahl Objekte	317
Minimum	5,0 µm
Maximum	468,5 µm
Mittelwert	85,8 µm
Standardabweichung	66,5 µm
Schiefe	0,0
Standardabweichung (n-1)	66,6 µm
Varianz	4'424,6 µm²
Varianz (n-1)	4'438,6 µm²
Summe	27'195,4 µm
Quadratsumme	3'735'688,7 µm²

Statistische Daten	Länge
Kubiksumme	737'284'026,8 µm³

2.1.1. Chord Lenght Distribution

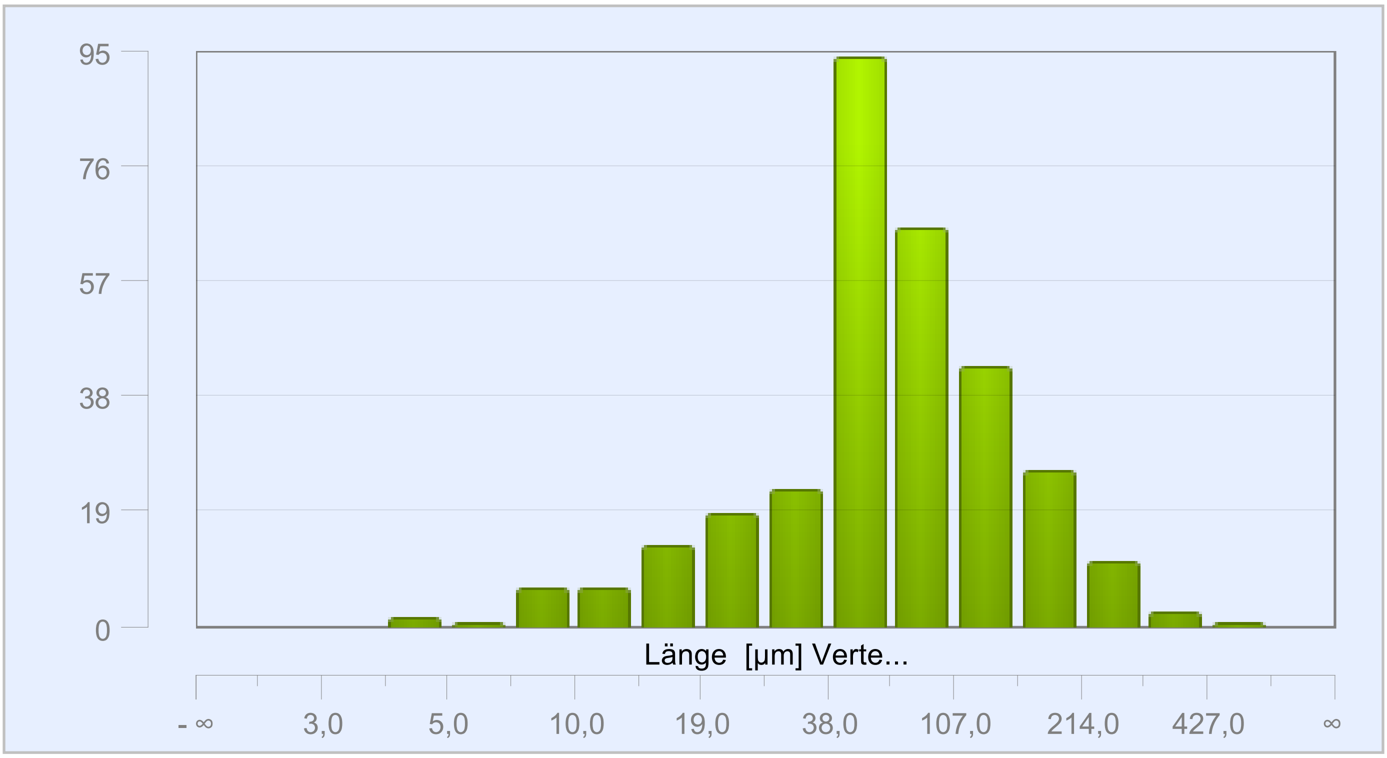

Start	Ende	Absolute Häufigkeit	Absolute Häufigkeit (kumuliert)	Relative Häufigkeit [%]	Relative Häufigkeit (kumuliert) [%]
	2,0 µm	0	0	0	0
2,0 µm	3,0 µm	0	0	0	0
3,0 µm	4,0 µm	0	0	0	0
4,0 µm	5,0 µm	2	2	1	1
5,0 µm	7,0 µm	1	3	0	1
7,0 µm	10,0 µm	7	10	2	3
10,0 µm	13,0 µm	7	17	2	5
13,0 µm	19,0 µm	14	31	4	10
19,0 µm	27,0 µm	19	50	6	16
27,0 µm	38,0 µm	23	73	7	23
38,0 µm	75,0 µm	94	167	30	53
75,0 µm	107,0 µm	66	233	21	74
107,0 µm	151,0 µm	43	276	14	87
151,0 µm	214,0 µm	26	302	8	95
214,0 µm	302,0 µm	11	313	3	99
302,0 µm	427,0 µm	3	316	1	100
427,0 µm	600,0 µm	1	317	0	100
600,0 µm		0	317	0	100
