# Supplementary material for: Data compilation on the effect of grain size, temperature, and texture on the strength of a single-phase FCC MnFeNi medium-entropy alloy
Source: Data Brief. 2019 Nov 15;28:104807. doi: 10.1016/j.dib.2019.104807 (PMC6909151; doi:10.1016/j.dib.2019.104807)
Supplement: Multimedia component 1 [file mmc1.zip › MnFeNi_1373K_30min/MnFeNi_1373K_30min_d=112μm.pdf]

Analysebericht

29.05.2018 15:21:33

powered by imagic.ch

1. cumulative Result 1

Anzahl Bilder	1
Korngröße (ASTM)	3
Korngröße (G643)	3
Kornstreckung	99,5 %
Mittlere Sehnenlänge	111,9 µm

2. Single Result 1 (MnFeNi Semesterprojekt_MnFeNi_homogenized_8.1mmSW_1100°C_30min_00097)

Mittlere Sehnenlänge	111,9 µm
Korngröße (ASTM)	3
Korngröße (G643)	3
Kornstreckung	99,5 %

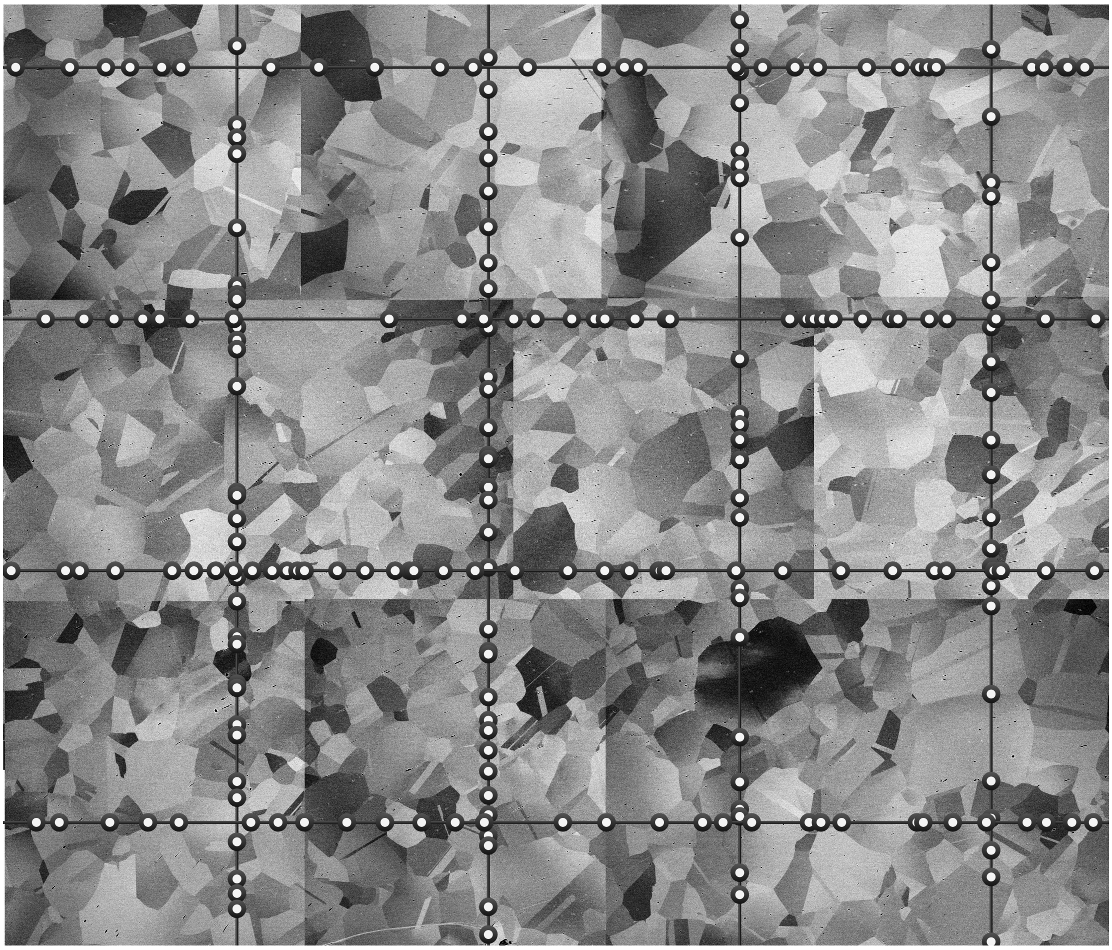

2.1. Statistische Analyse

Statistische Daten	Länge
Anzahl Objekte	243
Minimum	5,0 µm
Maximum	516,6 µm
Mittelwert	111,9 µm
Standardabweichung	77,0 µm
Schiefe	0,0
Standardabweichung (n-1)	77,2 µm
Varianz	5'933,2 µm²
Varianz (n-1)	5'957,7 µm²
Summe	27'195,4 µm
Quadratsumme	4'485'337,8 µm²

Statistische Daten	Länge
Kubiksumme	1'003'346'048,6 µm³

2.1.1. Chord Lenght Distribution

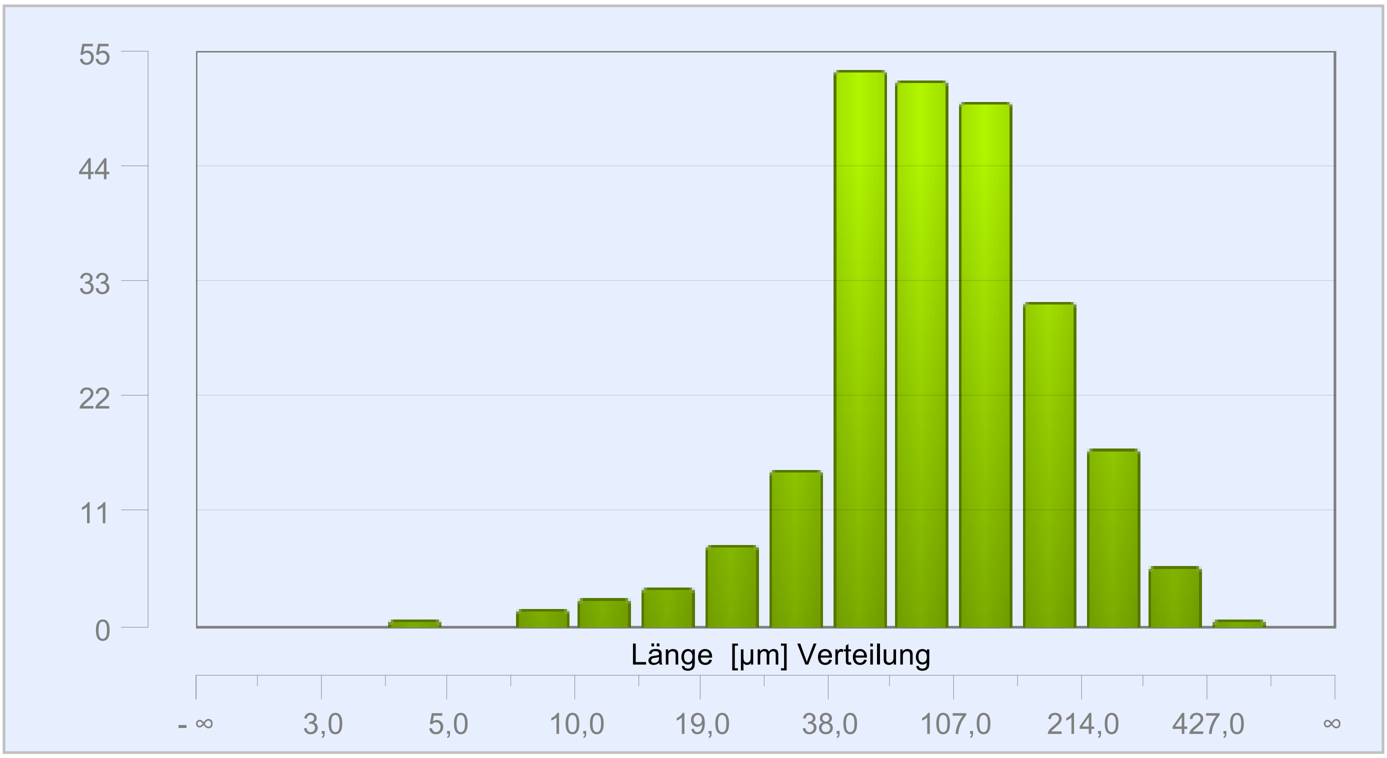

Start	Ende	Absolute Häufigkeit	Absolute Häufigkeit (kumuliert)	Relative Häufigkeit [%]	Relative Häufigkeit (kumuliert) [%]
	2,0 µm	0	0	0	0
2,0 µm	3,0 µm	0	0	0	0
3,0 µm	4,0 µm	0	0	0	0
4,0 µm	5,0 µm	1	1	0	0
5,0 µm	7,0 µm	0	1	0	0
7,0 µm	10,0 µm	2	3	1	1
10,0 µm	13,0 µm	3	6	1	2
13,0 µm	19,0 µm	4	10	2	4
19,0 µm	27,0 µm	8	18	3	7
27,0 µm	38,0 µm	15	33	6	14
38,0 µm	75,0 µm	53	86	22	35
75,0 µm	107,0 µm	52	138	21	57
107,0 µm	151,0 µm	50	188	21	77
151,0 µm	214,0 µm	31	219	13	90
214,0 µm	302,0 µm	17	236	7	97
302,0 µm	427,0 µm	6	242	2	100
427,0 µm	600,0 µm	1	243	0	100
600,0 µm		0	243	0	100
