# Supplementary material for: Data compilation on the effect of grain size, temperature, and texture on the strength of a single-phase FCC MnFeNi medium-entropy alloy
Source: Data Brief. 2019 Nov 15;28:104807. doi: 10.1016/j.dib.2019.104807 (PMC6909151; doi:10.1016/j.dib.2019.104807)
Supplement: Multimedia component 1 [file mmc1.zip › MnFeNi_1373K_60min/MnFeNi_1373K_60min_c=106μm.pdf]

Analysebericht

30.04.2018 13:21:27

powered by imagic.ch

1. cumulative Result 1

Anzahl Bilder	1
Korngröße (ASTM)	3,2
Korngröße (G643)	3,1
Kornstreckung	90,3 %
Mittlere Sehnenlänge	105,8 µm

2. Single Result 1 (MnFeNi Semesterprojekt_MnFeNi_homogenized_8.1mmSW_1100°C_60min_00082)

Mittlere Sehnenlänge	105,8 µm
Korngröße (ASTM)	3,2
Korngröße (G643)	3,1
Kornstreckung	90,3 %

2.1. Statistische Analyse

Statistische Daten	Länge
Anzahl Objekte	271
Minimum	4,1 µm
Maximum	554,5 µm
Mittelwert	105,8 µm
Standardabweichung	86,6 µm
Schiefe	0,0
Standardabweichung (n-1)	86,8 µm
Varianz	7'503,2 µm²
Varianz (n-1)	7'531,0 µm²
Summe	28'661,2 µm
Quadratsumme	5'064'598,0 µm²
Kubiksumme	1'249'378'563,9 µm³

2.1.1. Chord Lenght Distribution

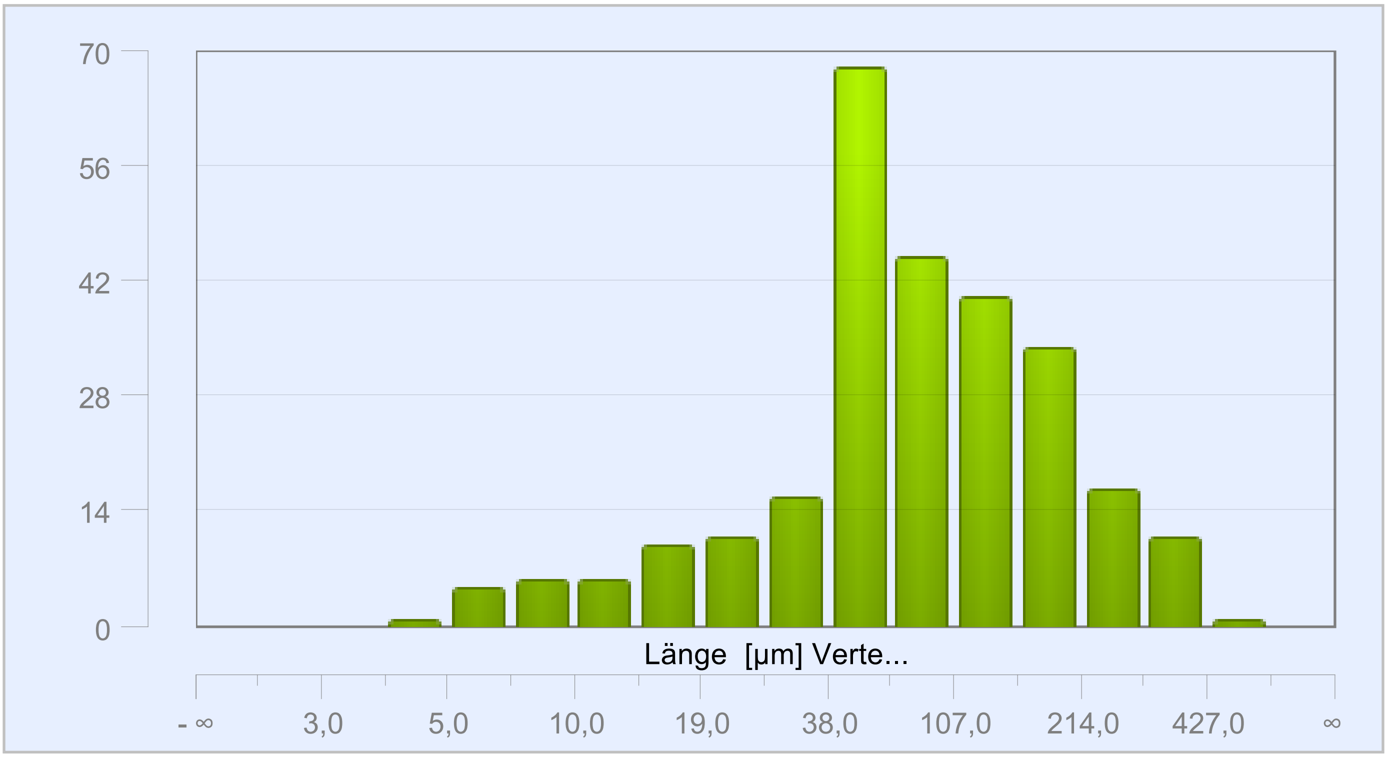

Start	Ende	Absolute Häufigkeit	Absolute Häufigkeit (kumuliert)	Relative Häufigkeit [%]	Relative Häufigkeit (kumuliert) [%]
	2,0 µm	0	0	0	0

Start	Ende	Absolute Häufigkeit	Absolute Häufigkeit (kumuliert)	Relative Häufigkeit [%]	Relative Häufigkeit (kumuliert) [%]
2,0 µm	3,0 µm	0	0	0	0
3,0 µm	4,0 µm	0	0	0	0
4,0 µm	5,0 µm	1	1	0	0
5,0 µm	7,0 µm	5	6	2	2
7,0 µm	10,0 µm	6	12	2	4
10,0 µm	13,0 µm	6	18	2	7
13,0 µm	19,0 µm	10	28	4	10
19,0 µm	27,0 µm	11	39	4	14
27,0 µm	38,0 µm	16	55	6	20
38,0 µm	75,0 µm	68	123	25	45
75,0 µm	107,0 µm	45	168	17	62
107,0 µm	151,0 µm	40	208	15	77
151,0 µm	214,0 µm	34	242	13	89
214,0 µm	302,0 µm	17	259	6	96
302,0 µm	427,0 µm	11	270	4	100
427,0 µm	600,0 µm	1	271	0	100
600,0 µm		0	271	0	100
