# Supplementary material for: Data compilation on the effect of grain size, temperature, and texture on the strength of a single-phase FCC MnFeNi medium-entropy alloy
Source: Data Brief. 2019 Nov 15;28:104807. doi: 10.1016/j.dib.2019.104807 (PMC6909151; doi:10.1016/j.dib.2019.104807)
Supplement: Multimedia component 1 [file mmc1.zip › MnFeNi_1373K_60min/MnFeNi_1373K_60min_d=167μm.pdf]

Analysebericht

02.05.2018 12:06:03

powered by imagic.ch

1. cumulative Result 1

Anzahl Bilder	1
Korngröße (ASTM)	1,9
Korngröße (G643)	1,8
Kornstreckung	96,2 %
Mittlere Sehnenlänge	166,6 µm

2. Single Result 1 (MnFeNi Semesterprojekt_MnFeNi_homogenized_8.1mmSW_1100°C_60min_00082)

Mittlere Sehnenlänge	166,6 µm
Korngröße (ASTM)	1,9
Korngröße (G643)	1,8
Kornstreckung	96,2 %

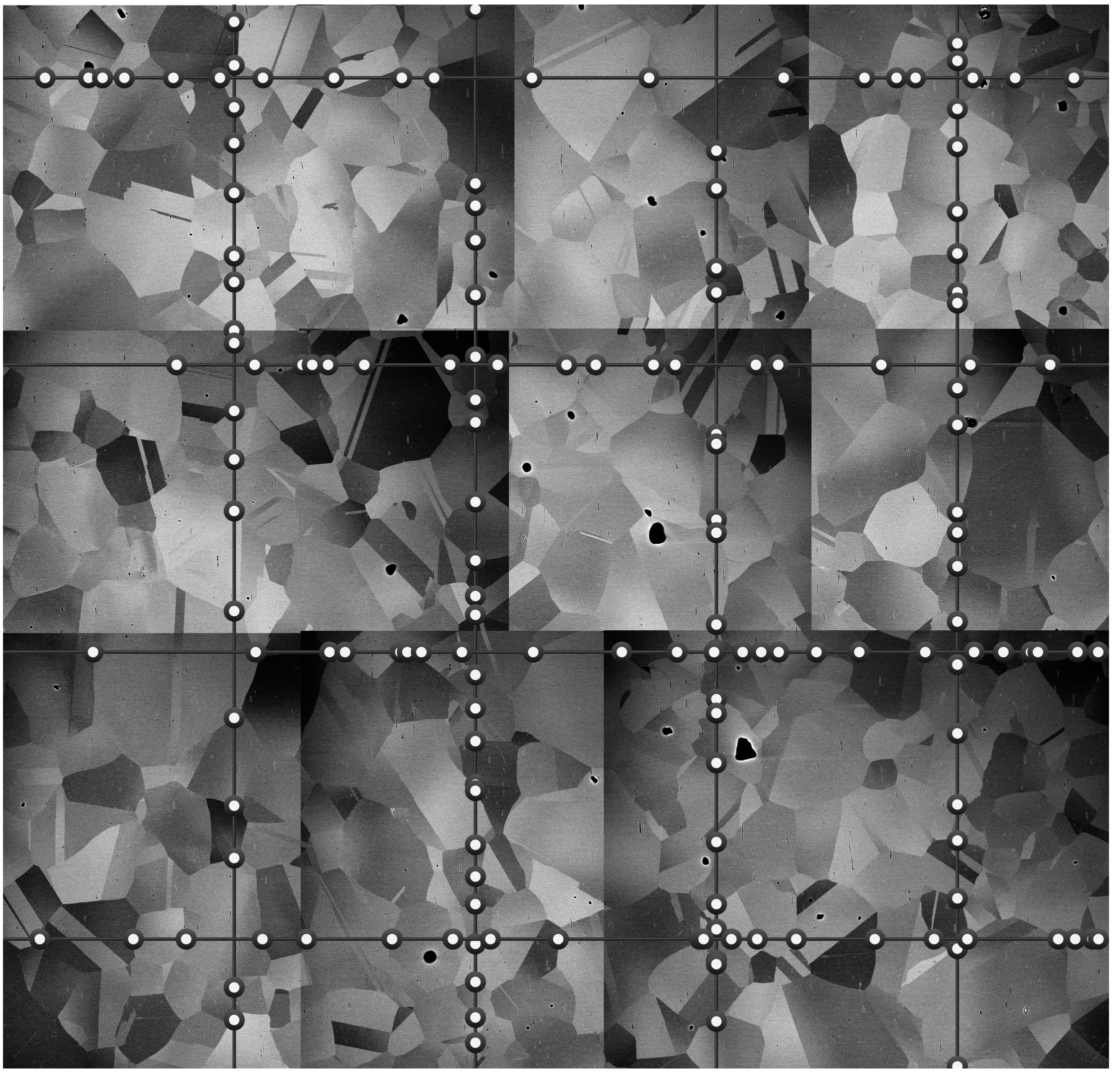

2.1. Statistische Analyse

Statistische Daten	Länge
Anzahl Objekte	172
Minimum	5,8 µm
Maximum	575,2 µm
Mittelwert	166,6 µm
Standardabweichung	111,4 µm
Schiefe	0,0
Standardabweichung (n-1)	111,7 µm
Varianz	12'414,9 µm²

Statistische Daten	Länge
Varianz (n-1)	12'487,5 µm²
Summe	28'661,2 µm
Quadratsumme	6'911'301,1 µm²
Kubiksumme	2'169'456'571,9 µm³

2.1.1. Chord Lenght Distribution

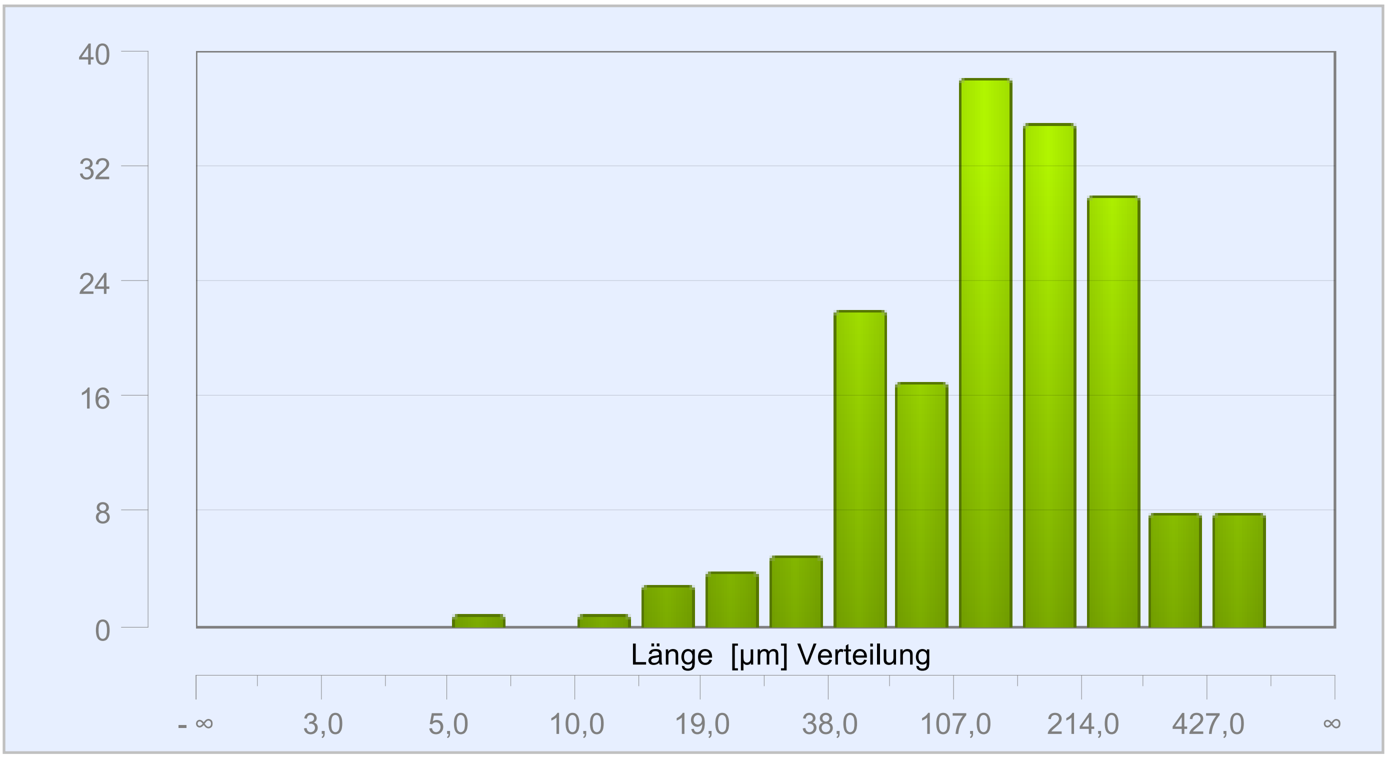

Start	Ende	Absolute Häufigkeit	Absolute Häufigkeit (kumuliert)	Relative Häufigkeit [%]	Relative Häufigkeit (kumuliert) [%]
	2,0 µm	0	0	0	0
2,0 µm	3,0 µm	0	0	0	0
3,0 µm	4,0 µm	0	0	0	0
4,0 µm	5,0 µm	0	0	0	0
5,0 µm	7,0 µm	1	1	1	1
7,0 µm	10,0 µm	0	1	0	1
10,0 µm	13,0 µm	1	2	1	1
13,0 µm	19,0 µm	3	5	2	3
19,0 µm	27,0 µm	4	9	2	5
27,0 µm	38,0 µm	5	14	3	8
38,0 µm	75,0 µm	22	36	13	21
75,0 µm	107,0 µm	17	53	10	31
107,0 µm	151,0 µm	38	91	22	53
151,0 µm	214,0 µm	35	126	20	73
214,0 µm	302,0 µm	30	156	17	91
302,0 µm	427,0 µm	8	164	5	95
427,0 µm	600,0 µm	8	172	5	100
600,0 µm		0	172	0	100
