# Supplementary material for: Data compilation on the effect of grain size, temperature, and texture on the strength of a single-phase FCC MnFeNi medium-entropy alloy
Source: Data Brief. 2019 Nov 15;28:104807. doi: 10.1016/j.dib.2019.104807 (PMC6909151; doi:10.1016/j.dib.2019.104807)
Supplement: Multimedia component 1 [file mmc1.zip › MnFeNi_1473K_60min/MnFeNi_1473K_60min_c=120μm.pdf]

Analysebericht

Mar 2, 2018 3:45:01 PM

powered by imagic.ch

1. cumulative Result 1

Number of images	1
Grain size (ASTM)	2.8
Grain size (G643)	2.8
Grain stretching	68.9 %
Mean chord length	120.1 µm

2. Single Result 1 (MnFeNi Semesterprojekt_MnFeNi_homogenized_8.1mmSW_1200_60min_00044)

Mean chord length	120.1 µm
Grain size (ASTM)	2.8
Grain size (G643)	2.8
Grain stretching	68.9 %

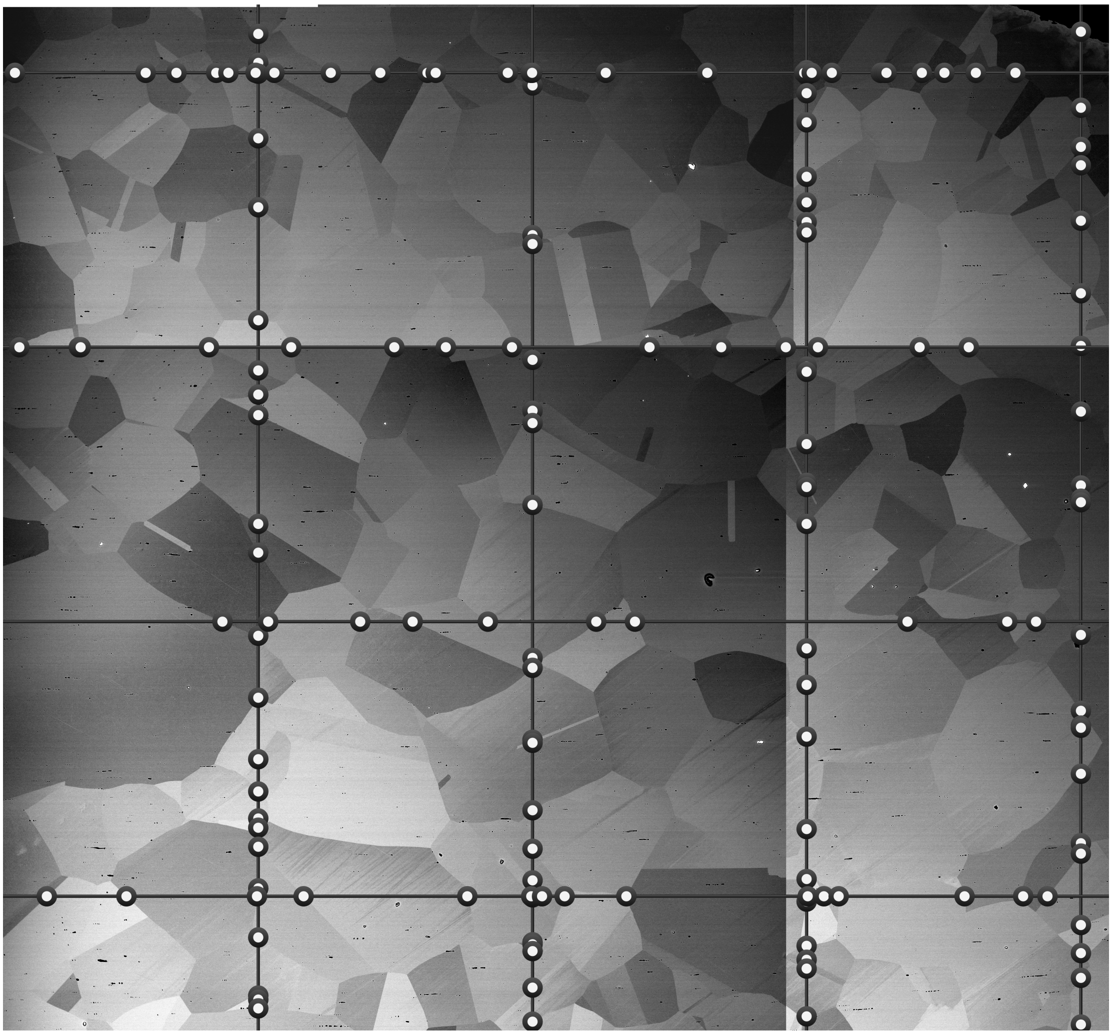

2.1. Statistical Analysis

Statistical Data	Length
Object Count	162
Minimum	3.0 µm
Maximum	622.0 µm
Average	120.1 µm
Standard deviation	101.6 µm
Skewness	0.0
Standard deviation (n-1)	101.9 µm
Variance	10'327.8 µm²
Variance (n-1)	10'392.0 µm²

Statistical Data	Length
Sum	19'463.8 µm
Sum of squares	4'011'622.8 µm²
Sum of cubes	1'158'100'903.0 µm³

2.1.1. Chord Lenght Distribution

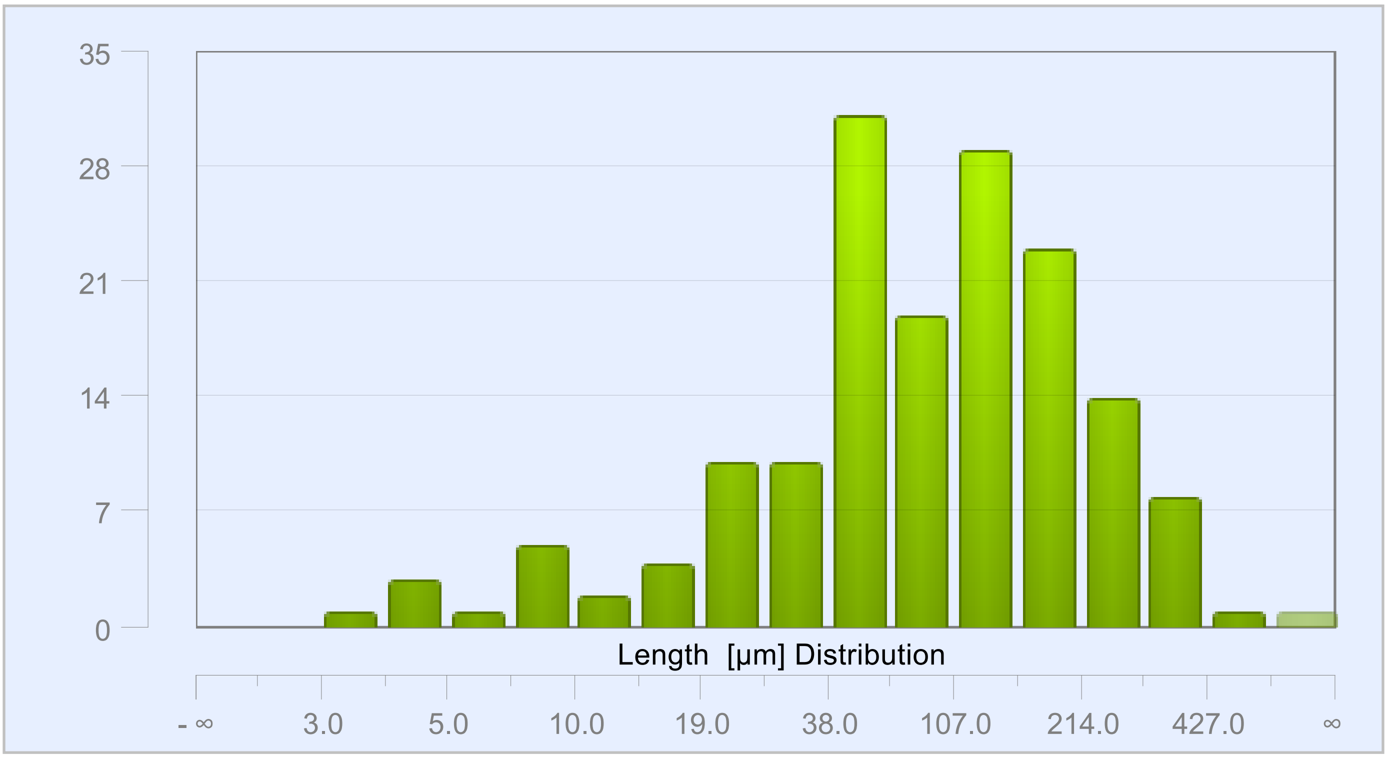

Start	End	Absolute Frequency	Absolute Frequency (accumulated)	Relative Frequency [%]	Relative Frequency (accumulated) [%]
	2.0 µm	0	0	0	0
2.0 µm	3.0 µm	0	0	0	0
3.0 µm	4.0 µm	1	1	1	1
4.0 µm	5.0 µm	3	4	2	2
5.0 µm	7.0 µm	1	5	1	3
7.0 µm	10.0 µm	5	10	3	6
10.0 µm	13.0 µm	2	12	1	7
13.0 µm	19.0 µm	4	16	2	10
19.0 µm	27.0 µm	10	26	6	16
27.0 µm	38.0 µm	10	36	6	22
38.0 µm	75.0 µm	31	67	19	41
75.0 µm	107.0 µm	19	86	12	53
107.0 µm	151.0 µm	29	115	18	71
151.0 µm	214.0 µm	23	138	14	85
214.0 µm	302.0 µm	14	152	9	94
302.0 µm	427.0 µm	8	160	5	99
427.0 µm	600.0 µm	1	161	1	99
600.0 µm		1	162	1	100
