# Supplementary material for: Data compilation on the effect of grain size, temperature, and texture on the strength of a single-phase FCC MnFeNi medium-entropy alloy
Source: Data Brief. 2019 Nov 15;28:104807. doi: 10.1016/j.dib.2019.104807 (PMC6909151; doi:10.1016/j.dib.2019.104807)
Supplement: Multimedia component 1 [file mmc1.zip › MnFeNi_1473K_60min/MnFeNi_1473K_60min_d=216μm.pdf]

Analysebericht

Mar 1, 2018 4:21:55 PM

powered by imagic.ch

1. cumulative Result 1

Number of images	1
Grain size (ASTM)	1.1
Grain size (G643)	1.1
Grain stretching	92.8 %
Mean chord length	216.3 µm

2. Single Result 1 (MnFeNi Semesterprojekt_MnFeNi_homogenized_8.1mmSW_1200_60min_00044)

Mean chord length	216.3 µm
Grain size (ASTM)	1.1
Grain size (G643)	1.1
Grain stretching	92.8 %

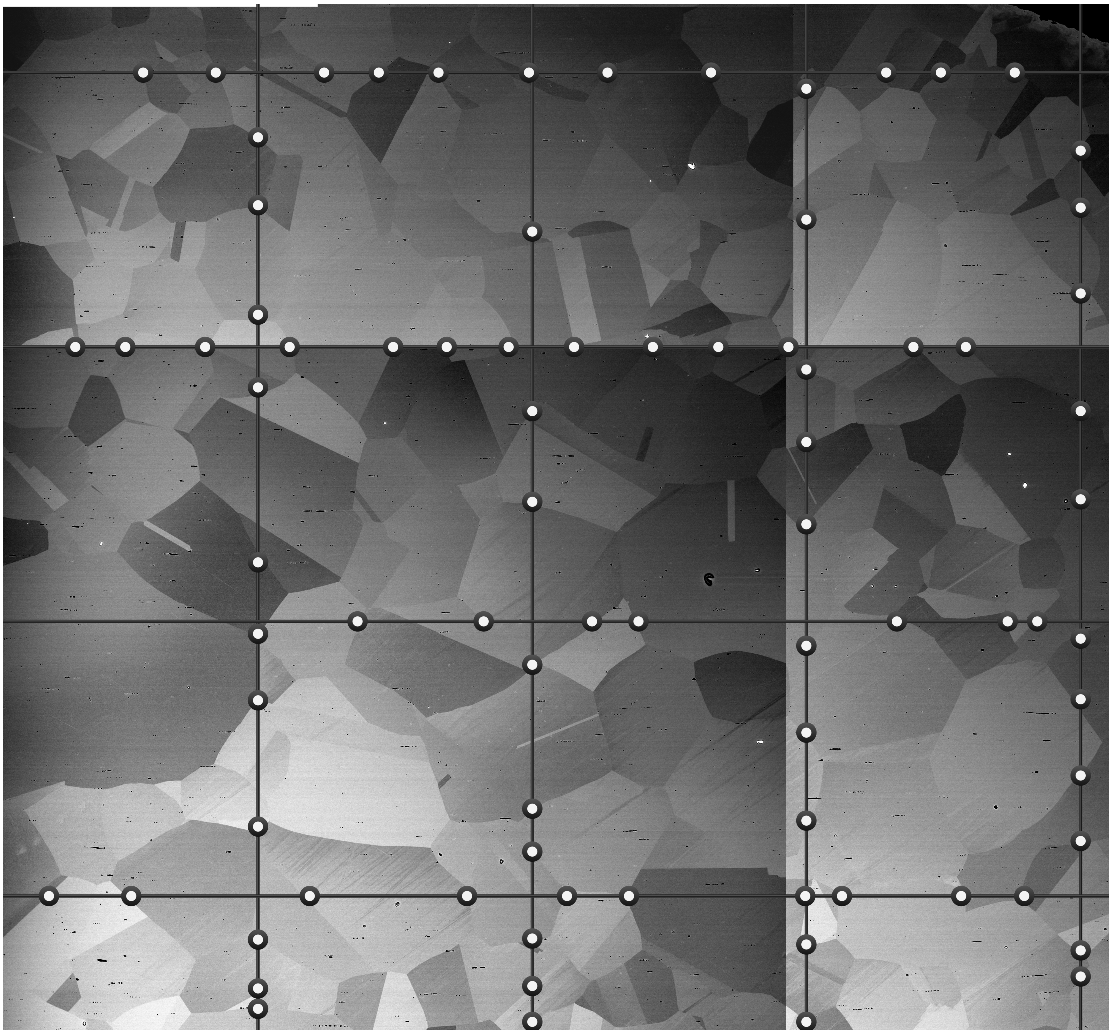

2.1. Statistical Analysis

Statistical Data	Length
Object Count	90
Minimum	18.7 µm
Maximum	809.8 µm
Average	216.3 µm
Standard deviation	122.7 µm
Skewness	0.0
Standard deviation (n-1)	123.4 µm
Variance	15'056.8 µm²
Variance (n-1)	15'226.0 µm²

Statistical Data	Length
Sum	19'463.8 µm
Sum of squares	5'564'445.5 µm²
Sum of cubes	2'075'561'863.0 µm³

2.1.1. Chord Lenght Distribution

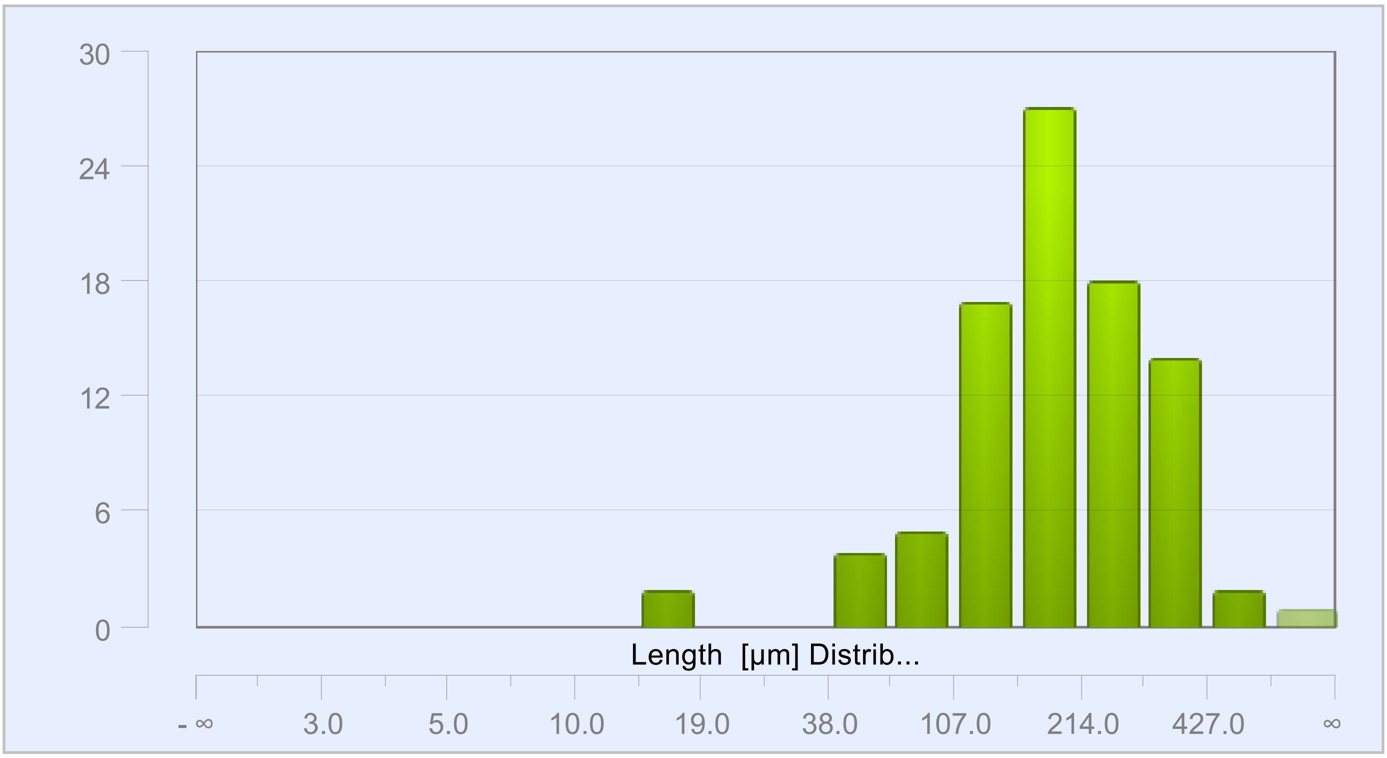

Start	End	Absolute Frequency	Absolute Frequency (accumulated)	Relative Frequency [%]	Relative Frequency (accumulated) [%]
	2.0 µm	0	0	0	0
2.0 µm	3.0 µm	0	0	0	0
3.0 µm	4.0 µm	0	0	0	0
4.0 µm	5.0 µm	0	0	0	0
5.0 µm	7.0 µm	0	0	0	0
7.0 µm	10.0 µm	0	0	0	0
10.0 µm	13.0 µm	0	0	0	0
13.0 µm	19.0 µm	2	2	2	2
19.0 µm	27.0 µm	0	2	0	2
27.0 µm	38.0 µm	0	2	0	2
38.0 µm	75.0 µm	4	6	4	7
75.0 µm	107.0 µm	5	11	6	12
107.0 µm	151.0 µm	17	28	19	31
151.0 µm	214.0 µm	27	55	30	61
214.0 µm	302.0 µm	18	73	20	81
302.0 µm	427.0 µm	14	87	16	97
427.0 µm	600.0 µm	2	89	2	99
600.0 µm		1	90	1	100
