# Supplementary figures and images for: Data compilation on the effect of grain size, temperature, and texture on the strength of a single-phase FCC MnFeNi medium-entropy alloy
Source: Data Brief. 2019 Nov 15;28:104807. doi: 10.1016/j.dib.2019.104807 (PMC6909151; doi:10.1016/j.dib.2019.104807)

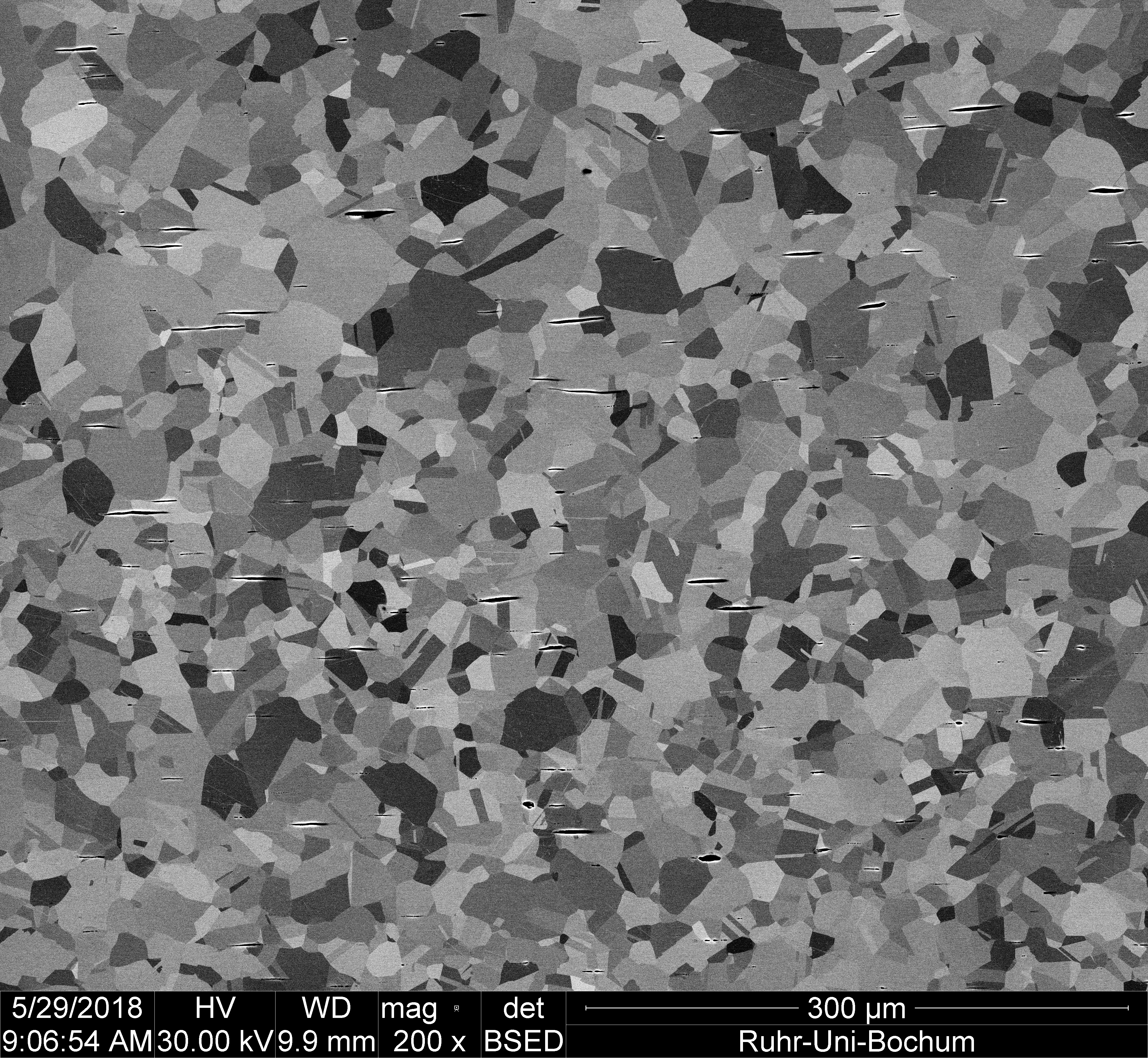

Supplement: Multimedia component 1 [file mmc1.zip › MnFeNi_1073K_120min/MnFeNi_1073K_120min_1.tif]

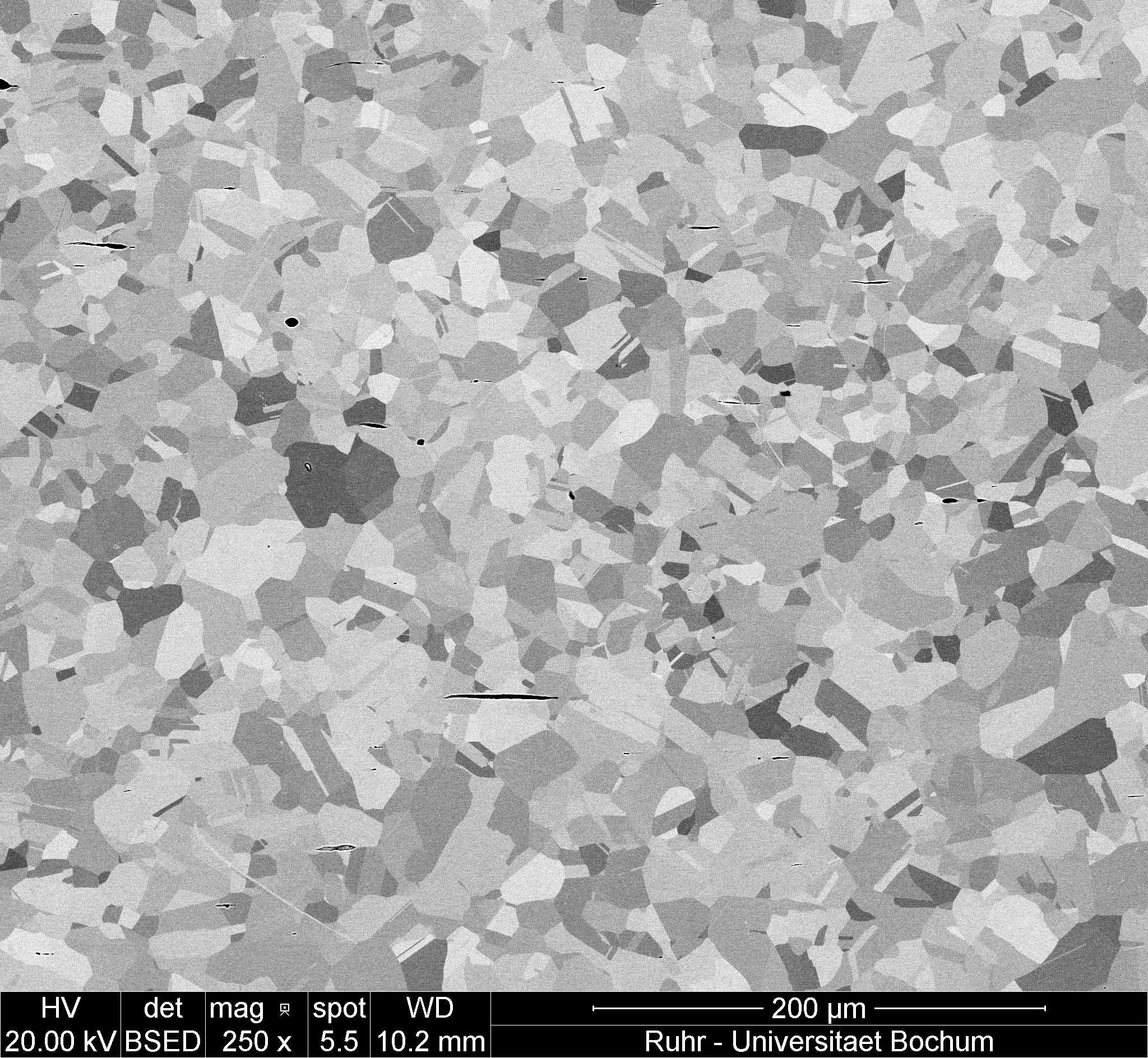

Supplement: Multimedia component 1 [file mmc1.zip › MnFeNi_1073K_45min/MnFeNi_1073K_45min_1.tif]

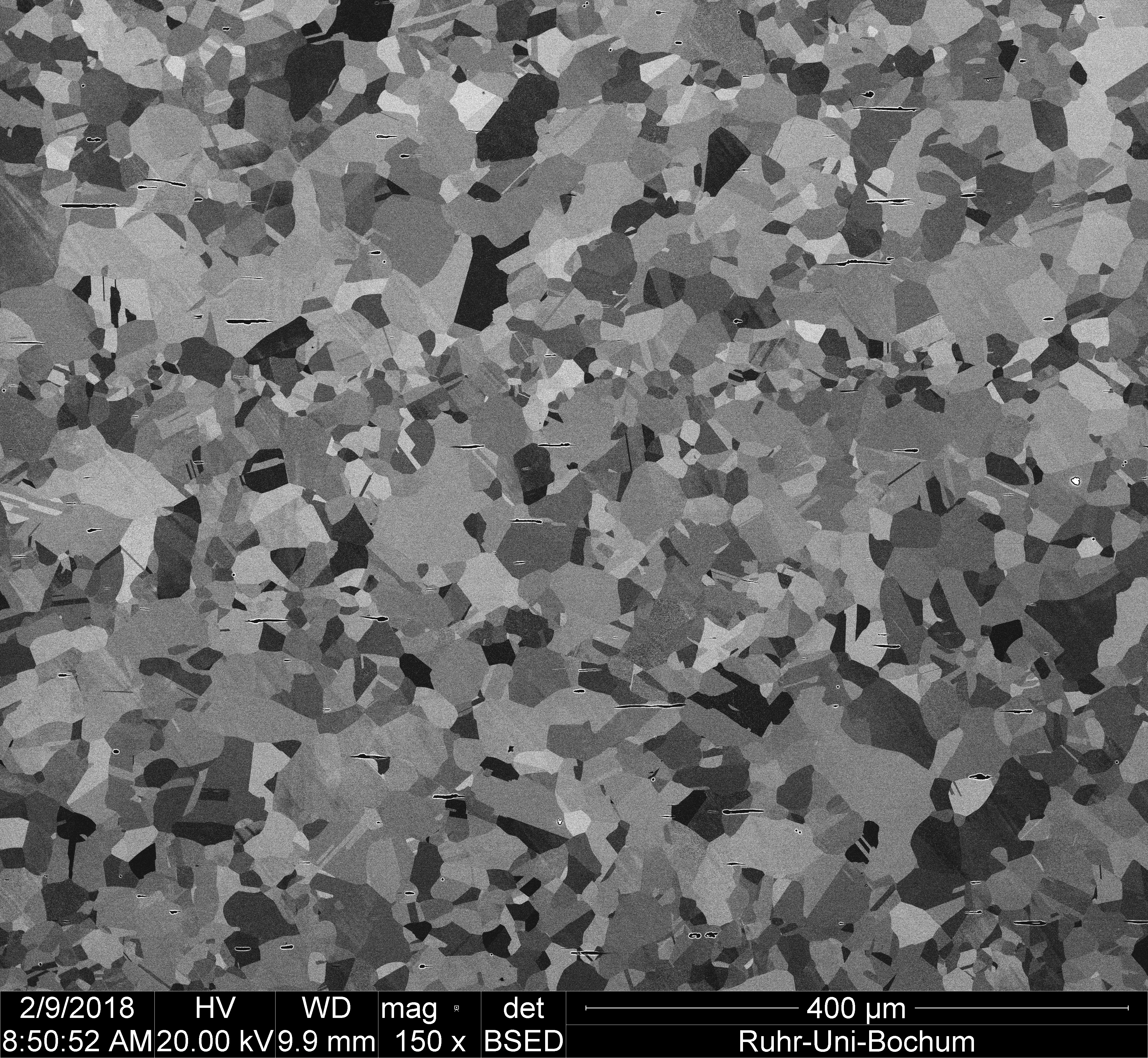

Supplement: Multimedia component 1 [file mmc1.zip › MnFeNi_1073K_60min/MnFeNi_1073K_60min_1.tif]

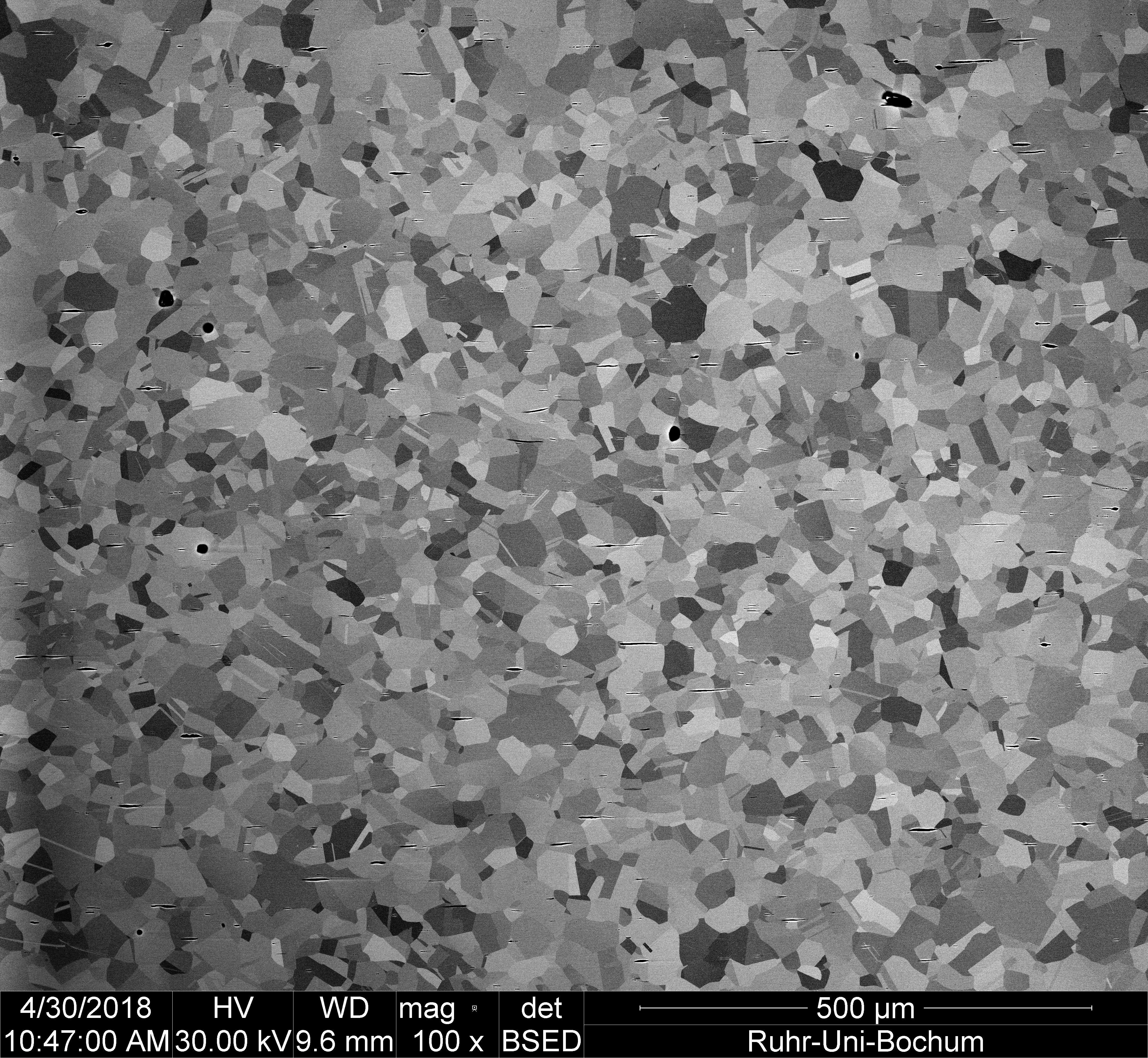

Supplement: Multimedia component 1 [file mmc1.zip › MnFeNi_1173K_30min/MnFeNi_1173K_30min_1.tif]

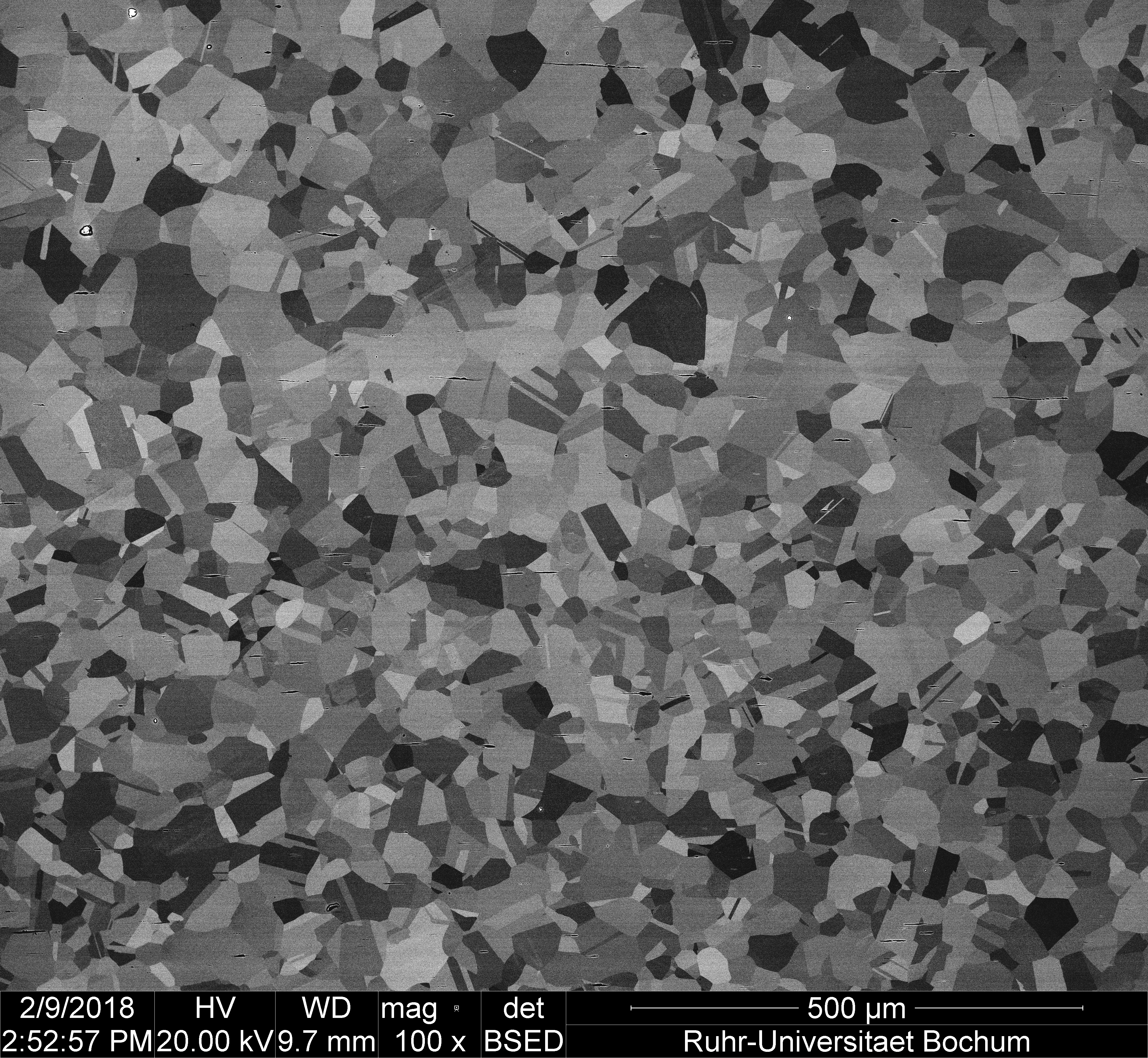

Supplement: Multimedia component 1 [file mmc1.zip › MnFeNi_1173K_60min/MnFeNi_1173K_60min_1.tif]

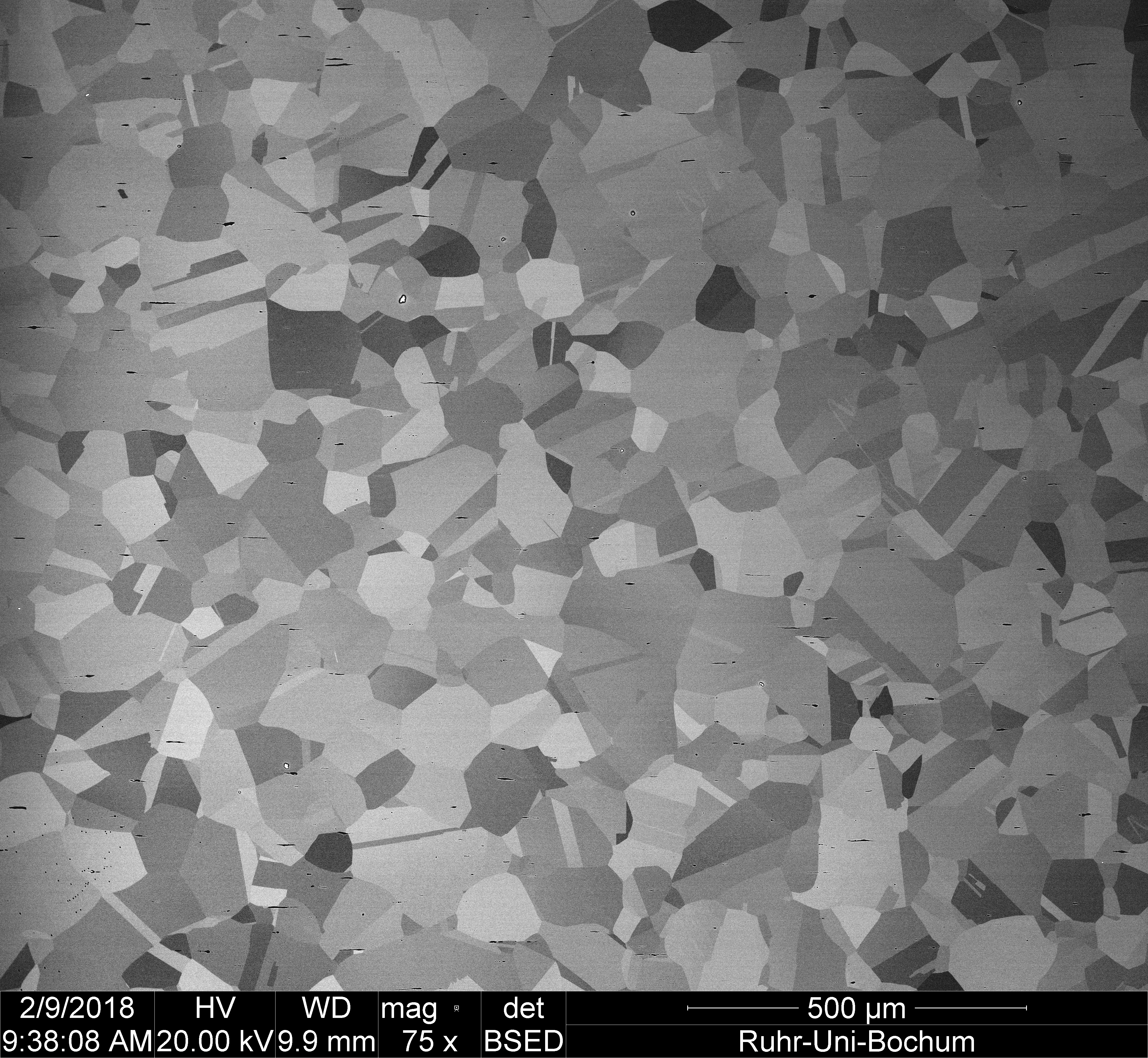

Supplement: Multimedia component 1 [file mmc1.zip › MnFeNi_1273K_60min/MnFeNi_1273K_60min_1.tif]

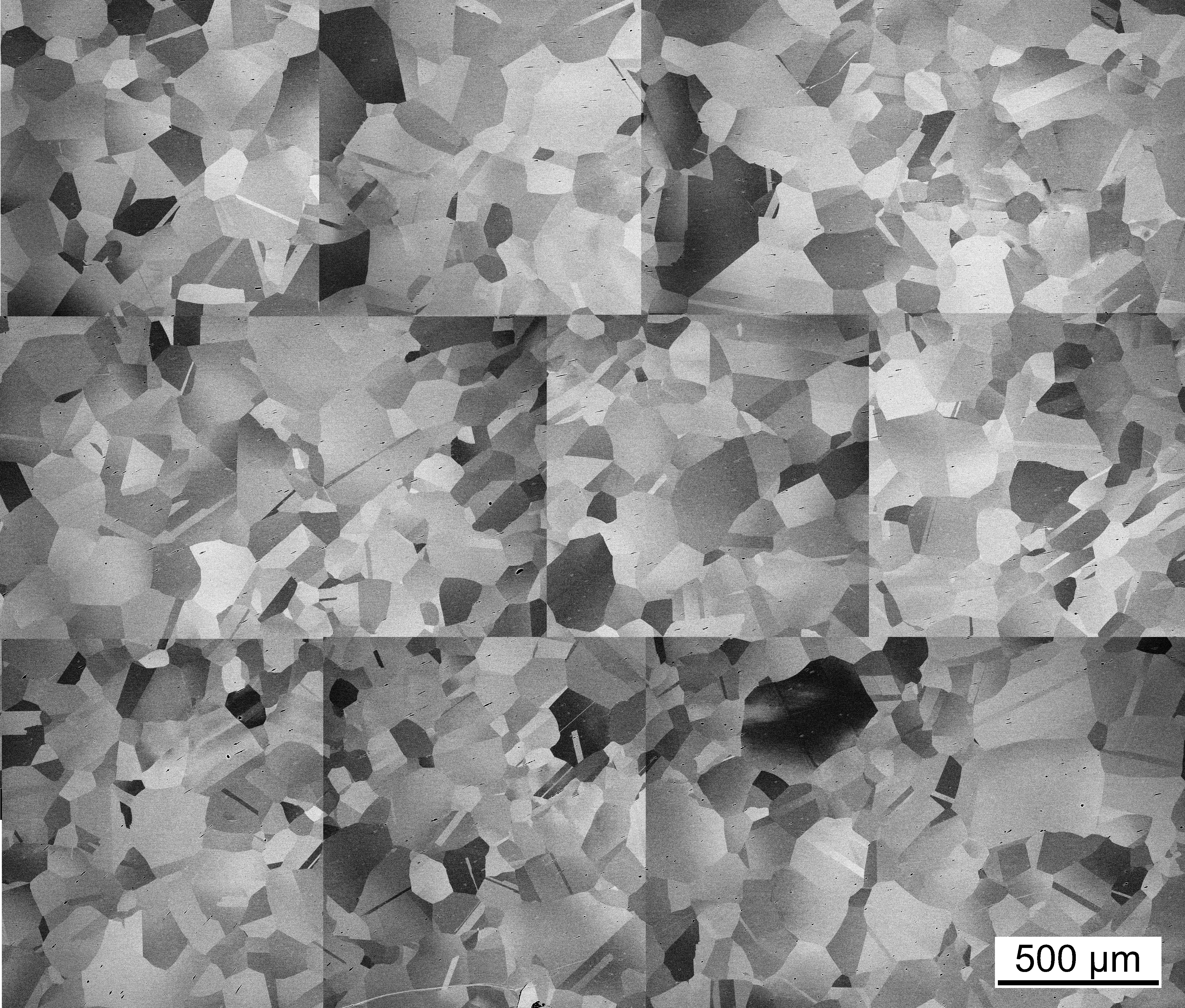

Supplement: Multimedia component 1 [file mmc1.zip › MnFeNi_1373K_30min/MnFeNi_1373K_30min_Montage.jpg]

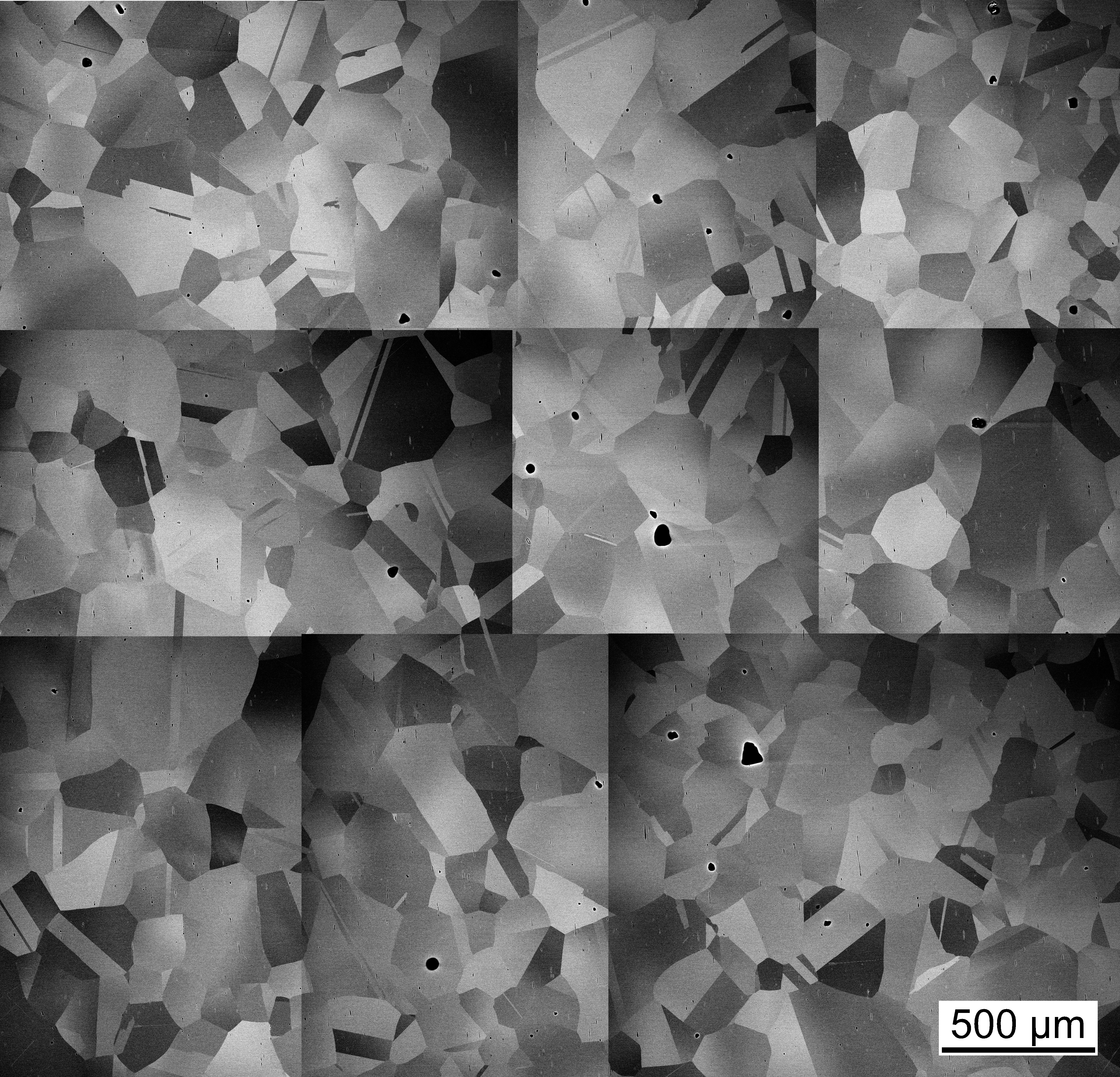

Supplement: Multimedia component 1 [file mmc1.zip › MnFeNi_1373K_60min/MnFeNi_1373K_60min_Montage.jpg]

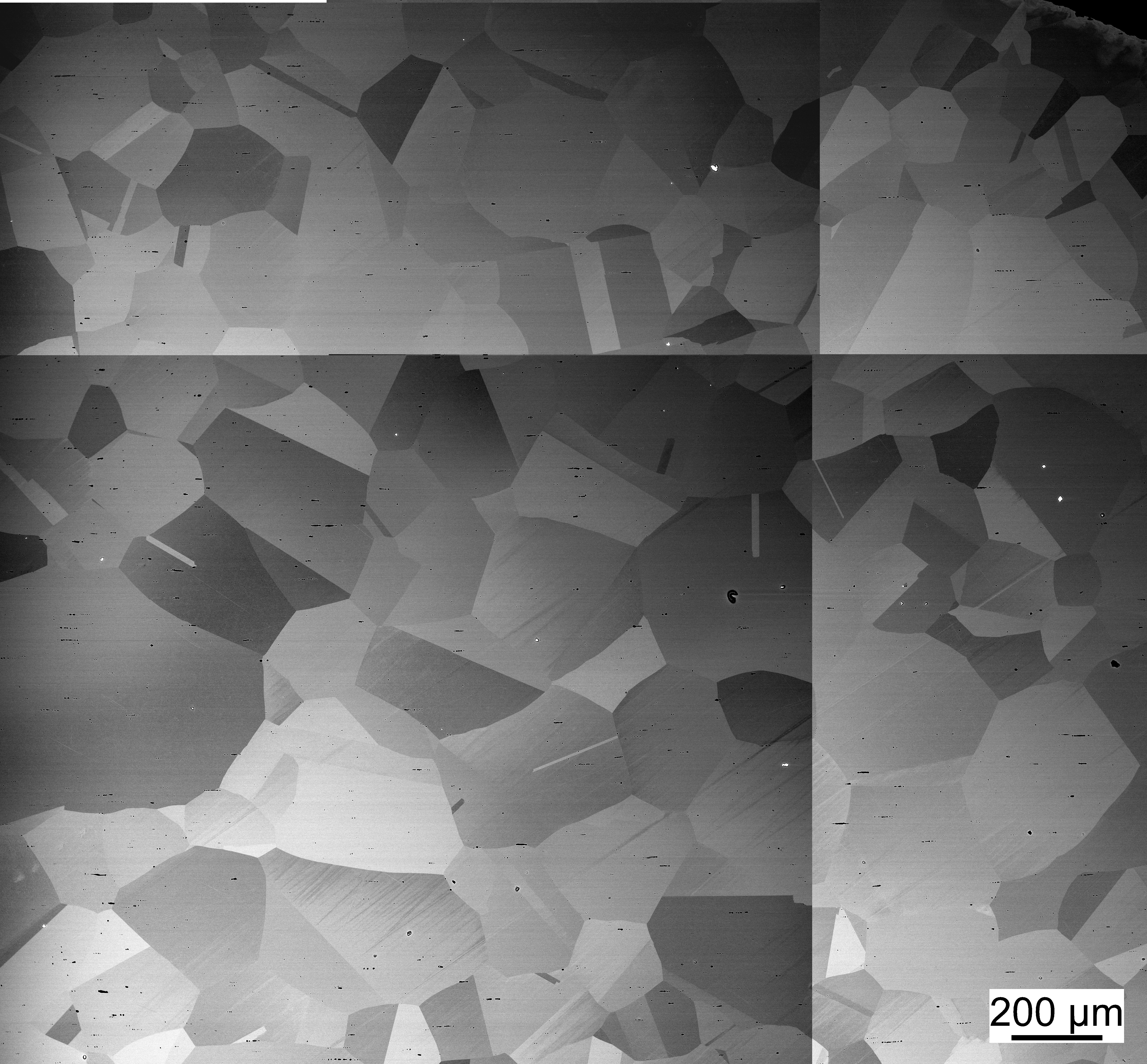

Supplement: Multimedia component 1 [file mmc1.zip › MnFeNi_1473K_60min/MnFeNi_1473K_60min_Montage.jpg]
